# A Self-Decoupling Multimodal Sensor for Enhanced Early Warning of Lithium-Ion Battery Thermal Runaway

**DOI:** 10.34133/research.1120

**Published:** 2026-02-24

**Authors:** Zhenglin Li, Meiyuan Jiao, Ke Chen, Yangyang Gao, Yang Gao, Cheng Lian, Jianrui Zhang, Fuzhen Xuan

**Affiliations:** ^1^ School of Mechanical and Power Engineering, Shanghai Key Laboratory of Intelligent Sensing and Detection Technology, East China University of Science and Technology, Shanghai 200237, China.; ^2^ Key Laboratory of Pressure Systems and Safety, Ministry of Education, East China University of Science and Technology, Shanghai 200237, China.; ^3^ State Key Laboratory of Chemical Engineering, Shanghai Engineering Research Center of Hierarchical Nanomaterials, School of Chemical Engineering, East China University of Science and Technology, Shanghai 200237, China.; ^4^ School of Chemistry and Molecular Engineering, East China University of Science and Technology, Shanghai 200237, China.

## Abstract

Lithium-ion batteries (LIBs) are central to sustainable energy systems but are vulnerable to thermal runaway (TR), necessitating robust safety monitoring. Conventional single-sensor systems cannot decouple the coupled electrochemical, thermal, and mechanical processes, while existing multimodal sensors suffer from signal cross-talk and large footprints in practical applications. Here, we introduce an insect-inspired, self-decoupling multimodal sensor, fabricated via maskless laser direct writing, that leverages distinct sensing mechanisms and orthogonal output signals for simultaneous strain, temperature, and gas detection. The sensor achieves reliable intrinsic decoupling of strain and temperature over 20 to 110 °C, along with an independent gas response. Seamlessly integrated onto lithium iron phosphate (LiFePO_4_) cells, it captures real-time multimodal data during both normal and TR events. Coupled with a bespoke multiphysics model, this platform reconstructs the thermal–mechanical evolution of LIBs. Our work provides a compact and durable strategy for precise real-time monitoring and early warning of battery failure.

## Introduction

Lithium-ion batteries (LIBs), endowed with high energy density, long cycle life, and eco-friendly recyclability, are indispensable to the global sustainable energy transition and are extensively deployed in electric vehicles, grid-scale storage systems, and consumer electronics [1]. However, under thermal, mechanical, or electrical abuse, these cells can trigger irreversible exothermic chain reactions—thermal runaway (TR) [2] process driven by internal chain exothermic reactions under abuse conditions, such as solid electrolyte interphase (SEI) decomposition and separator melting [3,4], is influenced by state of charge (SOC), and high charge/discharge rates markedly increase its hazards [5,6]. While LIBs are often housed in confined spaces, the flammable gases and high-temperature particles rapidly released during TR can quickly accumulate or disperse [7,8]. If subsequently exposed to stimuli such as arc faults or collisions, these conditions can readily trigger fires, explosions, or severe structural damage [9]. To address this risk, beyond passive protection strategies such as developing novel flame-retardant materials and separators [10,11], establishing precise TR detection and early warning systems is crucial for enabling timely intervention and ensuring battery system safety [12].

Early investigations focused on single-modality sensing, tracking electrical parameters [13], surface temperature [14], pressure [15], or gas emissions [16] for LIB safety monitoring. Nevertheless, such single-modality monitoring approaches fail to correlate external signals with battery internal reactions, in which electrochemical reactions, heat generation, and mechanical stress interact in complex ways, limiting their ability to comprehensively and accurately define the early warning window of TR [17].

To address these limitations, the concept of multimodal monitoring has emerged. This approach integrates multiple types of sensors to simultaneously monitor key physical parameters during LIB operation. Optical fiber sensors have been explored for LIB safety monitoring. For instance, Huang et al. [18] developed an optical fiber sensor with the capability of decoupling temperature and pressure signals, enabling real-time monitoring of the formation and structural evolution of the SEI layer. Mei et al. [19] integrated both a fiber Bragg grating and an open-cavity Fabry–Pérot interferometer onto a single optical fiber, leveraging their distinct sensitivities to temperature and pressure for intrinsic signal decoupling. However, their accuracy and long-term reliability are challenged during implantation and employment, where bending and vibrations can easily damage the sensors [20]. Moreover, the deployment of optical fiber sensors often relies on auxiliary equipment such as laser sources and fiber Bragg grating demodulators. The bulk and complexity of these systems largely hinder their applicability in practical industrial scenarios.

Thin-film multimodal sensors hold substantial promise for LIB monitoring owing to their superior flexibility, mechanical robustness, customizability, low cost, and portability. Fang’s group [21] developed a thin-film wireless multimodal sensor embedded inside LIBs for real-time state monitoring of strain and temperature. However, the decoupling of multimodal signals in their design does not originate from the intrinsic properties of the sensor itself but is achieved by placing a temperature sensor along a direction orthogonal to the principal strain. For real-time monitoring under electrical TR, Zhang et al. [22] developed a dual-modal sensor that leverages independent piezoresistive and thermoresistive mechanisms for simultaneous temperature and pressure detection.

Despite the aforementioned achievements, implementing multimodal monitoring strategies still presents considerable challenges due to the complex physical–chemical processes occurring within the LIBs. During charge–discharge or abuse conditions, electrode swelling and deformation, rapid temperature fluctuations, and the evolution of gases occur concurrently [23]. These interdependent parameters not only serve as critical indicators of LIB state but also introduce cross-sensitivity issues for multimodal sensors. This signal cross-talk impairs the accurate identification of individual stimuli, frequently requiring complex hardware and computational strategies for signal decoupling [24], thereby introducing considerable uncertainty into the estimation of the battery’s early warning window. As a result, combining multiple electrical signal types with orthogonal sensing mechanisms might be a promising approach to achieving robust and durable signal decoupling. Furthermore, most existing studies have focused on dual-modal sensing of temperature and pressure, which facilitates the identification of triggering events during TR in LIBs.

Here, inspired by the multimodal sensing capabilities of insects (e.g., bees) in perceiving force, temperature, and gas stimuli, we propose a self-decoupling multimodal sensor for enhanced early warning of LIB TR. Signal cross-talk is intrinsically minimized using distinct sensing mechanisms and output modalities, while response characteristics of each modal sensing unit are optimized and enhanced by tuning the composition of sensing materials. This design achieves reliable self-decoupling of strain and temperature signals across a broad temperature range (20 to 110 °C). Gas sensing is decoupled by compensating for independently measured temperature and strain signals. The sensor is fabricated using a cost-effective, maskless laser direct writing (LDW) process, enabling seamless integration of heterogeneous functional materials. As a proof of concept, the device was attached to a LiFePO_4_ LIBs to collect multimodal signals under both normal operation and various TR scenarios, including thermal, mechanical, and electrical abuse. By correlating the multimodal measurements with a multiphysics coupling model developed herein, the thermomechanical evolution of the LIB can be reconstructed. By enabling precise, real-time tracking of multiple physical parameters and their complex interplay during normal operation and TR, our work promises to substantially enhance the safety and reliability of energy storage systems.

## Results and Discussion

### State of art

**Fig. 1.**
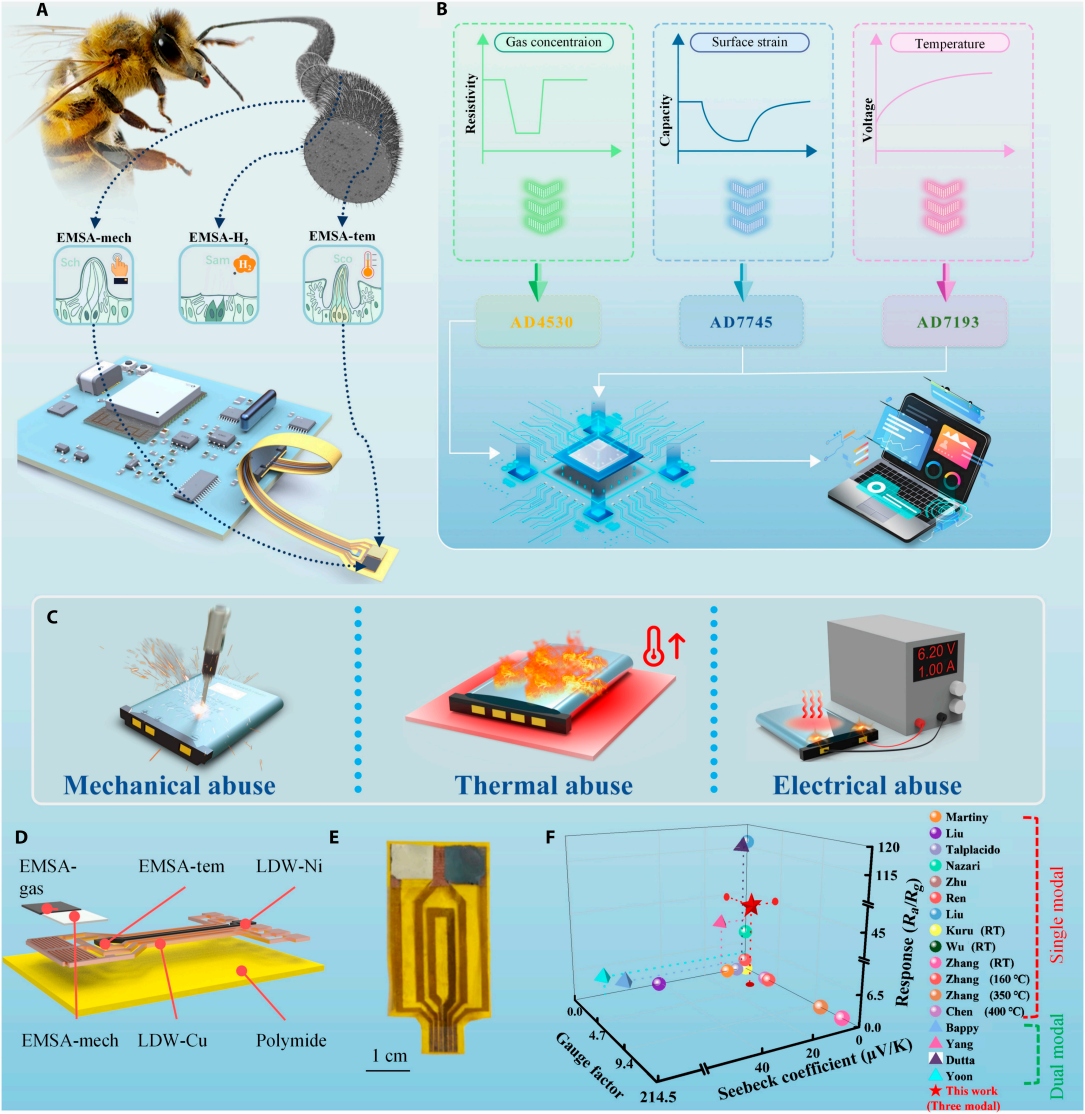
Schematic diagram of EMSA and its applications. (A) Schematic illustration of the electronic multifunctional sensing tentacle (EMSA) inspired by insect antennae. Sch, shaft-like for mechanical detection; Sam, basiconic for gas sensing; Sco, coeloconic for thermal sensing. (B) Signal modalities of the EMSA and architecture of the back-end data acquisition system. (C) Real-time monitoring of LIB TR under mechanical, thermal, and electrical abuse using the EMSA. (D) exploded view of EMSA. (E) A photograph of the EMSA. (F) Comparative analysis of the EMSA versus existing multimodal monitoring approaches for LIBs. RT, room temperature.

In nature, insects perceive complex environment through an integrated “sensilla–antennae–brain” system. Drawing inspiration from this biological system, we developed a multimodal flexible thin-film sensor system. The device comprises 3 functional components: highly sensitive and low-cross-talk sensing layers serving as the “sensilla”; an ultrathin, flexible substrate serving as the “antennae” to carry “sensilla”; and an integrated monitoring system serving as the “brain” for real-time data acquisition and processing. Drawing inspiration from insect antennae sensilla—shaft-like for mechanical detection, basiconic for gas sensing, and coeloconic for thermal sensing—we introduce the electronic multifunctional sensing antennae (EMSA), a thin-film, flexible multimodal sensor for LIB safety monitoring. The “antennae” of the EMSA is constructed from an 0.05-mm polyimide (PI) substrate and is equipped with various “sensilla” distributed across its tip, the fabrication process of which is shown in Fig. S1. Its “brain” is a customized back-end circuit that acquires, decouples, and analyzes capacitive, voltage, and resistive signals in real time, providing early failure warnings for LIBs (Fig. 1A and B). By designing electrode geometry to match pouch cell dimensions, EMSA reliably monitors strain, temperature, and ambient hydrogen across normal operation, mechanical, thermal, and electrical abuse TR (Fig. 1C). Figure 1D presents the structural configuration of the EMSA, comprising sensing units, substrate, and electrodes, while a photograph of the fabricated device is provided in Fig. 1E and Fig. S2A. Figure S2B presents measurements of the EMSA’s nonelectrode region before and after encapsulation, with values of 0.049 and 0.089 mm, respectively, reflecting the final thickness of the device. Alternative sizes and form factors (Fig. S2C) demonstrate EMSA’s adaptability for other applications. Figure 1F compares the performance of EMSA with previously reported LIB sensors. While most previous studies have been limited to dual-modal fiber-optic or thin-film sensors that couple temperature with strain or pressure, the EMSA represents the first reported device capable of true trimodal sensing by integrating gas detection with thermomechanical monitoring within a single, compact platform. Moreover, the EMSA demonstrates competitive single-mode performance in terms of strain, temperature, and gas sensitivities, underscoring its superior multifunctional capability [25]. Furthermore, Table S1 presents the fabrication cost of the EMSA and its comparison with commercial sensors, indicating that the proposed device exhibits promising cost advantage.

### Performance study of EMSA’s temperature sensing module

Under conditions such as overcharging, overdischarging, or mechanical impact, LIBs generate a large amount of heat internally, leading to a rise in temperature. When the temperature exceeds a certain threshold, a series of exothermic reactions will occur inside the battery, ultimately triggering TR. Therefore, temperature monitoring is extremely important for LIBs. The EMSA’s temperature sensing module (EMSA-tem) leverages the Seebeck effect for high-precision temperature measurement. This module features LDW Cu–Ni thermocouple junctions on a PI substrate [26]. Figure S3 presents the detailed characterization and analysis of the LDW-Cu/Ni electrodes.

**Fig. 2.**
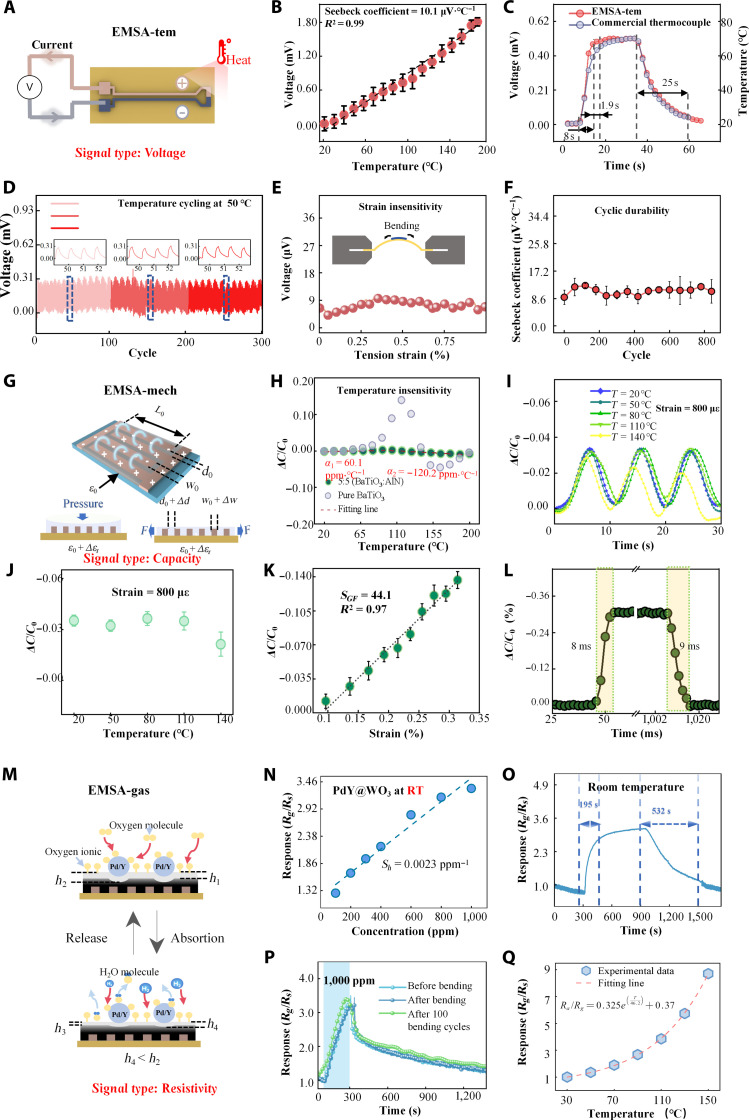
Comprehensive performance characterization of EMSA. (A) Response mechanism of EMSA-tem: Thermocouple sensor based on the Seebeck effect. (B) Seebeck voltage response of the device under 20 to 200 °C. (C) Response and recovery times of the device and commercial thermocouple. (D) Long-term temperature stability test of the EMSA-tem. (E) Response of the device to strain stimuli. (F) Variation in Seebeck coefficient of the device during multiple deformation cycles. (G) Response mechanism of EMSA-mech: Strain sensor based on the capacitance change of interdigitated electrodes. (H) Response of the device to temperature stimuli at 20 to 200 °C. (I) *ΔC*/*C*_0_ response under 150-με strain cycles at different temperature. (J) *ΔC*/*C*_0_ values corresponding to 150-με strain across the temperature range of 20 to 140 °C. (K) *ΔC*/*C*_0_ values at different strains. (L) Response and recovery times of the device. (M) Response mechanism of EMSA hydrogen: Redox reactions of semiconductor metal oxides. (N) Response of EMSA-gas to different hydrogen concentrations at room temperature. (O) The device response at room temperature at 1,000-ppm concentration of hydrogen. (P) Hydrogen response of the EMSA-gas under deformations. (Q) Response of the EMSA-gas under different temperatures.

According to the Seebeck effect, establishing a temperature gradient between one end of the copper–nickel junction and the other end of the electrode will generate a thermoelectric current and a Seebeck voltage, which is linearly related to the temperature difference (Fig. 2A). Figure 2B shows the thermoelectric response of EMSA-tem between 25 and 150 °C, a range that essentially covers the warning period of LIBs. The Seebeck coefficient (*S*_Seebeck_ = *∆V/∆T*) quantifies the device’s temperature sensitivity, where *ΔV* denotes the change in thermoelectric voltage from its baseline value and *ΔT* is the temperature differential across the thermocouple junction. The device has a *S*_Seebeck_ of 10.1 μV·°C^−1^, with excellent linearity (*R*^2^ = 0.99) and repeatability. Using a commercial infrared thermal imaging device as the reference standard, the sensing accuracy of the EMSA was evaluated to be ±1 °C (Fig. S4A) from 20 to 200 °C. When the device is contact with a 70 °C heat source, it responds within 8 s (Fig. 2C), exceeding the response time of a commercial thermocouple by about 1.9 s; after removal, the sensor stabilizes in ~25 s, comparable to the commercial sensor. The thermoelectric voltage of the EMSA-tem showed minimal deviation across a 3-week cycling test (Fig. 2D), demonstrating great long-term stability. To further assess stability under extreme conditions, we subjected the device to 1,000 rapid temperature cycles at 85% relative humidity (RH). As shown in Fig. S4B, the response remained essentially unchanged, demonstrating excellent robustness in high-humidity environments. Figure 2E illustrates the EMSA-tem’s voltage response to deformation-induced cross-talk. Across a 0% to 1% strain range, the output remains at the microvolt scale with negligible variation, confirming low strain sensitivity. After 1,000 cycles at 0.5% strain, the Seebeck maintains, further underscoring its long-term stability. In addition, the device was mounted on a horizontal heating stage and subjected to pressure from 0 to 40 kPa at 20 and 100 °C. The signals exhibit negligible pressure-induced cross-talk (Fig. S5).

### Performance study of EMSA’s mechanical sensing module

During the self-heating stage of TR in LIB [27], decomposition of the SEI layer induces trace gas generation, accompanied by a moderate casing microstrain in the range of 0 to 500 με [28]. As TR progresses, the LIB enters the accelerated TR stage [27]. In this stage, a rapid accumulation of heat and gas occurs inside the battery, leading to pronounced structural deformation, including casing bulging or even partial rupture. The casing strain increases markedly, reaching approximately 500 to 3,000 με [28]. Consequently, strain serves as a key parameter for identifying the onset of TR and discerning its developmental stage.

The EMSA’s mechanical sensing module (EMSA-mech) is integrated by LDW-fabricated interdigitated electrodes coated with a polydimethylsiloxane (PDMS) matrix loaded with barium titanate (BaTiO_3_) nanoparticles and aluminum nitride (AlN) nanoparticles (Fig. 2G). Each electrode is defined by its finger length (*l*_0_), thickness (*t*_0_), and interfinger gap (*d*_0_), with exact dimensions provided in Fig. S6A. The baseline capacitance (*C*_0_) is highly geometry-dependent exhibited by Eqs. (1) and (2)[29].

$$C=l_{0}\left(N-1\right)\frac{\varepsilon_{0}\varepsilon_{r,t}}{2}\frac{\left({\left(1-k^{2}\right)}^{1/2}\right)}{K\left(k\right)}$$ (1)

$$k=\text{cos}\left(\frac{\pi }{2}\frac{w_{0}}{d_{0}+w_{0}}\right)$$ (2)

where *N*, *ε*_0_, and *ε_r_,_t_* represent the total number of interdigitated fingers, the baseline relative permittivity of the vacuum, and the total relative permittivity surrounding the electrodes (including sensing layer, PI substrate, and air), respectively. *K*(*k*) denotes the complete elliptic integral of the first kind, accounting for fringe-field effects. Under uniaxial deformation (Fig. 2G), the interdigital gap increases by *Δd*, the width of each finger increases by *Δω*, and the relative dielectric constant of the sensitive layer changes by *Δε_r_*, resulting in a change in capacitance *C*. The device’s strain gauge factor (*S_GF_*) and pressure sensitivity (*S_P_*) are quantified by

$$S_{\mathrm{GF}}=\frac{\Delta C}{\varepsilon C_{0}}$$ (3)

$$S_{P}=\frac{\Delta C}{{\mathrm{PC}}_{0}}$$ (4)

where *ε* and *P* are the applied strain and the external pressure, respectively. BaTiO_3_ nanoparticles were selected for the sensing layer filler due to their high relative dielectric constant, which increases the sensor’s baseline capacitance and enhances its signal-to-noise ratio. Incorporating 30 wt % BaTiO_3_ nanoparticles into the PDMS matrix increases the initial capacitance 5-fold relative to the unfilled matrix (Fig. S6B). Raising the BaTiO_3_ content to 50 wt % produces only a 5.8% additional gain, suggesting the approached saturation of the filler loading in PDMS. Raman spectroscopy of tetragonal BaTiO_3_ phase (Fig. S6C) shows a prominent peak at 305 cm^−1^ [30]. Using the PDMS peak at 490 cm^−1^ as an internal standard, we derived the intensity ratio *I*_BaTiO3_*/I*_PDMS_ to semiquantify the change of BaTiO3 loading (Fig. S6D) [31]. Although this ratio increases with BaTiO_3_ concentration, it plateaus beyond 30 wt %, indicating that BaTiO_3_ dispersion in PDMS has reached a threshold. Subsequently, samples with BaTiO_3_ contents exceeding 30 wt % were characterized. Numerous agglomerates were observed distributed within the sensing layer (Figs. S6 and S7). Energy-dispersive x-ray spectroscopy (EDS) analysis further confirmed that these aggregates correspond to BaTiO_3_ nanoparticles (Fig. S6C and D). The presence of such uncontrollable agglomerates can compromise the stability and uniformity of the device. Therefore, the sample with 30 wt % BaTiO_3_ was selected for subsequent analyses. The capacitance of BaTiO_3_@PDMS composites first increases as temperature increases (Fig. S6E) and then decreases when the temperature reaches ~120 °C. This behavior arises from the opposing dielectric responses of the constituents: BaTiO_3_’s permittivity increases toward its curie temperature (~125 °C) [32], while PDMS’s permittivity monotonically decreases with temperature [33]. In addition, the maximum *ΔC/C*_0_ variation increases with higher BaTiO_3_ content. When the filler loading reaches 30 wt %, the capacitance changes at 120 °C is approximately 15%, introducing unacceptable thermal interference for practical applications.

To mitigate the pronounced thermal interference, we incorporated AlN nanoparticles, with stable relative permittivity up to 200 °C [34], into the composite matrix. Counterintuitively, the variation of *ΔC/C*_0_ with temperature first decreased and then increased as the AlN content rose (Fig. S5F), which may be attributed to the overall reduction in the composite’s relative permittivity with increasing AlN mass fraction, thus lowering *C*_0_ of EMSA-mech and amplifying the value of *ΔC*. To verify this, we measured the relative permittivity across different mass ratios. The relative permittivity indeed exhibited a decreasing trend with increasing AlN concentration, with a particularly sharp decline between the 6:4 and 10:0 ratios (Fig. S6G). Further support comes from the initial capacitance value (5.9 pF) of the EMSA-mech with 30% pure AlN (Fig. S6B), which is substantially lower than that of the 5:5 mass ration (7.8 pF), corroborating the proposed explanation. Therefore, a sensing layer containing 30 wt % total filler with a BaTiO_3_:AlN mass ratio of 5:5 was selected for the EMSA-mech. The scanning electron microscopy (SEM) characterization and analysis of 30 wt % EMSA-mech are presented in Fig. S8.

Figure 2H shows the temperature response of the EMSA-mech, which has a low sensitivity of 60.1 parts per million (ppm)·°C^−1^ within 20 to 100 °C, while its sensitivity increases to −120.2 ppm·°C^−1^ within 100 to 200 °C. Notably, within the early warning window for TR (20 to 100 °C), the optimized sensor exhibits a maximum *ΔC/C*_0_ change of only ~0.43%, corresponding to a strain of approximately 98.7 με, demonstrating excellent resistance to temperature-induced cross-talk. In contrast, the unoptimized PDMS/30% BaTiO_3_ composite shows a *ΔC/C*_0_ variation of up to 10% near 100 °C, confirming the enhanced thermal stability achieved through material optimization. Figure 2I shows *ΔC/C*_0_ response of EMSA-mech at various temperatures at 800 με. Results indicate nearly identical *ΔC/C*_0_ curves from 20 to 110 °C, whereas, at 140 °C, the baseline shifts markedly. This suggests that the module exhibits stable *ΔC/C*_0_ values across the temperature range of 20 to 110 °C (Fig. 2J), with strain sensitivity variations only occurring beyond ~110 °C; the temperature cross-talk performance of the sample in the range of 110 to 200 °C is shown in Fig. S9. Under an applied strain of 800 με, the *ΔC/C*_0_ value gradually decreases with increasing temperature, with strain sensitivity reaching its maximum attenuation of ~14.3% at 200 °C. Nevertheless, this temperature window (20 to 110 °C) fully meets the monitoring requirements for the early warning stage of LIB TR (60 to 100°C) [27]. Figure 2K presents the real-time *ΔC/C*_0_ response of EMSA-mech over a strain range of 0 to 3,500 με, effectively covering both the self-heating and accelerated stages of TR in LIBs [28]. The device demonstrates a notably high *S_GF_* of 44.1 and excellent linearity (*R*^2^ = 0.97), surpassing the performance of previously reported capacitive strain sensors [35]. The strain response accuracy of the EMSA-mech was evaluated to be ±3% within 0 to 4,000 με (Fig. S10). The EMSA-mech has response and recovery times of ~8 and ~9 ms (90 με), respectively, with a minimum detection limit of 30 με (Fig. S11A). The varying frequency (0.5 to 2 Hz) at 120-με strain has negligible impact on its performance (Fig. S11B). To assess the device’s long-term stability, we conducted cyclic bending tests within the 0- to 3,000-με strain range under common and extreme condition (Fig. S11C and D). After 10,000 cycles under common condition, peak capacitance and baseline shifts were ~3%. After 1,000 cycles under extreme condition (60 °C, 85% RH), the device response remains stable, demonstrating high durability. In addition, EMSA-mech also has the potential to serve as a pressure sensor. A detailed analysis of this aspect is presented in Fig. S12.

### Performance study of EMSA-gas module

During the early stage of TR in LIBs, gases such as hydrogen, carbon monoxide, carbon dioxide, and volatile organic compounds are released from the electrolyte. Among them, hydrogen is considered the most advantageous characteristic gas because it is generated through multiple pathways across various battery chemistries (LiFePO_4_, NCA, and NCM) and under diverse abuse conditions. It is released earlier than other gases [36] and has an extremely low background concentration in air, enabling reliable detection with minimal false alarms. Therefore, the hydrogen sensing unit EMSA-gas is integrated into EMSA, which senses hydrogen using a nanostructured tungsten oxide (WO_3_) layer modified with a Pd/Y alloy. Figure 2M depicts the hydrogen sensing mechanism. WO_3_ nanoparticles (n-type semiconductor) adsorb O_2_ molecules on their surface in air. These molecules capture conduction band electrons, forming O_2_^−^, O_2_^−^, and O_2_^2−^ ions and creating a depletion region of thickness *d*_1_, raising resistance. Pd/Y nanoparticles catalyze the dissociation of oxygen molecules into additional oxygen ions, expanding the depletion region to *d*_2_ (*d*_2_ > *d*_1_). When exposed to hydrogen, it reacts with surface oxygen ions, releasing electrons and forming H_2_O, thus lowering resistance. The Pd/Y spillover effect dissociates additional hydrogen into atoms, further reducing resistance and enhancing sensitivity and selectivity [37].

Figures S13 and S14 present the adsorption behavior of Pd/Y@WO_3_ toward hydrogen as calculated via first-principles density functional theory (DFT), along with a detailed analysis of the results. This process demonstrates the superiority of selecting the Pd/Y-alloy-modified WO_3_ in EMSA-gas. To validate DFT insights, we prepared Pd/Y@WO_3_, Pd@WO_3_, and WO_3_ nanoplates via identical protocols. The Pd/Y/WO_3_ mass ratio for Pd/Y@WO_3_ was 1:1:8, while the ratio for Pd@WO_3_ was 2:8. Figure S15 shows their responses to 1,000-ppm hydrogen at different temperatures. Pd/Y@WO_3_ exhibits stable sensitivity from room temperature to 75 °C. As temperature increases, the response performance improves, until the highest response reached at 100 °C. In contrast, Pd@WO_3_ shows highly unstable behavior with increasing temperature. Between 25 and 50 °C, the resistance decreases upon hydrogen introduction, but as the temperature exceeds 75°C, the resistance increases after hydrogen adsorption, and it does not return to the initial resistance once hydrogen input is stopped. Similarly, the WO_3_ nanoplates show irreversible and unstable hydrogen response in the temperature range of 25 to 75 °C, highlighting the excellent performance of Pd/Y@WO_3_. For further details on the structural and chemical characterization of the Pd/Y-loaded WO_3_ sensing layer, please refer to the Supplementary Materials (Figs. S16A to C and S17). The x-ray diffraction, x-ray photoelectron spectroscopy, SEM, and EDS analyses therein collectively confirm the successful formation of the composite material, with well-defined crystal phases, uniform elemental distribution, and a porous morphology conducive to gas sensing.

The ratio of the Pd and Y materials in the Pd/Y@WO_3_ sensitive layer markedly influences the response performance. The gas sensitivity *S_h_* is defined as *S_h_* = (*R_g_/R_s_*)/*C*_hydrogen_, where *R_g_* and *R_s_* are the initial and final resistance of the sensor, respectively, and *C*_hydrogen_ is the concentration of hydrogen. A device with a Pd/Y ratio of 5:5 provides the optimized hydrogen response, with an *S_h_* of 0.0023 ppm^−1^ at room temperature, higher than previous reports in LIB monitoring field [38]. The hydrogen response accuracy of the EMSA-gas is found to be ±10% (Fig. S18) over a wide concentration range, comparable to that of recently reported semiconductor-metal-oxide-based gas sensors [39]. Moreover, the response/recover time of the module at 1,000-ppm hydrogen is 195 and 532 s, respectively, faster than other Pd/Y ratios (Fig. 2O and Fig. S19B), leading us to select this ratio for further performance characterization. The gas sensing selectivity of the device was examined by measuring its responses to hydrogen, CO, CO_2_, NH_3_, and C_2_H_4_ at 1,000 ppm at room temperature. The device’s response to CO, CO_2_, NH_3_, and C_2_H_4_ are 1.02, 1.11, 1.12, and 1.09, respectively (Fig. S20A), all markedly lower than that to hydrogen, indicating a high selectivity of the sensor. The device has a lowest detection limit of 10 ppm at room temperature (Fig. S20B), exceeding the international standard (100 ppm; ISO-26142). The EMSA-gas was subjected to a 3-week stability test involving 6 cycles/week, demonstrating that a highly consistent response amplitude to 1,000 ppm hydrogen is maintained both within and across the weekly test periods (Fig. S20C), indicating its reliable long-term performance.

The temperature and strain cross-talk of the EMSA-gas was evaluated. Figure 2P shows that a radius of curvature of 15 mm produces a negligible change in its response to hydrogen. Over 100 bending cycles, the hydrogen response of the EMSA-gas increases by approximately 5%, which might be attributed to the formation of microcracks on the EMSA-gas surface, creating additional active sites for hydrogen adsorption [40]. Figure 2Q shows that the hydrogen response of the EMSA-gas decreases with increasing temperature, with a maximum of 7.1 at 150 °C. Figure S16D shows the minimal strain-induced cross-talk to EMSA-gas. Thus, for practical battery monitoring, the response of the EMSA-gas should be compensated with EMSA-tem and EMSA-mech signals.

In conclusion, the EMSA demonstrates robust and stable single-parameter responses, as well as excellent intersignal isolation within its multimodal framework. A comprehensive performance evaluation—including cross-talk quantification, response-time analysis, and benchmarking against both reported and commercial multimodal sensors—further validates its superior sensing precision and integration simplicity. Complete datasets, analytical details, and comparative results are presented in the Supplementary Materials (Tables S2 to S5).

### EMSA monitoring of LIB charging/discharging under normal operation

**Fig. 3.**
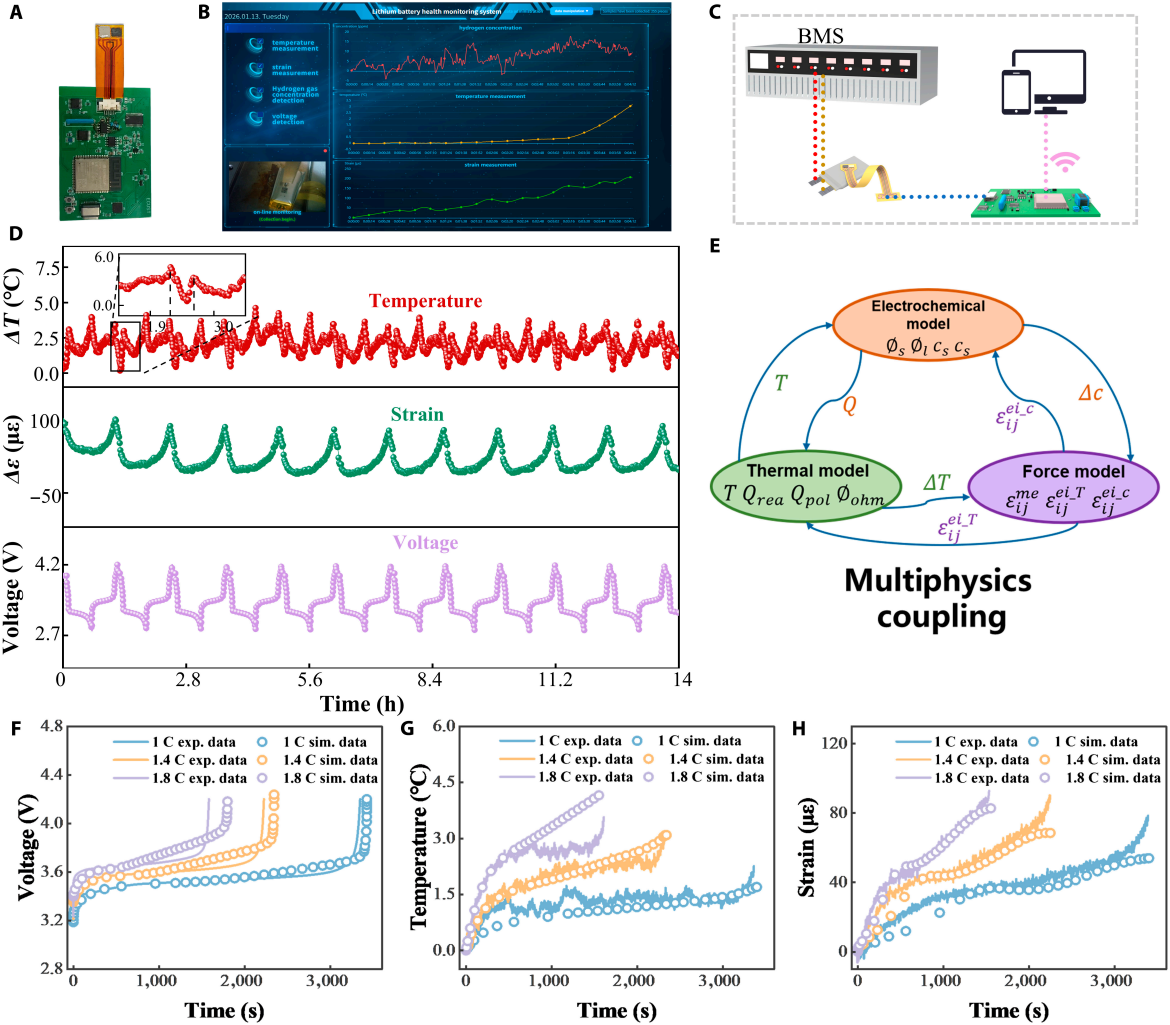
Multimodal monitoring of LIBs under normal operating conditions by EMSA. (A) A photograph of battery management system (BMS), EMSA, and the data acquisition module. (B) Signal acquisition software interface. (C) Experimental setup for online multimodal monitoring of a pouch-type LIB. (D) Voltage, strain, and temperature changes of LIBs under cyclic charging/discharging at a charging rate of 1 C. (E) E–T–M coupling model for pouch cell LIBs. Comparison of monitored (F) voltage, (G) temperature, and (H) strain signals with simulated ones under different charging rates of an LIB.

Conventional LIB monitoring systems often rely on complex backend signal acquisition setups, including fiber-optic demodulators, thermocouple readers, infrared cameras, or other customized modules [41]. These systems are typically bulky, functionally limited, and lack integration. In contrast, we designed and developed a compact multimodal data acquisition module based on the EMSA platform (Fig. 3A), along with its dedicated software interface (Fig. 3B). On the basis of this system, an experimental platform was configured for LIB monitoring under normal operation condition (Fig. 3C). The EMSA was bonded to the surface of a 350-mA·h LiFePO_4_ pouch cell and interfaced with our multimodal data acquisition module. A battery management system controlled the operating parameters, and all measurements were streamed wirelessly to a PC. Since no external hydrogen evolution occurs under normal conditions, we concentrated on monitoring surface strain and temperature variation during LIB charging/discharging cycles.

The EMSA was firstly calibrated against a commercial thermal infrared camera and strain gauges at 1 C (Fig. S21A), demonstrating small temperature and strain variations between EMSA and commercial sensors (Fig. S21B and C). Under room-temperature charge–discharge cycling (Fig. 3D), the LIB’s temperature profile displayed 2 distinct peaks per cycle—charging and discharging (inset of Fig. 3D). This behavior reflects the exothermic nature of both charge and discharge, coupled with a 30-s rest period between them that causes a postpeak temperature dip, followed by a secondary rise. The charging peak is approximately 0.8 °C higher than the discharging peak. Strain responses are remarkably consistent, peaking at ~110 με coincident with the end of charge. Figure S22 summarizes how surface peak strain, temperature, and LIB capacity evolve under different charge rates. Peak strain increases over the first 9 cycles and then plateaus. A clear correlation emerges between strain and capacity, both of which scale with charge rate. Similarly, increasing from 1 to 2 C elevates the peak temperature by approximately 1.8 °C. This phenomenon arises because higher cycle count and faster rates hinder uniform Li^+^ transport, triggering Li plating [42], capacity fade, and increased internal resistance [43], which, in turn, elevate maximum strain and temperature.

To achieve reliable early failure warning, a model must capture not only electrochemical kinetics but also the interplay of thermal and mechanical effects—something beyond the reach of single-physics frameworks. Therefore, we developed an electrochemical–thermal–mechanical (E–T–M) coupling model (Fig. 3E), whose comparison with experimental data can inform early failure warning. The detailed structure and coupling mechanisms of the E–T–M model are provided in the Supplementary Materials (Fig. S23). Model accuracy was validated by directly comparing simulations with EMSA-acquired real-time measurements. Figure 3F shows that simulated charge profiles at various C rates follow the experimental data closely. Figure 3G presents the simulated and experimental temperature curves of the LIB, which initially rises, then falls, and rises again. With deeper discharge, elevated Li^+^ concentration in the cathode impedes intercalation, increasing internal resistance and heat generation. Natural convection (*h* = 0.5 W·m^−2^·K^−1^) was incorporated to explain the transient temperature drop, where heat dissipation temporarily exceeds generation. As charging proceeds, heat generation again dominates, leading to renewed temperature rise. At higher C rates, the temperature curve approaches linearity, highlighting the dominance of ohmic heating. Figure 3H shows good agreement between simulated and experimental surface strain at different C rates. To validate the generality of the model across different battery chemistries and capacities, we selected an 850-mA·h LMO battery was selected for charge–discharge tests under various C rates. After adjusting the relevant material parameters in the model, the simulated results were compared with experimental data, as shown in Fig. S24. The close agreement demonstrates that the model maintains good predictive accuracy for LMO batteries as well, reflecting its strong versatility. Together, these findings confirm the accuracy of our E–T–M coupling model, offering in depth insight into battery dynamics and underpinning EMSA enabled multimodal monitoring.

### EMSA monitoring of LIB under TR

LIB TR is typically initiated by thermal, mechanical, or electrical abuse, posing a critical safety challenge for electric vehicles, grid-scale storage, and portable electronics. Existing early warning strategies predominantly rely on single-mode indicators, such as characteristic TR temperatures (*TR*_1_ to *TR*_3_) [44], pressure spikes [19], or abnormal electrical signals. Small-capacity cells in the *TR*_1_ to *TR*_2_ range often exhibit subthreshold failures involving gas release, lithium plating, and swelling—degrading performance and concealing latent risks. Moreover, commercial protection circuitry, while critical for safety, masks electrical signatures during mild TR, limiting the usefulness of electrical monitoring. In this study, we examine noncombustion TR in protected, small-capacity LIBs under 3 abuse conditions, linking multimodal mechanisms to sensor outputs to develop an effective early warning framework.

**Fig. 4.**
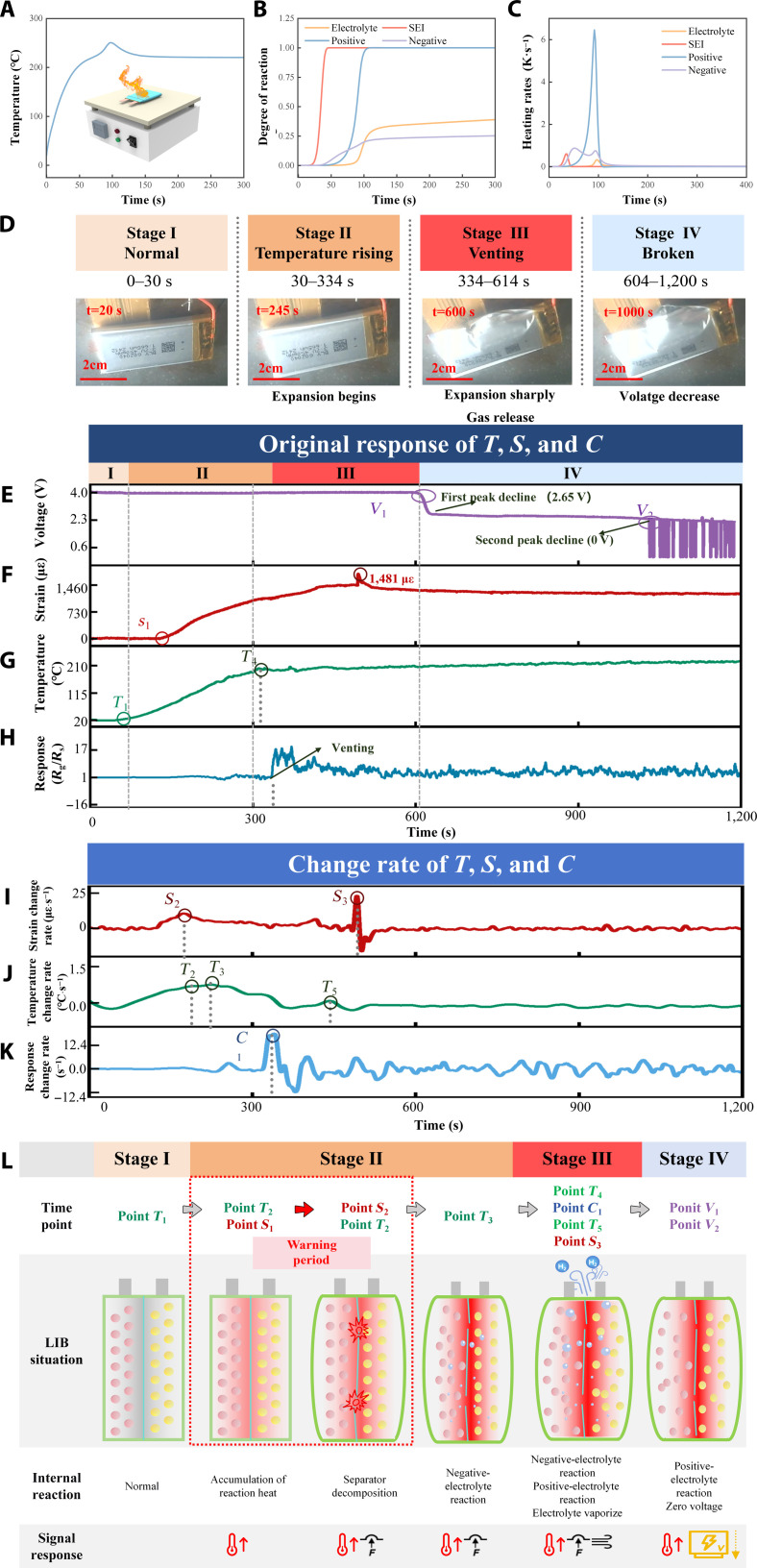
EMSA-based multimodal monitoring of LIBs under thermal abuse TR. (A) Simulated temperature profile during thermal abuse TR based on the E–T model. (B) E–T model prediction of the sequence and degree of internal reactions in LIBs during TR. (C) Simulated heat generation rates for each component reaction. (D) Photographs of an LIB at critical reaction stage during thermal abuse TR. (E) In situ voltage signals of an LIB during thermal abuse TR. In situ multimodal signals of (F) strain, (G) temperature, and (H) hydrogen captured by EMSA during thermal abuse TR. Temporal derivatives of (I) strain, (J) temperature, and (K) hydrogen response from EMSA data. (L) sequence of model-predicted internal reactions aligned with key multimodal signal time points, delineating the thermal abuse TR early warning interval.

In this study, we first established an electrochemical–temperature–pressure (E–T–P) TR model of the LIB to quantify the heat released from chemical reactions under elevated temperatures (Fig. S25). Using this E–T–P model, the evolution of the TR behavior of LIBs under heating conditions is constructed (Fig. 4A). The TR progression, modeled in Fig. 4B and C, occurs in distinct stages marked by different heat-release characteristics: initial SEI decomposition (low heat), followed by negative- and positive-electrolyte reactions (increasing heat), and finally vigorous gas generation from electrolyte decomposition and boiling. This gas generation and pressurization process is further illustrated in Fig. S26. The internal battery pressure gradually increases from an initial zero value. By monitoring pressure changes, the model can accurately identify the onset of gas release from SEI decomposition. Subsequently, electrolyte decomposition causes a sharp increase in gas production, leading to rapid pressure rise. Once the internal pressure reaches the battery’s pressure threshold, the gas is released outward. An EMSA-based multimodal monitoring system was deployed on a 50% SOC pouch cell with an external safety circuit under thermal abuse. The EMSA, attached to the cell inside a sealed chamber, wirelessly transmitted sensing data via WiFi, while an internal camera recorded the TR process. Notably, real-time temperature compensation was applied to the hydrogen response for qualitative detection. The entire TR process, divided into 4 key stages (I: normal operation, II: temperature rise, III: gas release, and IV: failure; Fig. 4D), is shown in Movie S1. Figure 4E to K presents EMSA-recorded multimodal signals and their temporal derivatives, while Fig. 4L illustrates the TR reaction mechanisms in LIBs along with key time markers. In stage I (0 to 30 s), the pouch cell remains stable with no observable change. Stage II begins at *T*_1_ ≈ 34.5 s. Heat accumulation triggers a minor strain increase at *S*_1_ (114.5 s, ~49 °C; Fig. 4F), well before the visual detection thresholds time (214 s). At *S*_2_ (*t* = 174 s; ~91.1 °C), the strain rate reaches maximum (~10.5 με·°C^−1^; Fig. 4I), whose temperature is consistent with SEI decomposition temperatures reported by Wang et al [45], corroborating the mechanism illustrated in Fig. 4L. Notably, the temperature rise rate at this moment reaches 0.94 °C·s^−1^, occurring approximately 9 s prior to the commonly reported TR onset threshold of 1 °C·s^−1^ [46] (defined as the *T*_2_ point at ~183 s; Fig. 4G). As TR progresses, the temperature-rise rate reaches its maximum (1.12 °C·s^−1^) at *T*_3_ (224 s, 143 °C; Fig. 4J) with strain ≈ 701 με and visible swelling (stage II; Fig. 4D), marking the onset of positive electrolyte reactions. The surface temperature peaks again at *T*_4_ (319 s, 209 °C), hinting at further anode processes. Electrolyte evaporation between *T*_4_ and *C*_1_ (333.9 s; Fig. 4H) drives a hydrogen surge, as confirmed by the EMSA-gas signal. Subsequent local peaks at *T*_5_ (441 s, 118°C) and *S*_3_ (481 s, ~1,481 με) reflect complete negative electrolyte decomposition and ongoing low-severity positive electrolyte reactions without major thermal spikes. In contrast, the voltage remains stable under the safety circuit until *V*_1_ (601.5 s) and then collapses to zero at *V*_2_ (1,028 s), indicating full failure. The onset of SEI film decomposition is widely recognized as the critical threshold for irreversible TR in batteries. Accordingly, conventional single-parameter approaches define the early warning window as spanning from *T*_1_ to *T*_4_. However, when incorporating insights from both internal reaction pathways and multimodal signal analysis, the EMSA-based monitoring strategy enables this window to be further narrowed to the *T*_1_–*S*_2_ interval, offering earlier and more precise TR detection.

**Fig. 5.**
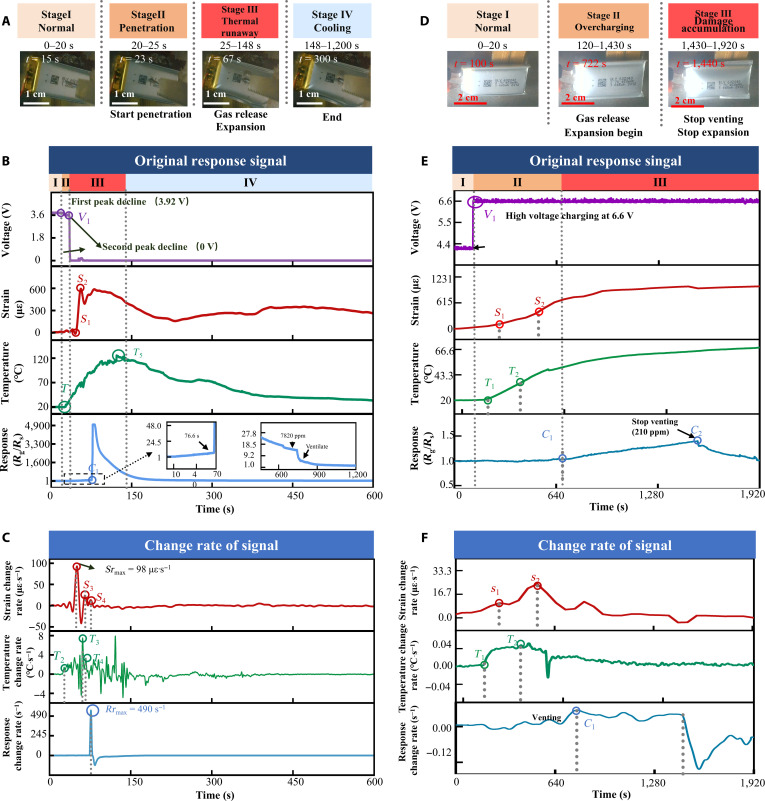
EMSA-based multimodal monitoring of LIBs under mechanical abuse and electrical abuse TR. (A) Photographs of an LIB at critical reaction stage during mechanical abuse TR. (B) In situ multimodal signals captured by EMSA during mechanical abuse TR. (C) Temporal derivatives of strain, temperature, and hydrogen concentration from EMSA data during mechanical abuse TR. (D) Photographs of an LIB at critical reaction stage during electrical abuse TR. (E) In situ voltage signals of an LIB during electrical abuse TR. (F) Temporal derivatives of strain, temperature, and hydrogen concentration from EMSA data during electrical abuse TR.

The mechanical abuse test was performed using a microcontroller-driven motor to penetrate a fully charged LIB with a 2-mm-diameter stainless steel nail at a constant speed of 0.5 mm·s^−1^ (Fig. S27A). Images before and after the needle-puncture TR test are shown in Fig. S27B and C. Prior to testing, the cell was connected to an external safety circuit to simulate realistic operating conditions. As shown in Fig. 5A to C and Movie S2, the TR process induced by nail penetration and the corresponding multimodal signals can be divided into 4 distinct stages: I: normal operation, II: penetration, III: TR, and IV, cooling. The internal reaction flow is illustrated in Fig. S27D. The battery remained in a stable operating state during the initial 0 to 20 s (stage I). At *t* = 20 s (stage II), the penetration of the nail led to internal short-circuiting and localized heat generation, a process lasting approximately 6 s. Among the multimodal response signals, the output voltage exhibits the earliest response, dropping sharply from 3.7 to 3.51 V at 22.8 s and reaching 0 V at the *V*_1_ time point (29.2 s). The localized short circuit generated substantial joule and entropic heat, which began to propagate throughout the battery. The temperature at *T*_1_ (26.3 s) starts to climb sharply—2.9 s before the voltage falls to zero. We avoid comparing this point to the voltage disturbance at 22.8 s, since voltage blips can also result from voltage sensor faults or sampling jitter [47]. Continued internal heat accumulation caused the battery’s subsequent thermal behavior to closely mirror that observed under thermal abuse conditions [48], thus validating the use of previously established thermal abuse models to analyze the heat response under mechanical abuse scenarios. In addition, a slight fluctuation in strain was observed at 21.5 s, which is attributed to surface deformation caused by nail penetration. As heat accumulated, the temperature rise rate peaked at *T*_2_ (26.9s), reaching 1.32 °C·s^−1^—exceeding the critical threshold of 1 °C·s^−1^. At *S*_1_ (43.1 s), the strain sharply increased at a rate of 90.6 με·°C^−1^, coinciding with a surface temperature of ~99 °C—indicative of vigorous decomposition of the SEI film and associated gas and heat release. The strain peaked at *S*_2_ (55.4 s) and then rapidly declined, likely because of the escape of electrolyte and gas through the puncture site, as shown in Movie S2. A second peak in temperature rise rate (~7.5 °C·s^−1^) occurred at *T*_3_ (61.1 s), attributed to exothermic reactions involving the anode electrolyte. This was followed by strain and temperature surges at *S*_3_ (65.9 s) and *T*_4_ (66.8 s), linked to continued electrolyte reactions and boiling. At *C*_1_ (76.6 s), a substantial hydrogen release was detected, showing a markedly stronger signal than in thermal-abuse-induced TR. Notably, the strain began to decline at this stage, possibly due to the depletion of active materials and venting of internal gases and electrolyte. The hydrogen signal dropped quickly after peaking, attributed to its diffusion from the EMSA region into the chamber, stabilizing at ~7,820 ppm around 760 s and falling to zero after venting. At *T*_5_ (121.94 s), the TR temperature reached a maximum of ~122 °C before entering the cooling stage (stage IV). Mechanically induced TR is thus characterized by rapid onset, high intensity, and a short warning window. Voltage signals offer the most immediate indication of abuse onset, while EMSA-based multimodal sensing enables more accurate tracking of TR evolution. We define the warning period as the interval from *V*_1_ to *T*_2_ (from initial mechanical abuse to SEI decomposition) and the danger period from *T*_3_ to *C*_1_, during which hazardous gas release, thermal escalation, and expansion create a high combustion risk.

The experimental setup for electrical abuse testing is shown in Fig. S28A. To eliminate interference from safety circuits during overcharging, we directly connected the fully charged LIB (100% SOC, identical to previous tests) to a dc power source. As shown in Fig. 5D to F, the overcharge-induced TR process was divided into 3 stages: I: normal, II: overcharging, and III: damage accumulation. From 0 to 120 s, the voltage was set to 4.2 V, corresponding to the nominal full-charge voltage of commercial pouch cells. At 120 s, the voltage was raised to 6.6 V, initiating stage II. The battery surface temperature began to rise at *T*_1_ (125 s), while strain remained relatively stable. A minor local peak in strain rate (160 με) was observed at *S*_1_ (293 s), reflecting mild expansion likely within the reversible reaction range, owing to electrode design redundancy. At *S*_2_ (418 s), the strain growth rate began to increase again. Although the temperature rise rate peaked at *T*_2_ (438 s), it remained below 1 °C·s^−1^, falling short of the early warning threshold. At *S*_3_ (493 s), the strain rate reached its maximum, indicating intensified internal reactions. On the basis of the typical overcharge mechanism [49], TR likely started at *S*_2_, accompanied by lithium plating, SEI growth, and other side reactions that generated joule heat and caused volumetric expansion, peaking at *S*_3_. This is further supported by the visible surface bulging shown in stage II of Fig. 5D. Figure 5D visually confirms noticeable surface bulging. Hydrogen release was detected by EMSA-gas at *C*_1_ (722 s), with the concentration gradually increasing, indicating onset of SEI and electrolyte decomposition with gas generation and slow venting. At *C*_2_, hydrogen concentration reached a maximum before declining, suggesting reduced reaction intensity. The *C*_1_*–C*_2_ interval reflects battery loss of internal sealing and a substantially elevated safety risk. As shown in Fig. S21B, posttest cell images reveal visible swelling but no surface rupture. Throughout the experiment, the LIB surface temperature remained between 20 and 70 °C (Fig. 5E) with no abrupt temperature rise, indicating the absence of TR. However, the EMSA detected clear battery swelling and hydrogen leakage in Fig. 5E, confirming damage to the battery cell. Further analysis reveals a distinct reaction mechanism under electrical abuse. As shown in Fig. S28D, the strain variation rate peaked at stage II (*S*_2_). The observed swelling was not primarily caused by heat accumulation from electrolyte reactions but rather by lithium metal deposition at high potentials during overcharging. This deposited lithium reacts with other components, generating gases such as hydrogen and ultimately leading to failure [50]. This highlights a limitation of relying solely on temperature sensors due to internal–external thermal gradients, which hinders timely damage detection. On the basis of the above, we define the warning period for overcharge-induced TR as the interval between *S*_2_ and *C*_1_, with the strain rate peak marking the damage accumulation stage trigger point and hydrogen release indicating cell failure. These results demonstrate the effectiveness of EMSA-based multimodal sensing in real-time TR monitoring.

Thermal, mechanical, and electrical abuse tests confirm that EMSA provides noninvasive, comprehensive, and accurate monitoring of temperature, strain, and hydrogen concentration. Compared to single-parameter methods, multimodal analysis narrows early warning windows and refines thresholds, enabling intervention before catastrophic TR events. Figure S29 compares EMSA’s TR monitoring strategies under thermal, mechanical, and electrical abuse with traditional voltage- and vision-based methods. In Fig. S29A, EMSA defines an early warning window for thermal abuse TR, from strain onset to peak strain rate (114.5 to 174 s), nested within the temperature-rise interval (34.5 to 183 s at 1 °C·s^−1^), enhancing diagnostic confidence. By contrast, EMSA-gas detects hydrogen release at 333.9 s, marking packaging rupture and impending failure. The interval from 333.9 to 1,200 s defines the damage phase. In contrast, voltage drop appears at 601.5 s, while visible swelling occurs only at 304 s, highlighting the limitations of voltage- and vision-based diagnostics for early TR detection. The window between early warning and damage (174 to 333.9 s) constitutes the TR phase.

Figure S29B depicts the time evolution of mechanical abuse TR. Under this condition, EMSA-tem exhibits the fastest response, with the temperature ramp rate exceeding 1 °C·s^−1^ at 26.9 s, resulting in a narrow early warning window of just 1.9 s—still approximately 2.3 s ahead of the voltage drop. Figure S28C shows that under overcharge conditions, the reaction degree is relatively mild and may not necessarily trigger TR. The multimodal signal changes (strain and hydrogen gas) are mainly caused by internal lithium deposition. At this time, both voltage and temperature exhibit minor fluctuations, and the temperature rise rate is always below 1 °C·s^−1^, making it impossible to detect the reaction through temperature parameters. Referencing the earlier E–T–M predictive model, the interval between the strain surpassing the simulated peak and the peak in strain rate is defined as the TR early warning phase (293 to 493 s)—approximately 48 s earlier than the visually observable changes, which occur at 541 s. hydrogen release is further used to demarcate the point of complete battery failure.

A comprehensive comparison of multisource sensing data across all abuse scenarios reveals that mechanical abuse induces the most intense TR response, characterized by the shortest early warning window, highest hydrogen release, and earliest gas evolution. In both thermal and electrical abuse, hydrogen release consistently lags behind the temperature peak. The key distinction between these 2 lies in the rate of heat generation, with electrical abuse showing a more gradual temperature increase. These results highlight the advantages of EMSA in narrowing the TR early warning window, identifying onset stages, and distinguishing between TR types. Hence, EMSA holds promise not only for real-time safety diagnostics at the user level but also for guiding safety-informed battery design during development level.

## Conclusion

In this study, we present a flexible, customizable, and highly integrated multimodal thin-film sensor—EMSA—with low-signal cross-talk, along with a dedicated data acquisition module for real-time wireless monitoring of strain, temperature, and hydrogen concentration during LIB TR. Leveraging distinct sensing mechanisms, EMSA achieves self-decoupling of temperature and strain measurements within the range of 20 to 110 °C. Decoupled gas response can be achieved through the compensation of independent temperature and strain signals.. The proposed EMSA system enables comprehensive monitoring of LIBs during normal operation and under thermal, mechanical, and electrical abuse, effectively capturing data throughout TR events.

We further developed a multiphysics-coupled model tailored for LiFePO_4_ pouch cells, which accurately predicts battery surface temperature and strain evolution across varying charging rates. When integrated with EMSA sensing data, the model facilitates precise evaluation of battery operational status. Notably, as TR initiates, EMSA offers enhanced, multidimensional signal information that surpasses traditional single-parameter indicators. By analyzing both multimodal signal trajectories and their derivatives, EMSA enables earlier and more reliable identification of critical thresholds during the latent phase of TR, allowing for timely intervention before the onset of irreversible cascading reactions, thus substantially improving battery safety.

Moreover, when combined with conventional voltage monitoring, EMSA can help distinguish between different TR triggers, supporting targeted preventive strategies and active mitigation measures. Its capability to infer internal reaction dynamics under specific TR scenarios suggests strong potential for integration during the battery design stage. Coupled with TR modeling, EMSA can contribute to safety-oriented optimization and risk assessment in battery manufacturing processes.

## Materials and Methods

### Preparation Cu electrodes of EMSA

A 0.05-mm-thick PI substrate was first treated with ozone–ultraviolet to enhance its hydrophilicity. Following our previous work [26], a copper–salt solution was rod-coated onto the PI. A continuous-wave laser (Shenzhen 91 Laser Co. Ltd.; 405 nm, 7 W, 50-μm beam diameter, and scan speed of 10 mm·s^−1^) was then used to irradiate the surface along a preprogrammed CAD pattern, forming the Cu electrodes. Finally, the substrate was rinsed with alcohol to remove residual salts and dried at 50 °C for 2 min.

### Preparation of EMSA-tem module

NiO nanoparticles were first synthesized as reported [51]. A precursor slurry of NiO, polyvinyl pyrrolidone, and *n*-pentanol (24:5:71 mass ratio) underwent 6-h sonication and was rod-coated onto a Cu-patterned PI substrate. Electrodes and Cu–Ni junctions were then formed by LDW using a 355-nm pulsed laser (MMEPU-355-5, Meiman Laser Technology Co. Ltd.) at 20 kHz, 50 mW, and 50 mm·s^−1^. Finally, the film was alcohol-rinsed to remove residues and dried at 50 °C on a hot plate.

### Preparation of EMSA-mech module

Five grams of BaTiO_3_ (100 nm; MERYER) were dispersed in 250 ml of ethanol–water (95:5, v/v) solution. After adding 2.5 g of 3-aminopropyltriethoxysilane, the suspension was magnetically stirred for 24 h to complete hydrolysis and surface functionalization. The modified particles were collected by filtration and dried at 60 °C for 1 h. A composite precursor was prepared by mixing the functionalized BaTiO_3_, AlN nanoparticles (500 nm; Zhongye New Materials Co. Ltd.), and PDMS at a 1.5:1.5:7 mass ratio. The resulting colloid was cast onto the LDW-patterned PI substrate and cured at 80 °C for 2 h to form the EMSA-mech module.

### Preparation of EMSA-gas module

The EMSA-gas sensing layer was prepared by dispersing 0.1865 g of WO_3_ nanoparticles in 10 g of a water–ethanol solvent (1:1 by mass). Subsequently, 0.0196 g of YCl_3_ and 0.0177 g of PdCl_2_ were successively added with 15 min of ultrasonication. The mixture was heated to 80 °C to remove the solvent. The precursor was then calcined in a tube furnace under argon at 650 °C for 2 h using a rapid moving bed pyrolysis method, where the quartz boat was swiftly inserted into the hot zone in 1 s. This process enabled the mixed-metal precursors to undergo simultaneous thermal decomposition at elevated temperatures, promoting the formation of a highly supersaturated state. Consequently, fine and uniformly dispersed Pd/Y alloy nanoparticles were formed on the WO_3_ support. Upon natural cooling, alloyed nanoparticles were recovered. A coating solvent—composed of pine oil alcohol, butyl carbitol acetate, and dibutyl phthalate (60:30:10 wt %)—was prepared. The nanopowder was milled into this solvent at a 1:5 powder-to-solvent ratio, applied to the device, and dried at 100 °C for 10 min to evaporate the solvent.

### Characterization of the EMSA sensitive material

The surface morphology was characterized by field-emission SEM (S-4800, Hitachi). Phase purity and chemical composition were analyzed using micro-Raman spectroscopy (Renishaw Invia; 514-nm laser), EDS (S-4800, Hitachi), and powder x-ray diffraction (D/MAX-2550 VB/PC, Rigaku).

### Performance study of the EMSA

The Seebeck voltage of EMSA-tem was determined indirectly by measuring the current across a calibrated 20-kΩ resistor (CHI660E electrochemical workstation), while temperature was controlled with a hot plate (C-MAG HS7, IKA). The strain sensing performance of EMSA-mech was assessed using a MARK-10 horizontal tensile tester, with capacitance shifts recorded by an LCR meter (3511-50, HIKOKI) at 10 kHz. The hydrogen sensing behavior of EMSA-gas was tested in a custom sealed chamber with controlled hydrogen flow; resistance changes were monitored using a Keithley 4200A-SCS parameter analyzer, and the operating temperature was regulated by an integrated heating stage.

### Monitoring of LIBs by EMSA at normal operation condition

A 350-mA·h LiFePO_4_ pouch cell (no external safety circuit) was interfaced with a battery management system (CT9004, NEWARE Technology Limited) to execute charge–discharge protocols at designated C rates. The EMSA was bonded to the cell surface with cyanoacrylate adhesive (Pattex), and its flexible printed circuit connector was linked directly to the data acquisition module for wireless real-time monitoring.

### Monitoring of LIB TR by EMSA

Experimental setups for the 3 abuse conditions were integrated into a custom metal gas chamber. A 350-mA·h LFP pouch cell (with safety circuit) was mounted inside on an aluminum platform. All signal cables were shielded and passed through sealed feedthroughs to eliminate radio frequency noise. Mechanical abuse was administered with an microcontroller unit-driven stepper motor and a 2-mm puncture probe; thermal abuse was delivered via a precision hot plate controlling surface temperature; and electrical abuse was applied by connecting the cell to a dc power supply for overvoltage testing.

## Data Availability

The data that support the findings of this study are available from the corresponding author upon reasonable request.
