# Supplementary material for: A Self-Decoupling Multimodal Sensor for Enhanced Early Warning of Lithium-Ion Battery Thermal Runaway
Source: Research (Wash D C). 2026 Feb 24;9:1120. doi: 10.34133/research.1120 (PMC12929815; doi:10.34133/research.1120)
Supplement: Supplementary 1 — Figs. S1 to S28 Tables S1 to S5 Movies S1 to S3 [file research.1120.f1.zip › research_supplementary_materials.docx]

Supplementary Materials for

**A Self-Decoupling Multimodal Sensor for Enhanced Early Warning of Lithium-Ion Battery Thermal Runaway**

Zhenglin Li *et al.*

*Corresponding author. Email: [yanggao@ecust.edu.cn,](mailto:yanggao@ecust.edu.cn,) [liancheng@ecust.edu.cn,](mailto:liancheng@ecust.edu.cn,) [fzxuan@ecust.edu.cn](mailto:fzxuan@ecust.edu.cn)

**This PDF file includes:**

Supplementary Text

Figs. S1 to S22

**Other Supplementary Materials for this manuscript include the following:**

Movies S1 to S3


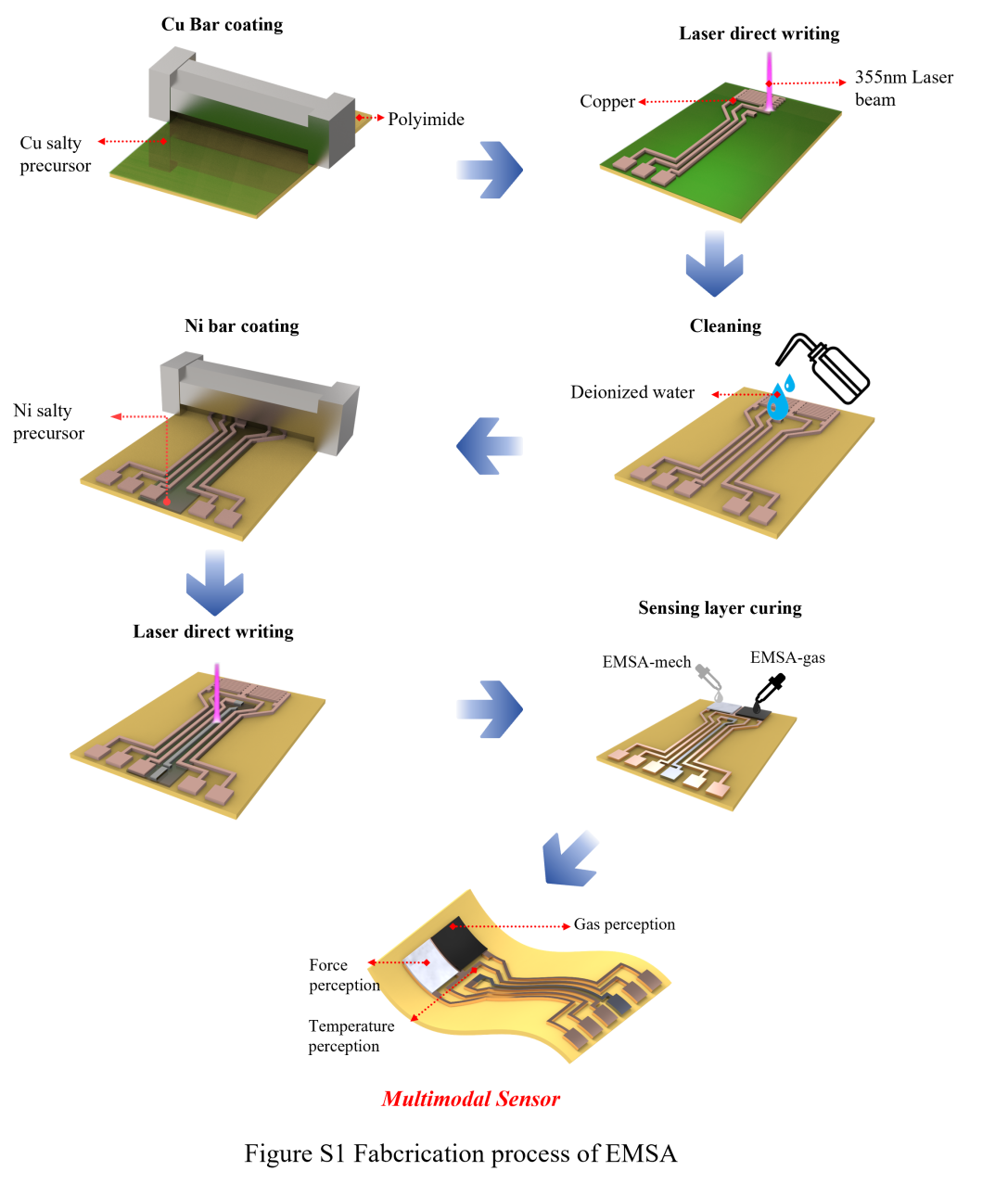


Fig. S1. LDW fabcrication process of EMSA.


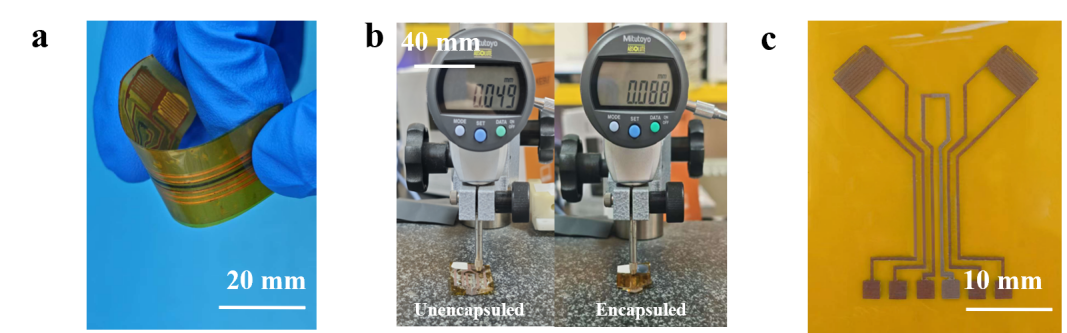


Fig. S2. a, Photo of EMSA ( bending state) b, EMSA thickness with and without encapsulation. c, Customizable configurations of the EMSA in alternative form.


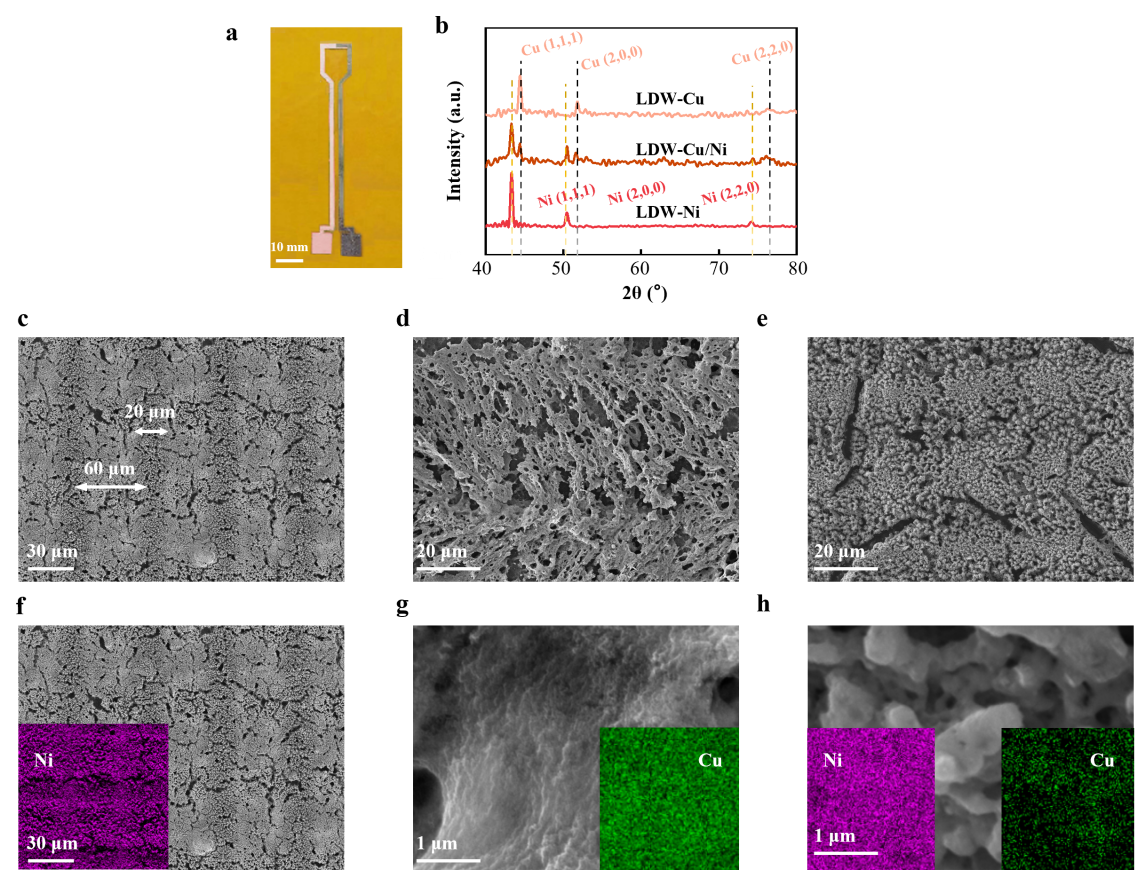


Fig. S3. a, A photograh of LDW EMSA-tem module. b, XRD spectra of the LDW-Cu electrode, LDW-Ni electrode and crosslinking areas. c-e, SEM of the LDW-Cu electrode, LDW-Ni electrode and crosslinking areas. f-h, EDS mapping images of LDW-Cu electrode, LDW-Ni electrode and crosslinking areas.


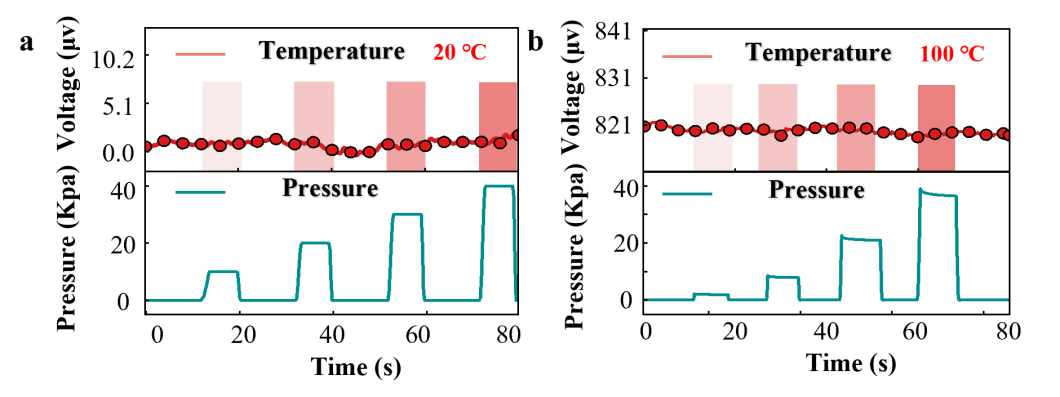


Fig. S4. a, The performance of the EMSA-tem at different pressure at RT. b, The performance of the EMSA-tem at different pressure at 100℃.


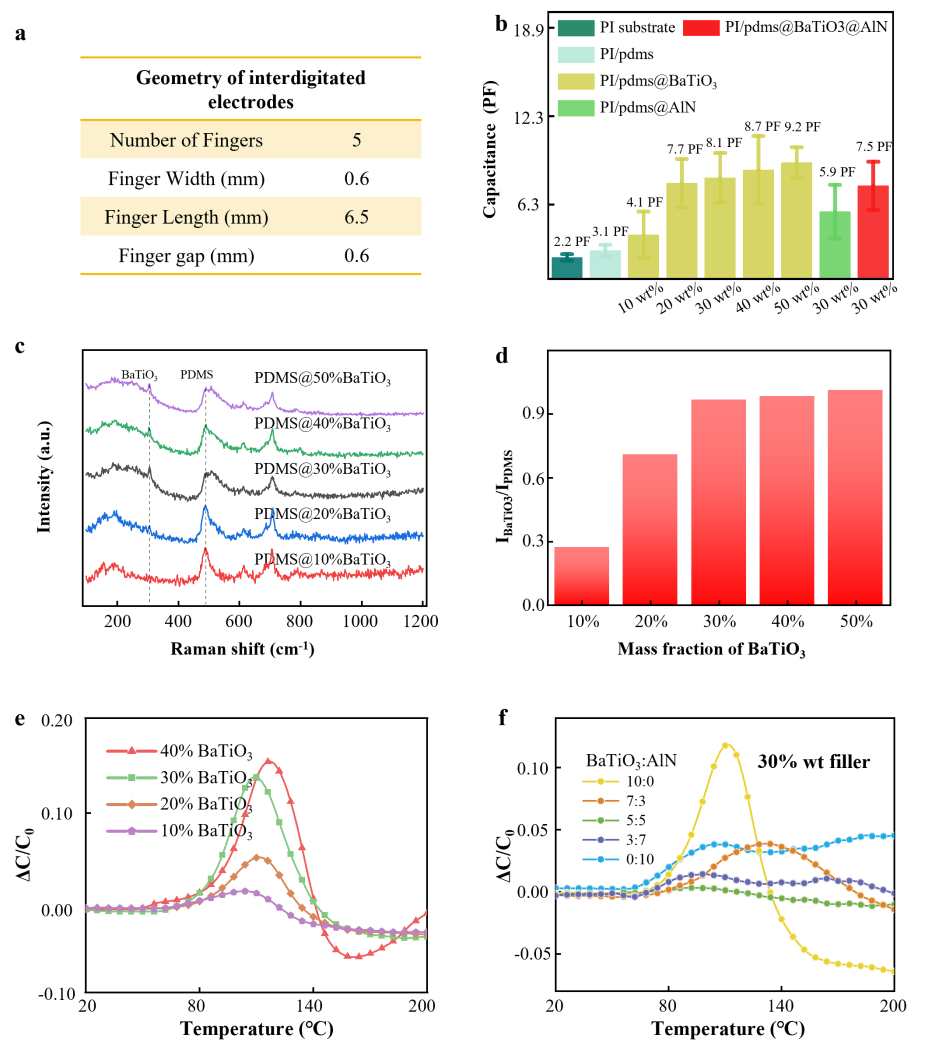


Fig. S5. a, Geometry of the interdigitated electrodes of the EMSA-mech module. b, Initial capacitance of the EMSA-mech module with various dielectric layers. c, Raman spectra of PDMS@BaTiO_3_ composites with different BaTiO_3_ mass fractions. d, Intensity ratio I_BaTiO3_/I_PDMS_ as a function of BaTiO_3_ content e, Temperature-dependent *ΔC/C_0_* response of PDMS@BaTiO_3_ composites with varying BaTiO_3_ loadings. f, Temperature-dependent *ΔC/C_0_* response of the composite at different BaTiO_3_-to-AlN ratios.


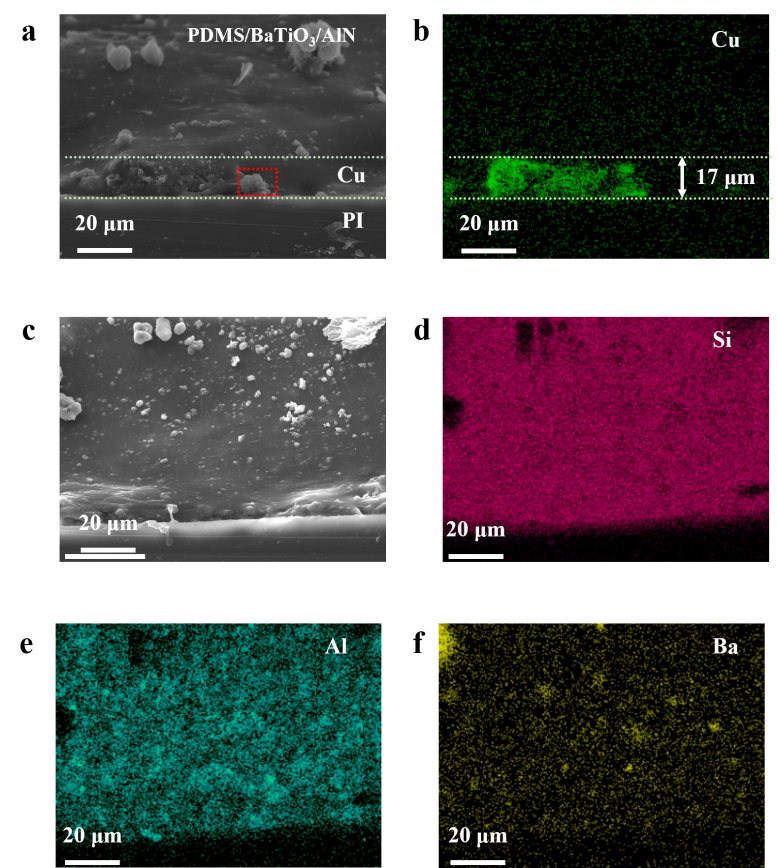


Fig. S6. a, SEM image of the cross-sectional EMSA-mech sensing layer and corresponding EDS image of b, Cu element. c, SEM image of the EMSA-mech sensing layer and its corresponding EDS mapping of d, Si, e, Al , and f, Ba elements.


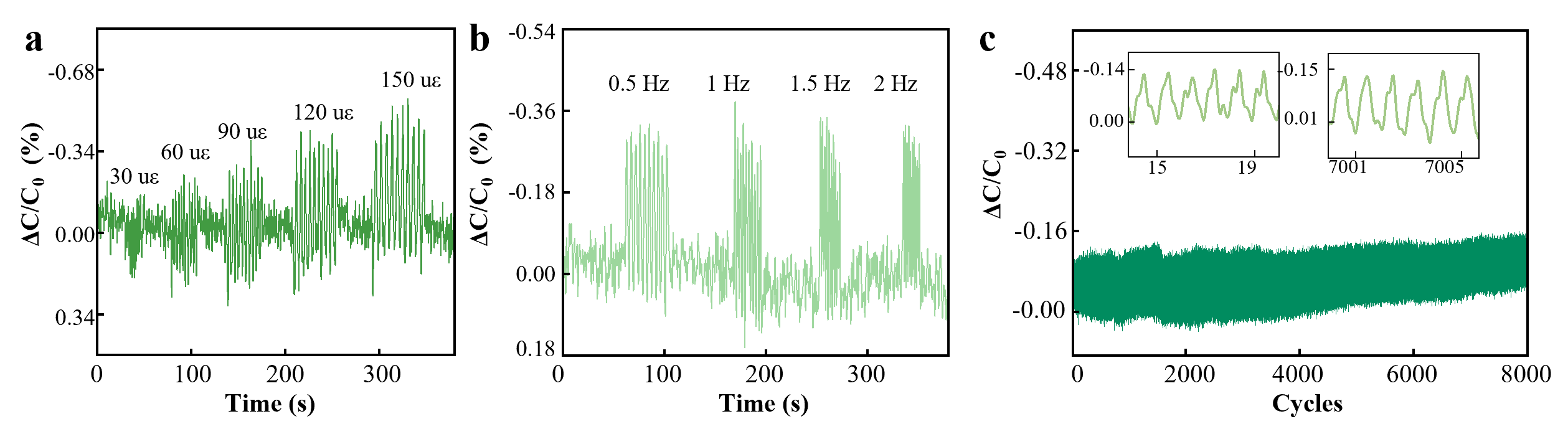


**Figure S7.** a, Performance of the EMSA-mech under small strain cycles; b, Performance of the EMSA-mech under different loading rate at a strain of 90 με. c, Performance of the EMSA-mech under 10,000 strain cycles at a strain of 3000 με.


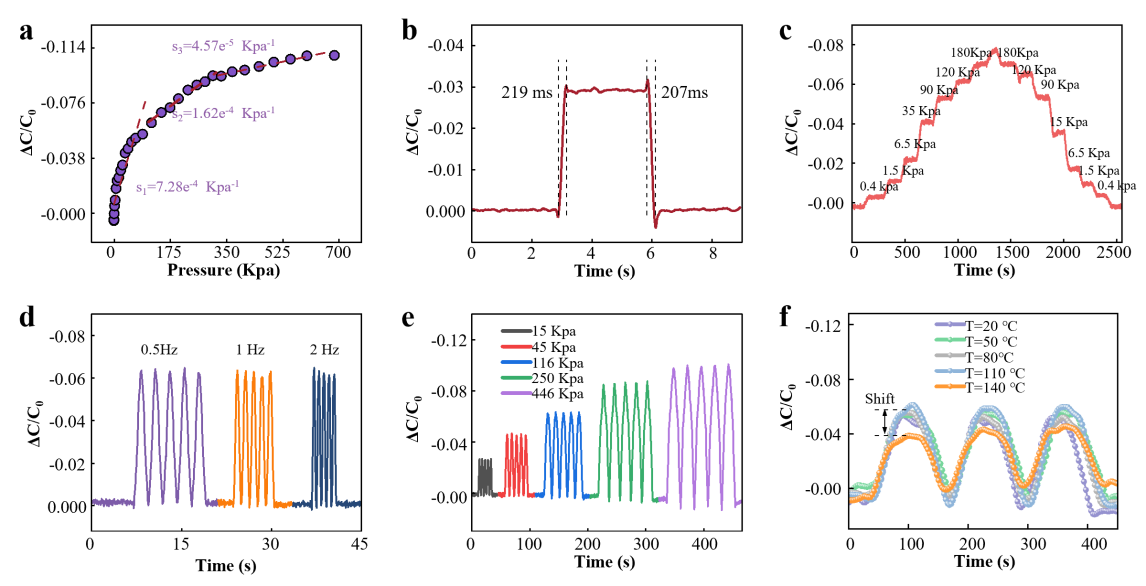


**Figure S8.** a, Sensing performance of the EMSA-mech at different pressures. b, Response and recovery time of the EMSA-mech at 10 kPa. c, *ΔC/C_0_* of the module at different pressures. d, Performance of the EMSA-mech under different loading rate at a pressure of 100 kPa. e. Cycling pressure of the EMSA-mech at different pressures. f, Performance of the EMSA-mech under different temperatures at a pressure of 30 kPa.


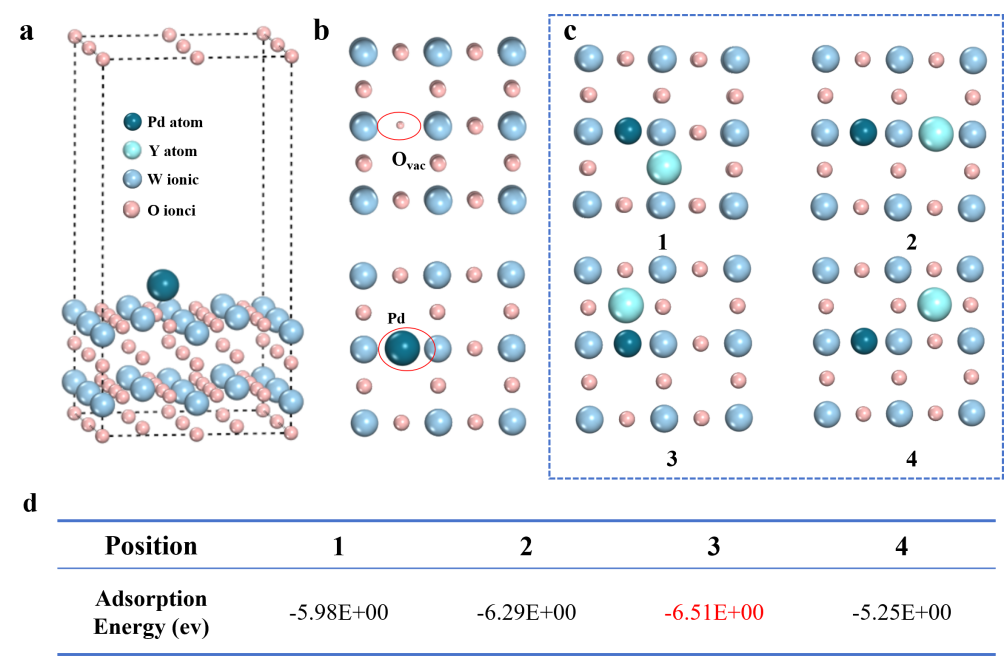


**Figure S9.** a, Constructed PdY@WO_3_ structure. b, Location of Pd atoms occupying oxygen vacancies. c, Four possible configurations of Y atoms. d, Adsorption energies of Y atoms corresponding to the four configurations.


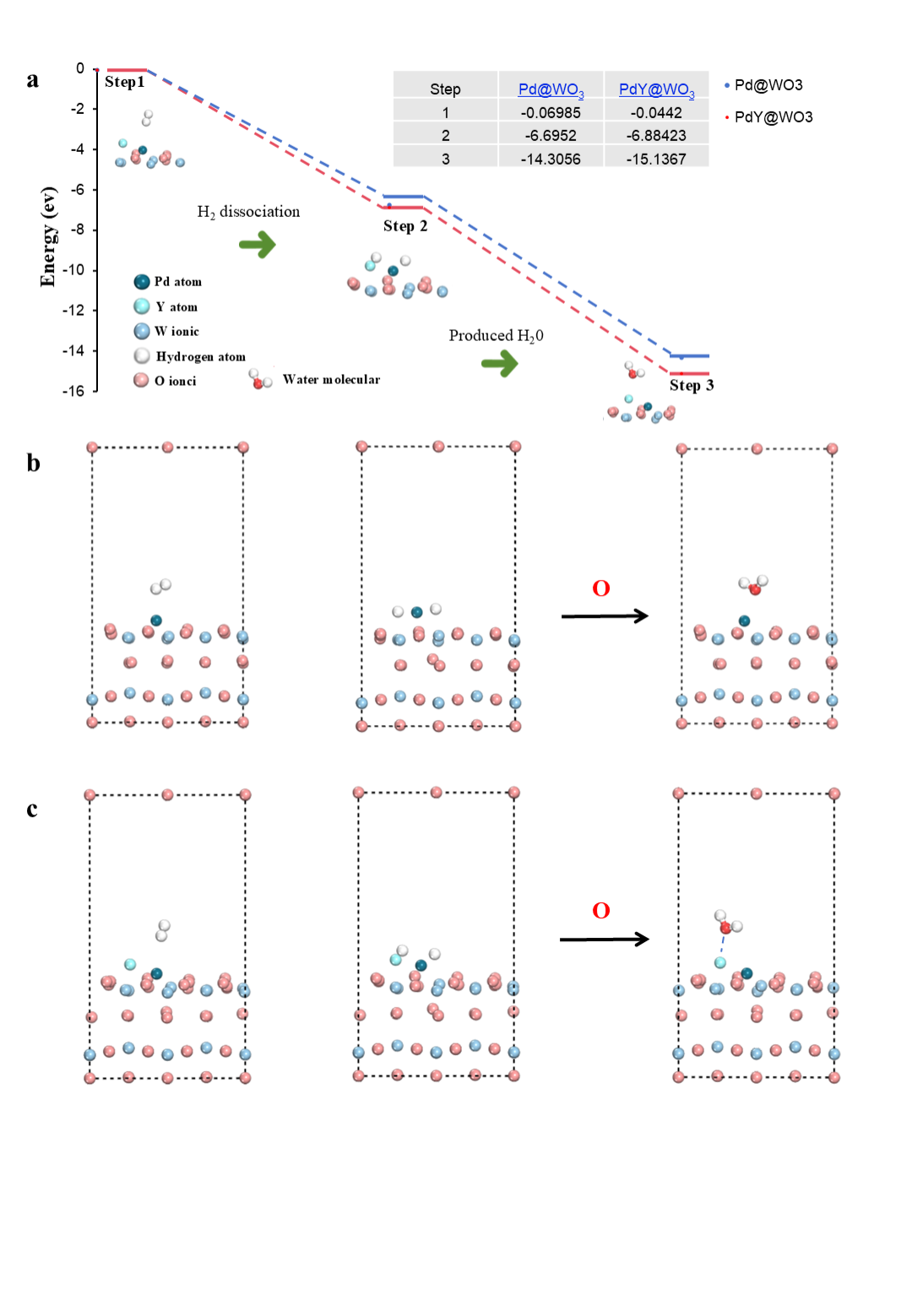


**Figure S10.** a, Calculated hydrogen reaction pathways and associated energy profiles on Pd@WO₃ and PdY@WO₃ surfaces. Schematic illustration of the hydrogen interaction mechanism on the b, Pd@WO₃ sensing layer and c, PdY@WO₃ sensing layer


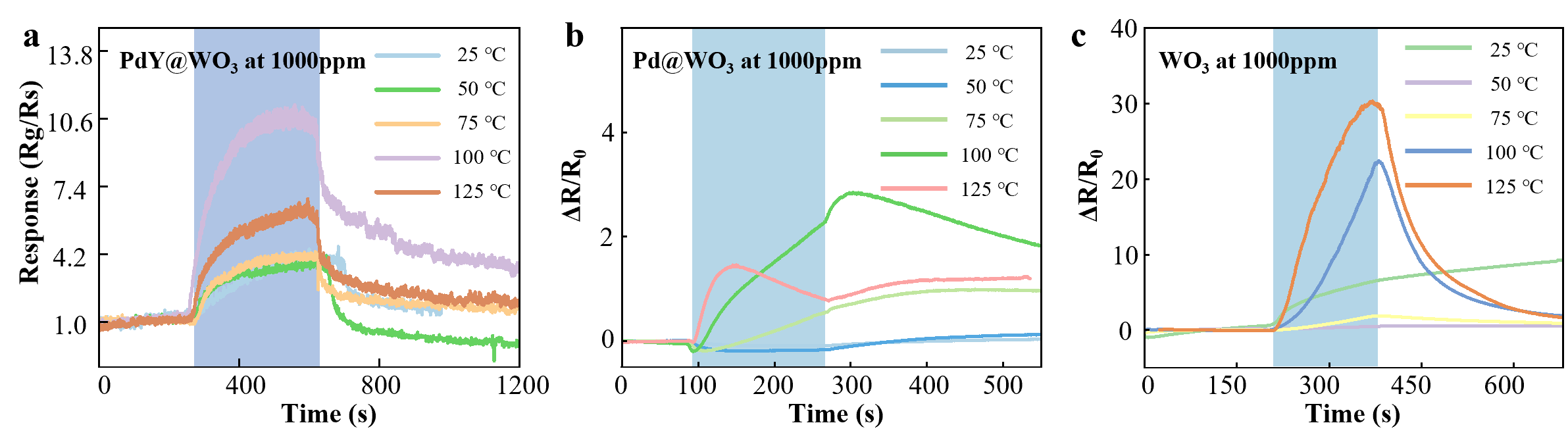


Fig. S11. Hydrogen response performance of the EMSA-gas module with a, PdY@WO_3_, b, Pd@WO_3_, and c, WO_3_ sensing layers at different temperatures.


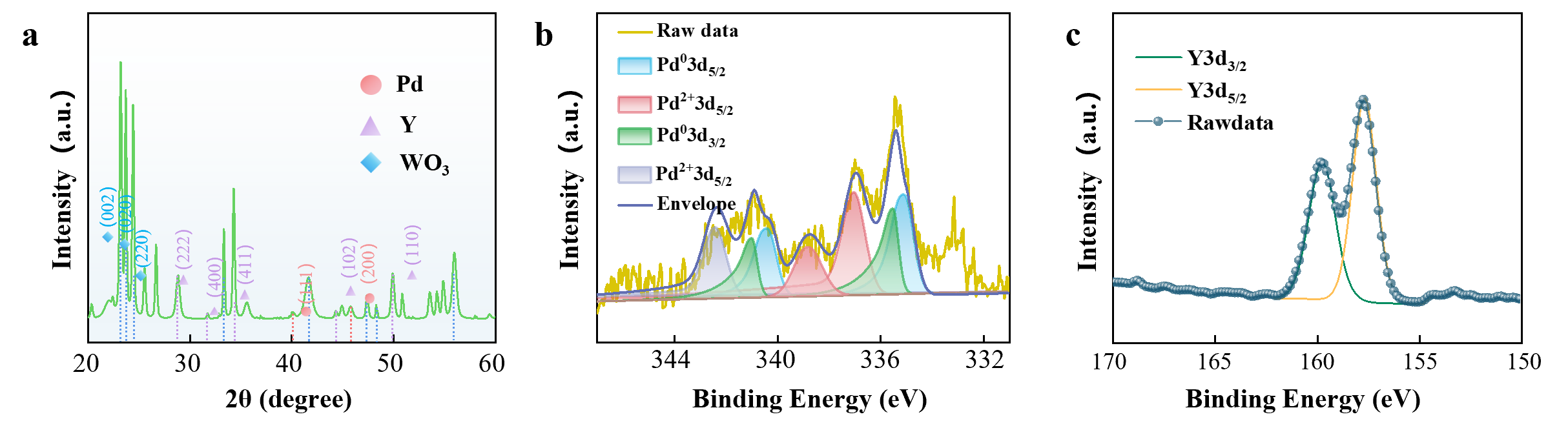


Fig. S12. a, XRD pattern. b and c, XPS spectrum of the EMSA-gas sensing layer.


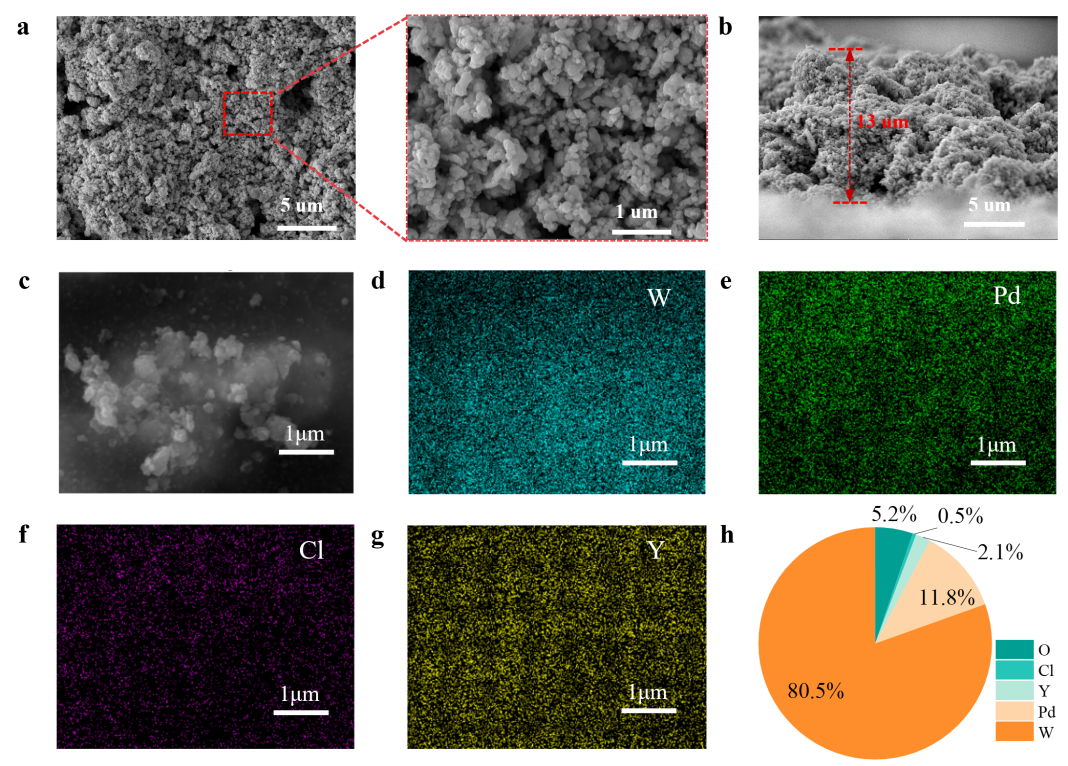


Fig. S13. SEM images of the PdY@WO_3_ gas sensing layer: a, Top-view; b, Cross-sectional view; c, High-magnification SEM image; and corresponding EDS elemental mapping of d, W, e, Pd, f, Cl, and g, Y element. h, Elemental composition analysis of the gas sensing layer.


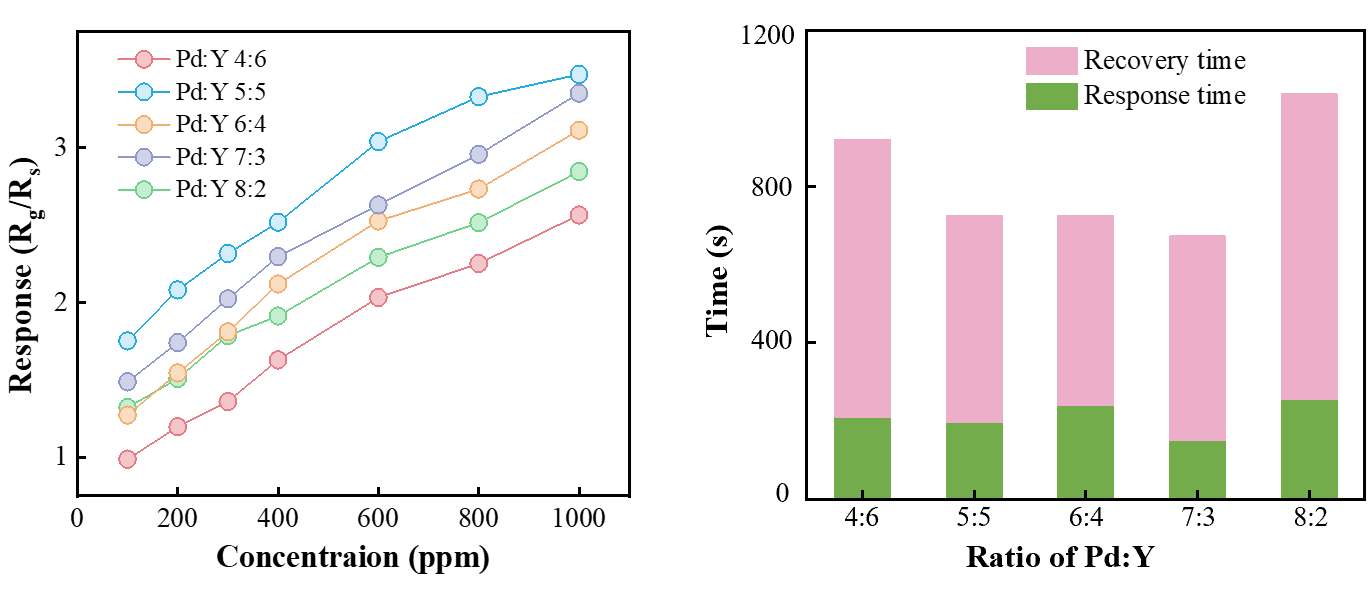


Fig. S14. a, Hydrogen response of the EMSA-gas module with different Pd/Y weight ratios. b, Response and recovery time of the EMSA-gas module with different Pd/Y weight ratios at 1000 ppm hydrogen.


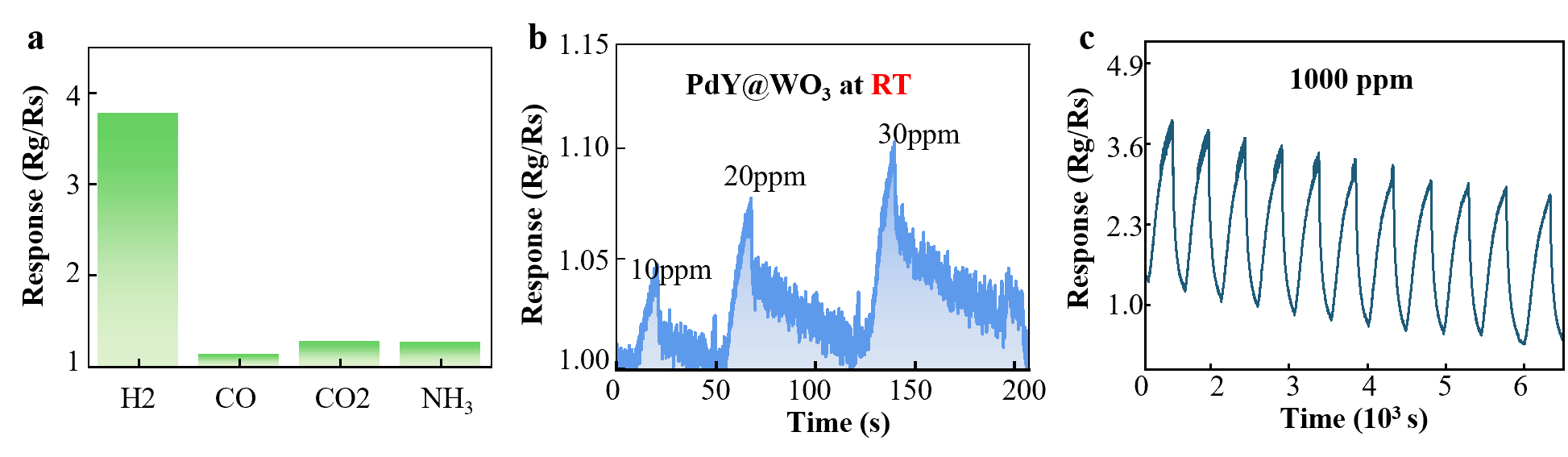


Fig. S15. a, Gas selectivity of the EMSA-gas. b, Response of the EMSA-gas at low hydrogen concentrations. c, Cycling performance of the module at 1000 ppm hydrogen.


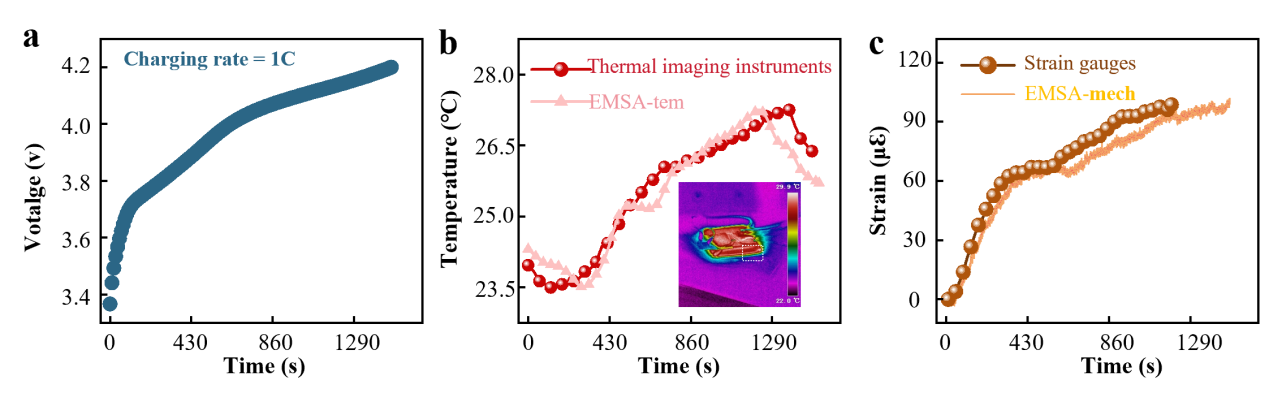


Fig. S16. a, Voltage profile of a lithium-ion battery at a 1C charge rate. b, Surface strain of the LIBs measured by the EMSA and a strain gauge at 1C. c, Surface temperature of the LIBs measured by the EMSA and an infrared thermography at 1C.


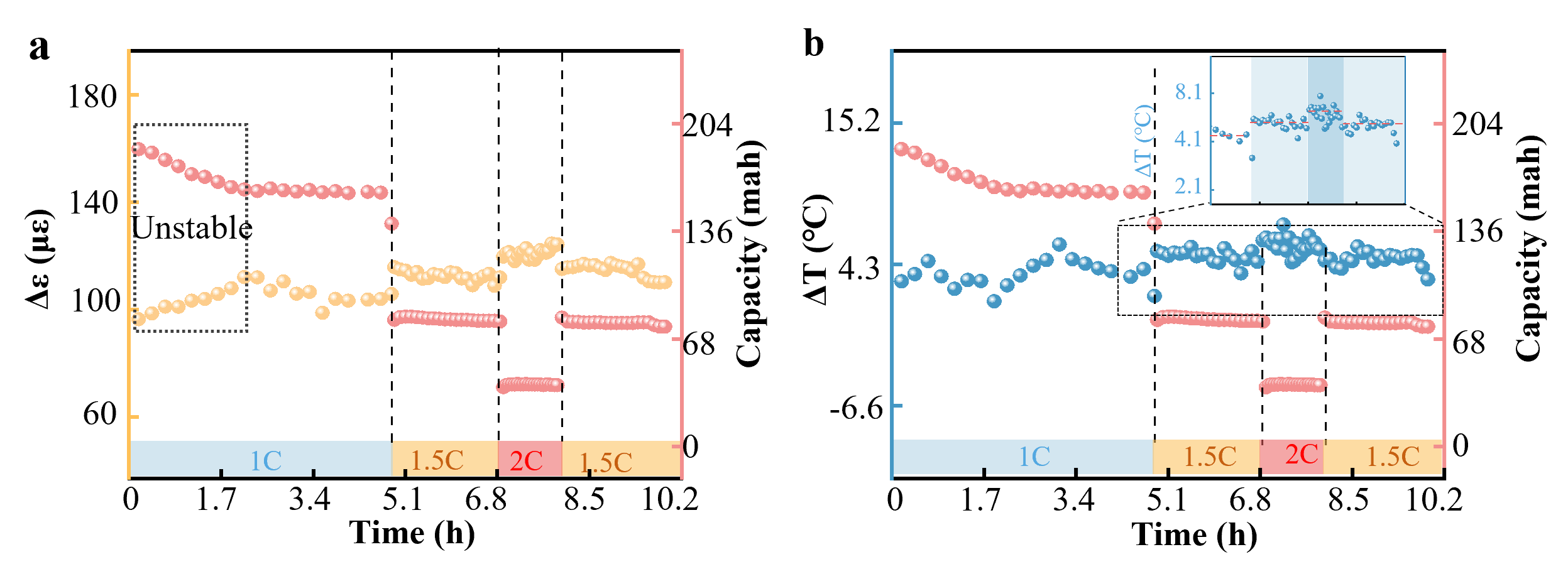


Fig. S17. a, Strain amplitude measured by the EMSA at different charge rates. b, Temperature amplitude measured by the EMSA at different charge rates.


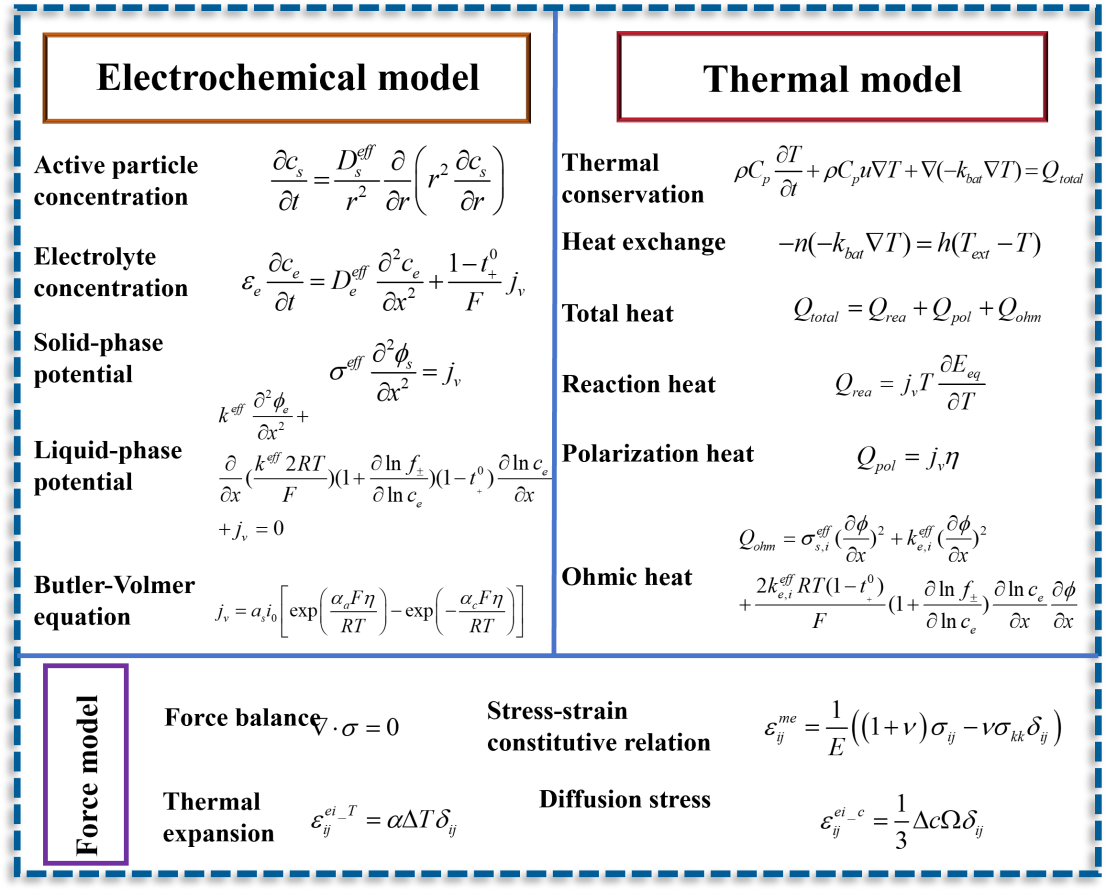


Fig. S18. Electrochemical-thermal-force coupling model of LiFePO₄ pouch cell under normal operation.


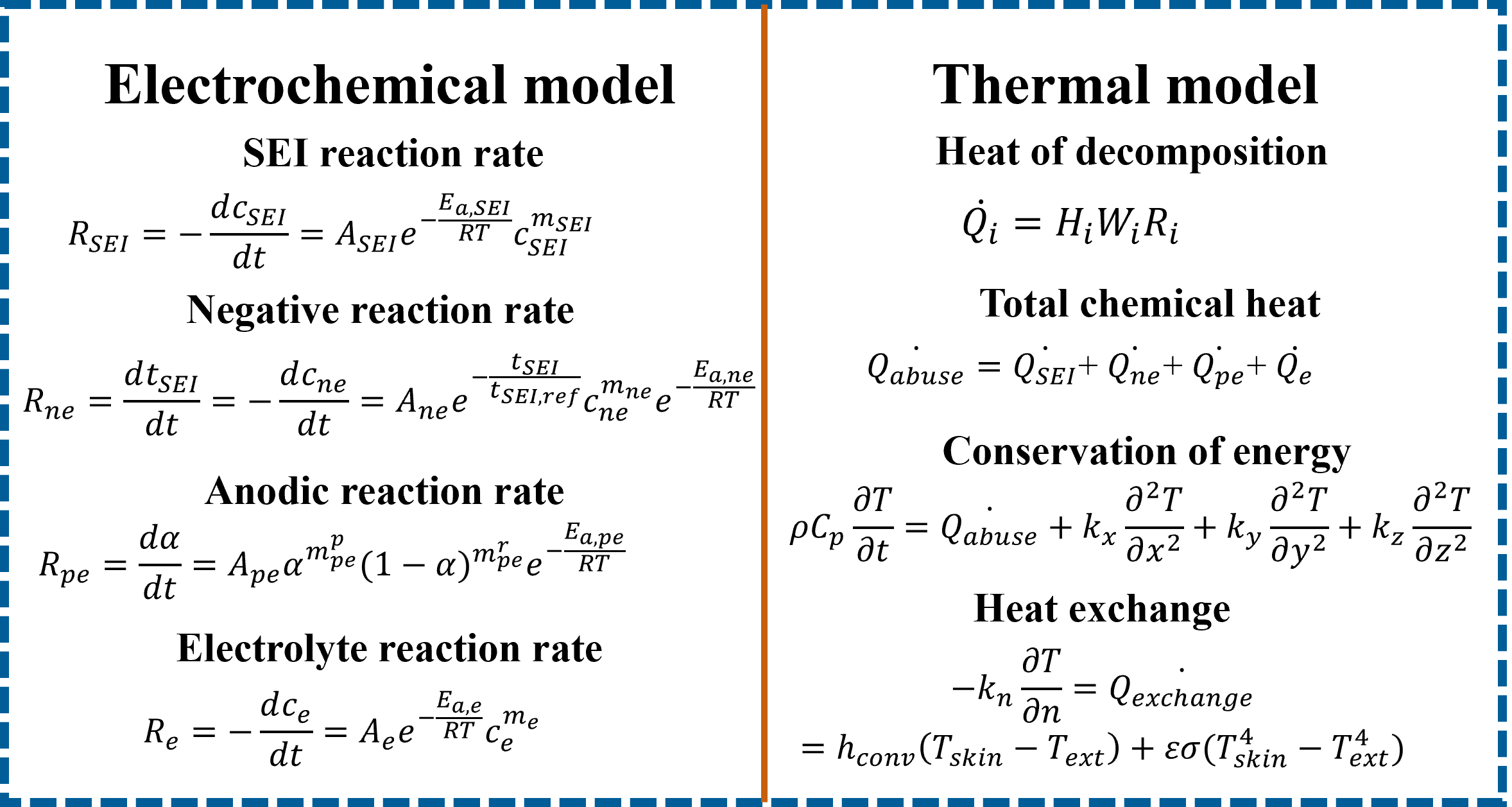


Fig. S19. Electrochemical-thermal coupling model of LiFePO₄ Pouch cell under TR.


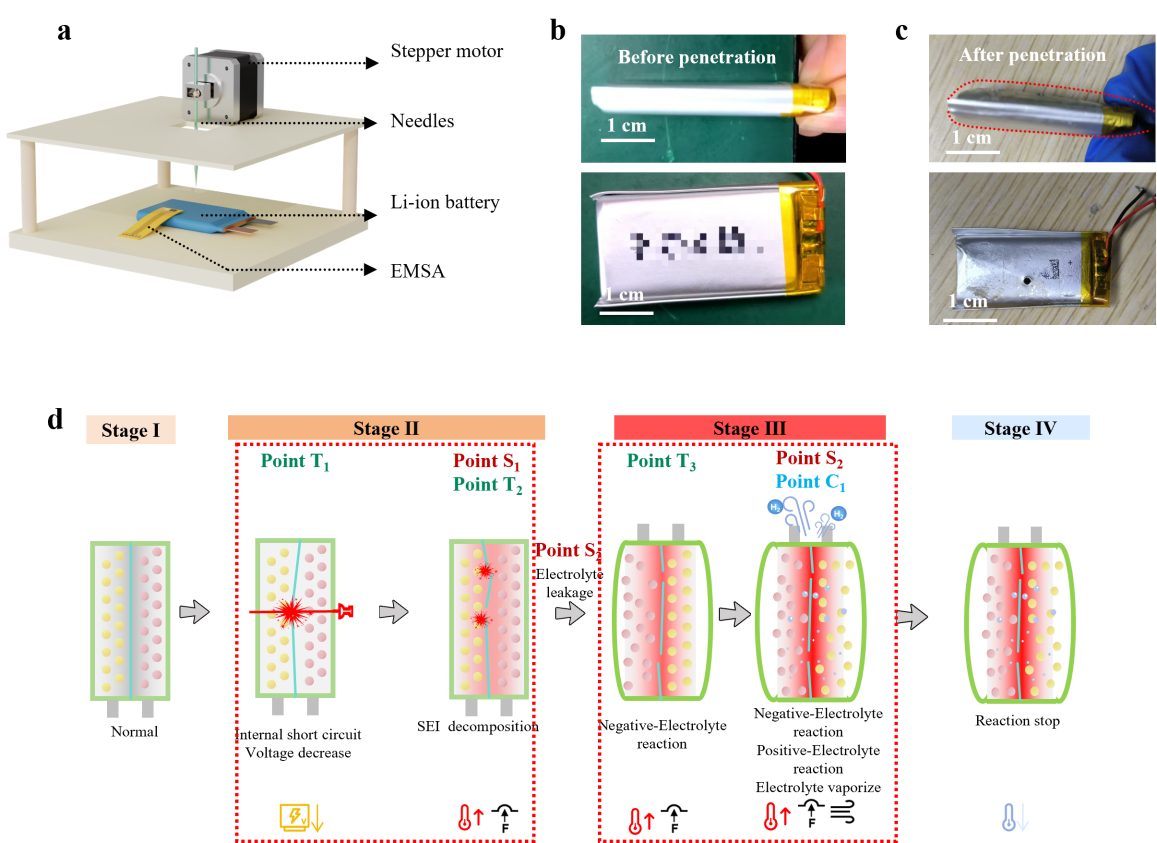


Fig. S20. a, Experimental setup for a puncture-induced TR of lithium-ion batteries. b and c, Photographs of a lithium-ion battery after the puncturing experiment. d, The E-T model-predicted sequence of internal reactions coincides with key multimodal signal transitions, enabling identification of the early warning period for mechanical-induced TR


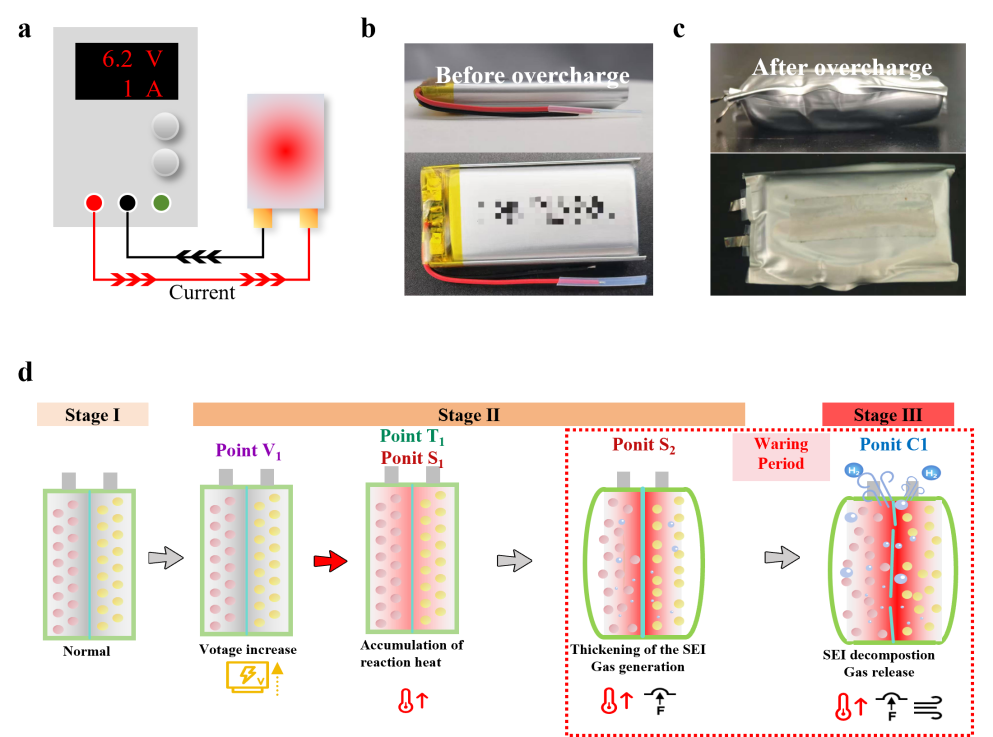


Fig. S21. a, Experimental setup for overcharge TR of LIBs. Comparison of LIB appearance b, before and c, after overcharge. d, The E-T model-predicted sequence of internal reactions coincides with key multimodal signal transitions, enabling identification of the early warning period for electrical-induced TR


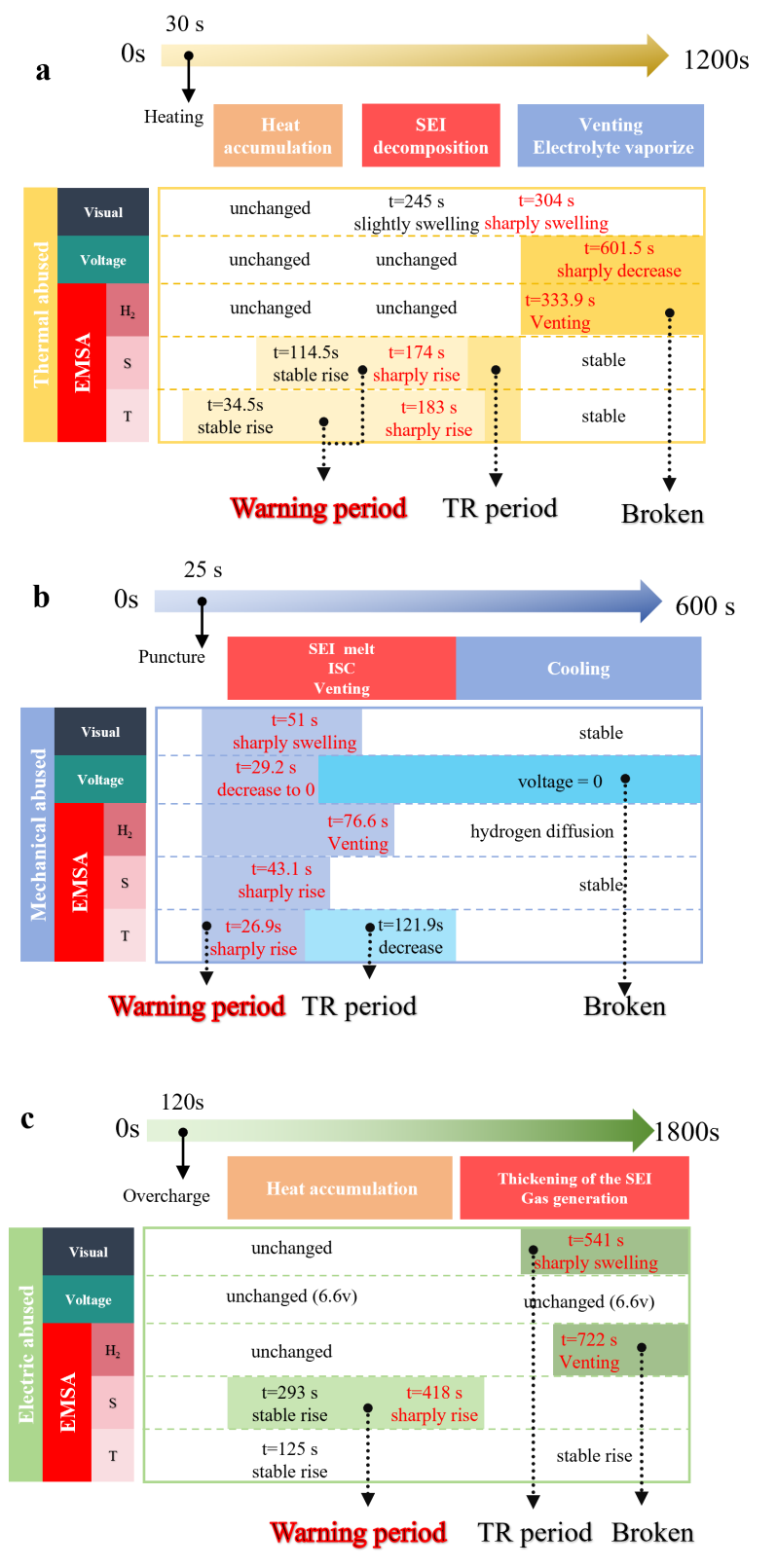


Fig. S22. EMSA-based early-warning windows and reaction stages for lithium-ion battery TR under a, thermal, b, mechanical, and c, electrical abuses.

Movie S1. Multimodal online monitoring of lithium-ion batteries under thermal abuse-induced thermal runaway based on EMSA (20× speed; the thermal runaway early-warning window was intentionally

**Movie S2**. Multimodal online monitoring of lithium-ion batteries under electrical abuse-induced thermal runaway based on EMSA (10× speed; the thermal runaway early-warning window remained active

**Movie S3.** Multimodal online monitoring of lithium-ion batteries under electrical abuse-induced thermal runaway based on EMSA (30× speed; the thermal runaway early-warning window was intentional
