# Supplementary material for: A Self-Decoupling Multimodal Sensor for Enhanced Early Warning of Lithium-Ion Battery Thermal Runaway
Source: Research (Wash D C). 2026 Feb 24;9:1120. doi: 10.34133/research.1120 (PMC12929815; doi:10.34133/research.1120)
Supplement: Supplementary 1 — Figs. S1 to S28 Tables S1 to S5 Movies S1 to S3 [file research.1120.f1.zip › research_supplementary_materials_final.docx]

Supplementary Materials for

**A Self-Decoupling Multimodal Sensor for Enhanced Early Warning of Lithium-Ion Battery Thermal Runaway**

Zhenglin Li *et al.*

*Corresponding author. Email: [yanggao@ecust.edu.cn,](mailto:yanggao@ecust.edu.cn,) [liancheng@ecust.edu.cn,](mailto:liancheng@ecust.edu.cn,) [fzxuan@ecust.edu.cn](mailto:fzxuan@ecust.edu.cn)

**This PDF file includes:**

Supplementary Text

Table S1 to S5

Figs. S1 to S28

**Other Supplementary Materials for this manuscript include the following:**

Movies S1 to S3


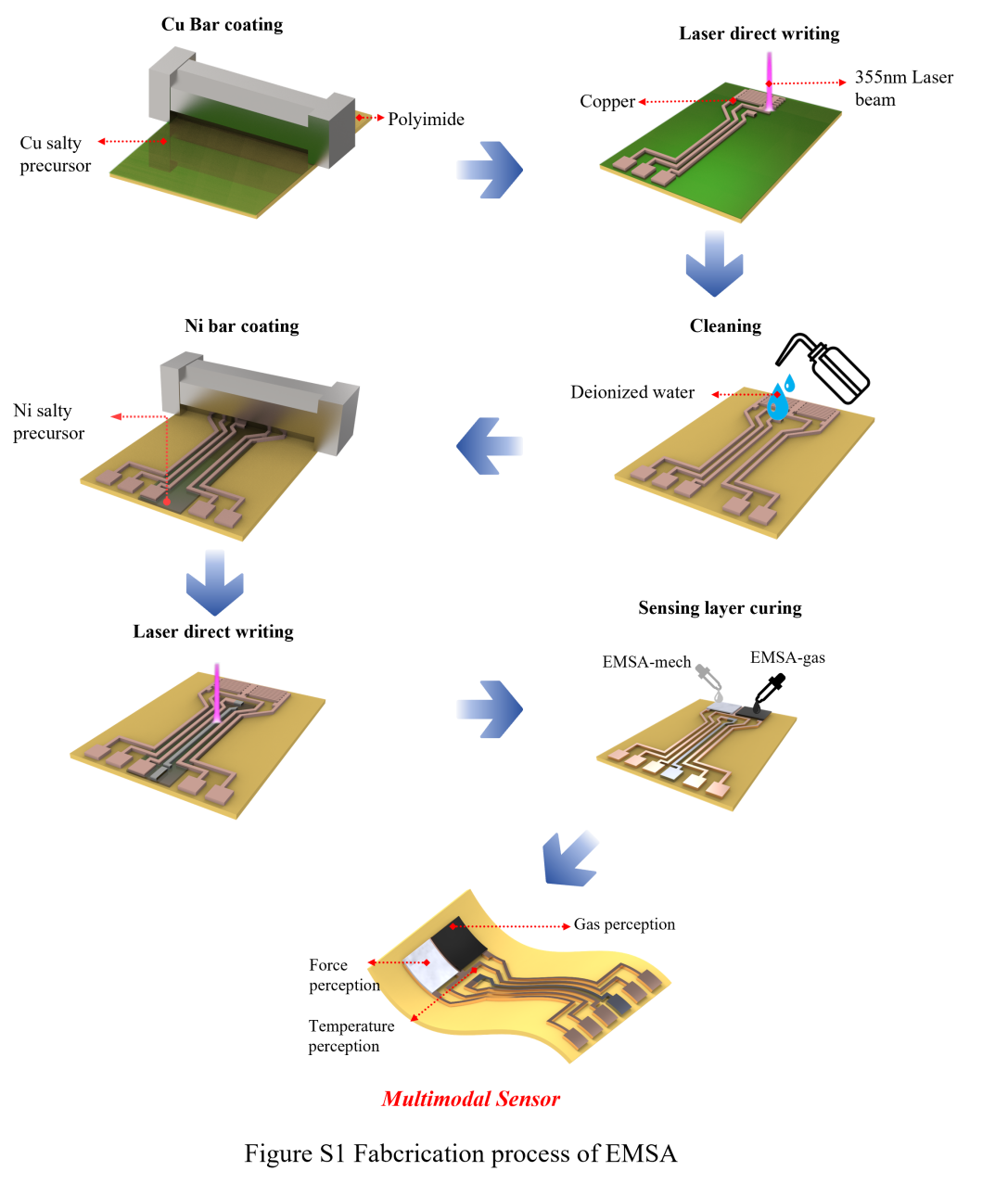


Fig. S1. LDW fabcrication process of EMSA.


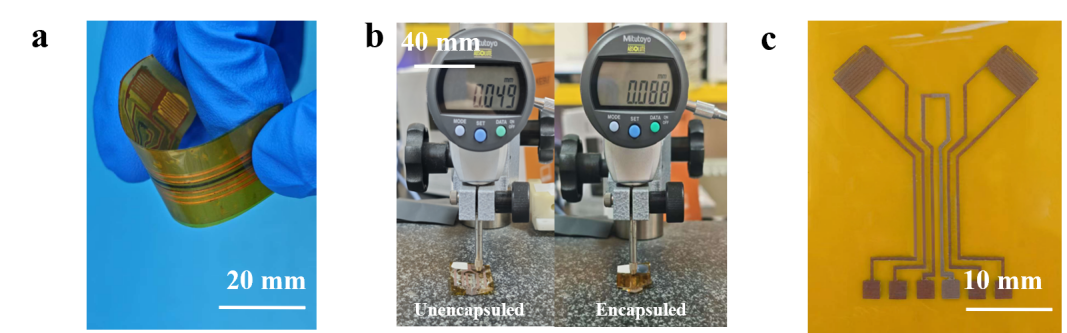


Fig. S2. **a,** Photo of EMSA ( bending state) **b,** EMSA thickness with and without encapsulation. **c,** Customizable configurations of the EMSA in alternative form.

**Table S1.** Manufacturing Costs of EMSA and Comparison with Commercial Sensors

| **Material** | **Material usage** | | **Cost ($)** |
| --- | --- | --- | --- |
| **Substrate (10-piece)** | | | |
| polyimide | 0.03 m^2^ | | 0.14 |
| **EMSA-tem (10-piece)** | | | |
| Hydrated copper nitrate | 2.5 g | | 0.7 |
| ethylene glycol | 2 ml | | 0.021 |
| formic acid | 10 μL | | 0.02 |
| Hydrated nickel nitrate | 3.63 g | | 0.8 |
| n-pentanol | 2.5 ml | | 0.0014 |
| sodium hydroxide | 1.2 g | | 0.021 |
| deionized water | 25 ml | | 0.0002 |
| Polyvinyl pyrrolidone | 0.2 g | |  |
| **EMSA-mech (10-piece)** | | | |
| BaTiO_3_ np | 1.8 g | | 0.18 |
| AlN np | 1.8 g | | 0.5 |
| PDMS | 13.3 g | | 1.27 |
| Aminopropyltriethoxysilane | 0.875 ml | | 0.14 |
| ethanol | 85.5 ml | | 0.38 |
| deionized water | 4.5 ml | | 0.00004 |
| **EMSA-gas (10-piece)** | | | |
| WO_3_ np | 2 g | | 0.13 |
| ethanol | 50 ml | | 0.03 |
| deionized water | 50 ml | | 0.0004 |
| PdCl2 | 0.0196 g | | 3.1 |
| YCl2 | 0.0177 g | | 0.05 |
| pine oil alcohol | 1.8 g | | 0.031 |
| butyl carbitol acetate | 0.9 g | | 0.0045 |
| dibutyl phthalate | 0.3 g | | 0.0016 |
| **Average cost per piece of EMSA: 0.77 $** | | | |
| Commercial thermocouple  (SWT Electric Heating Technology, China, KPS-QB-k*0.2*0.1) | | Cost: 3.9 $ | |
| Commercial strain sensor  (RunesKee, China, BF120-3AA-3CM) | | Cost: 0.87 $ | |
| Commercial gas sensor  (RISYM, China, MQ8) | | Cost: 0.92 $ | |
| Total Cost of commercial sensors: 5.7 $ | | | |


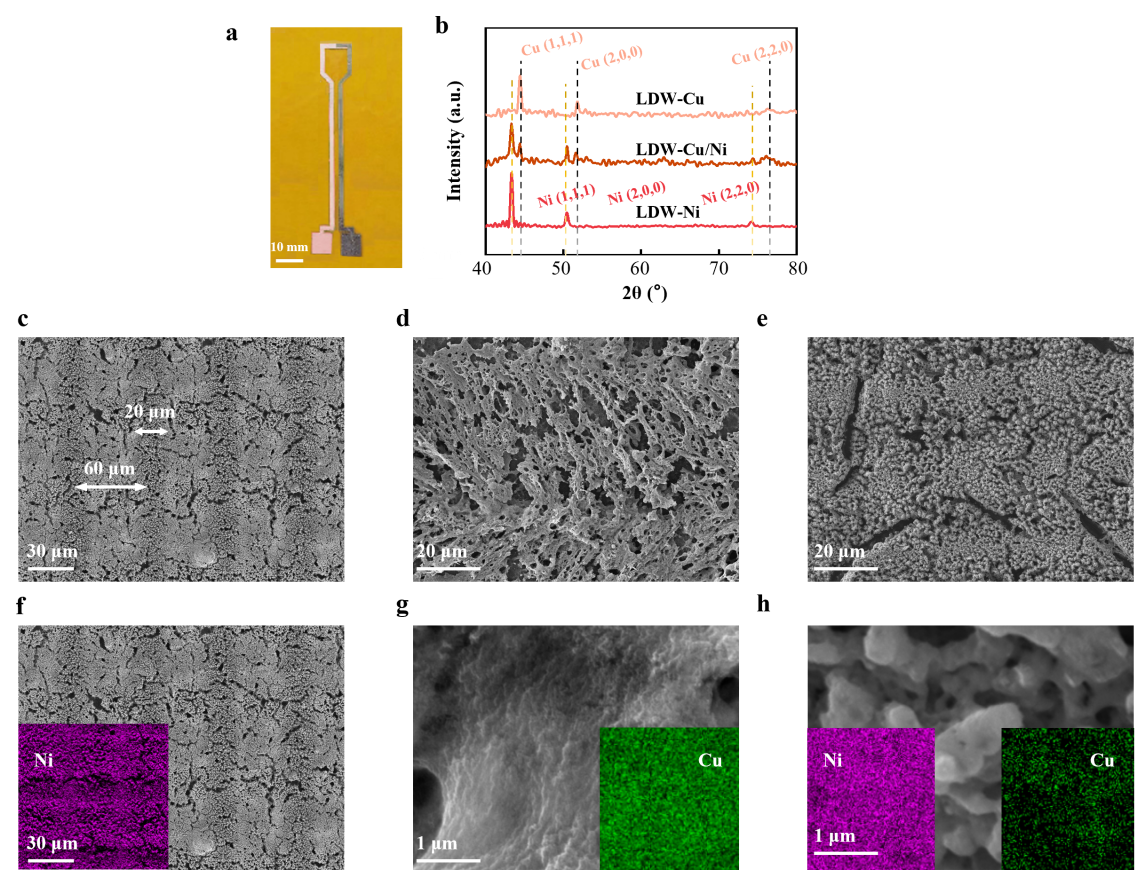


Fig. S3. **a,** A photograh of LDW EMSA-tem module. **b,** XRD spectra of the LDW-Cu electrode, LDW-Ni electrode and crosslinking areas. **c-e,** SEM of the LDW-Cu electrode, LDW-Ni electrode and crosslinking areas. **f-h,** EDS mapping images of LDW-Cu electrode, LDW-Ni electrode and crosslinking areas.

**Characterization and Analysis of EMSA-tem Electrodes**

Fig. S3a confirms the formation of robust thermocouple junctions between the LDW-synthesized Cu and Ni. XRD (Fig. S3b) exhibits Ni peaks at 44.4°(111), 51.7°(200), and 76.2°(220) (FCC Ni^[1]^) and Cu peaks at 43.3°(111), 50.4°(200), and 74.1°(220) ( FCC Cu^[2]^). In the Cu-Ni crosslinking region, both Cu and Ni peaks are present, with diminished Cu intensity due to Ni overlayer coverage. Four-probe measurements reveal that the resistivities of the LDW-fabricated Cu, Ni, and their crosslinking region are approximately 125 μΩ·cm, 670 μΩ·cm, and 198 μΩ·cm, respectively. SEM micrographs of the LDW-synthesized Ni, Cu, and crosslinking regions (Fig. S3c–e) exhibit porous surface morphologies attributed to gas evolution during laser patterning, which limits conductivity compared to bulk metal. ^[3]^ High-magnification SEM and EDS mappings (Fig. S3e and h) confirm that the Ni electrode uniformly overlies the Cu electrode, forming continuous conductive pathways.


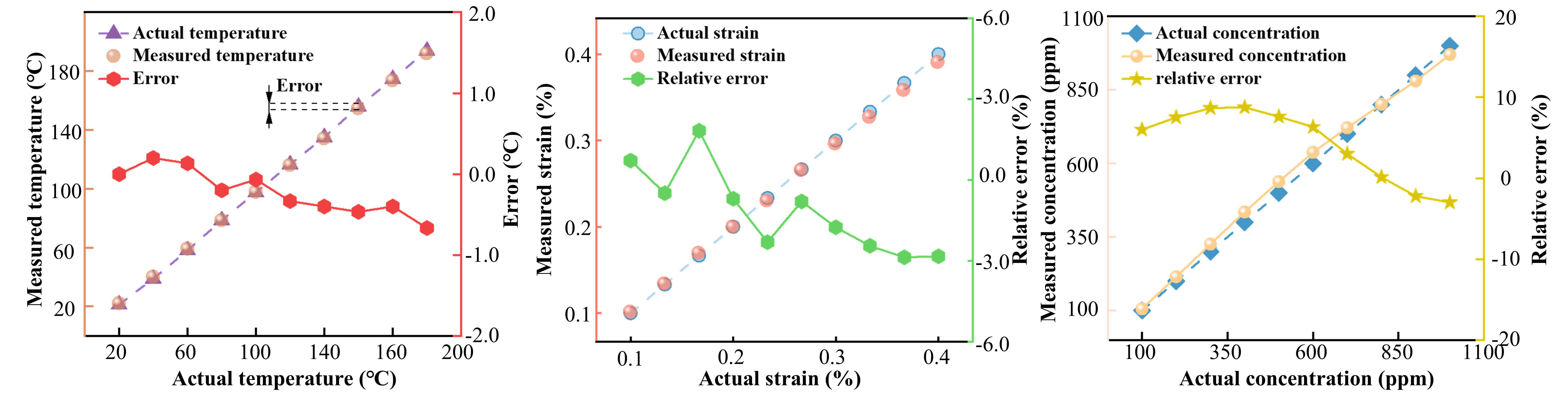


**Fig. S4.** Temperature response accuracy test of EMSA-tem.


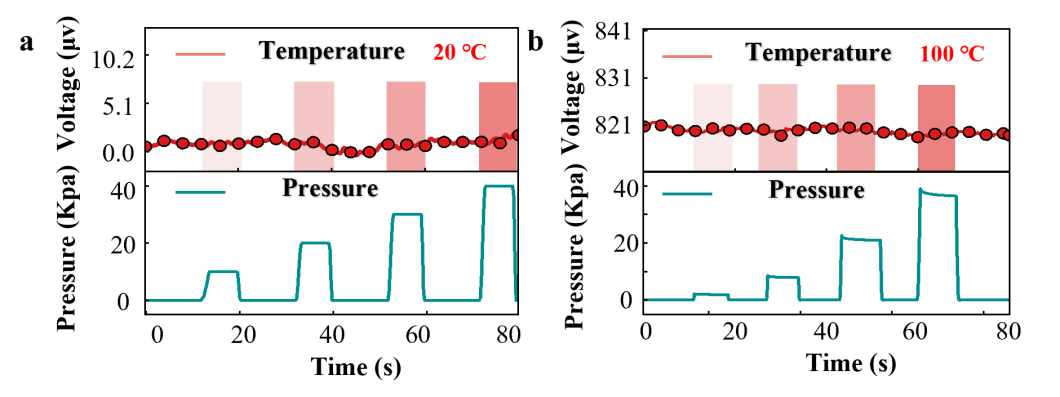


Fig. S5. **a,** The performance of the EMSA-tem at different pressure at RT. **b,** The performance of the EMSA-tem at different pressure at 100℃.


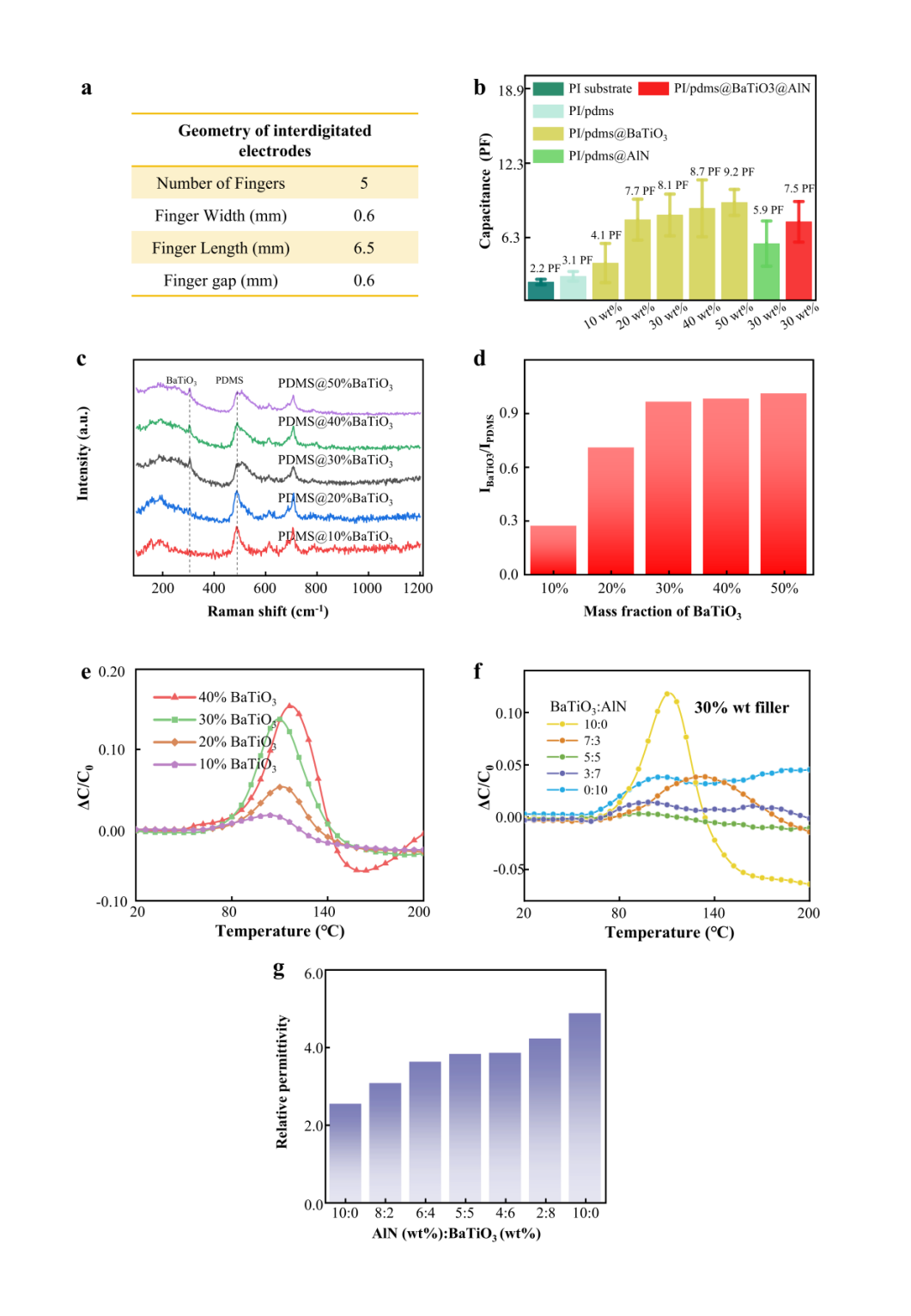


Fig. S6. **a,** Geometry of the interdigitated electrodes of the EMSA-mech module. **b,** Initial capacitance of the EMSA-mech module with various dielectric layers. **c,** Raman spectra of PDMS@BaTiO_3_ composites with different BaTiO_3_ mass fractions. **d,** Intensity ratio I_BaTiO3_/I_PDMS_ as a function of BaTiO_3_ content **e,** Temperature-dependent *ΔC/C_0_* response of PDMS@BaTiO_3_ composites with varying BaTiO_3_ loadings. **f,** Temperature-dependent *ΔC/C_0_* response of the composite at different BaTiO_3_-to-AlN ratios. **g.**Relative permittivity of the EMSA-mech composite as a function of the mass ratio between AlN and BaTiO_3_

_
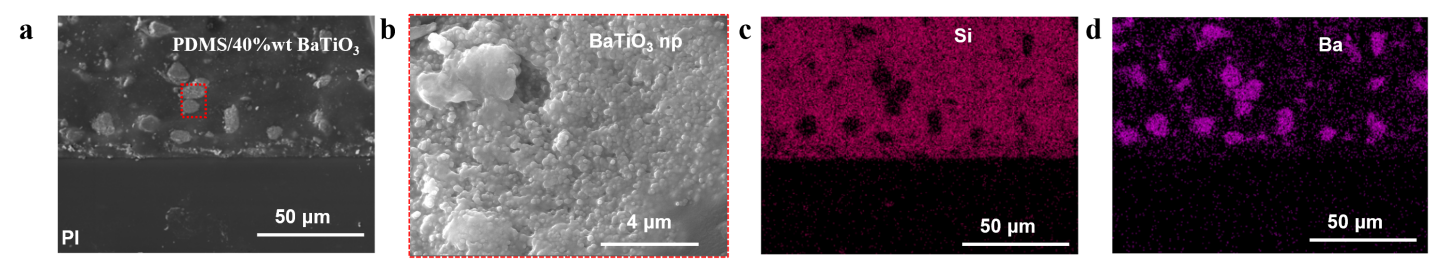
_

**Fig. S7** Characterization of EMSA-mech filled with 40 wt% BaTiO_3_: **a,** SEM image of the EMSA-mech. **b,** high-magnification SEM image showing the agglomerated BaTiO_3_ nanoparticles. and corresponding EDS elemental mappings of **c,** Si and **d,** Ba elements.


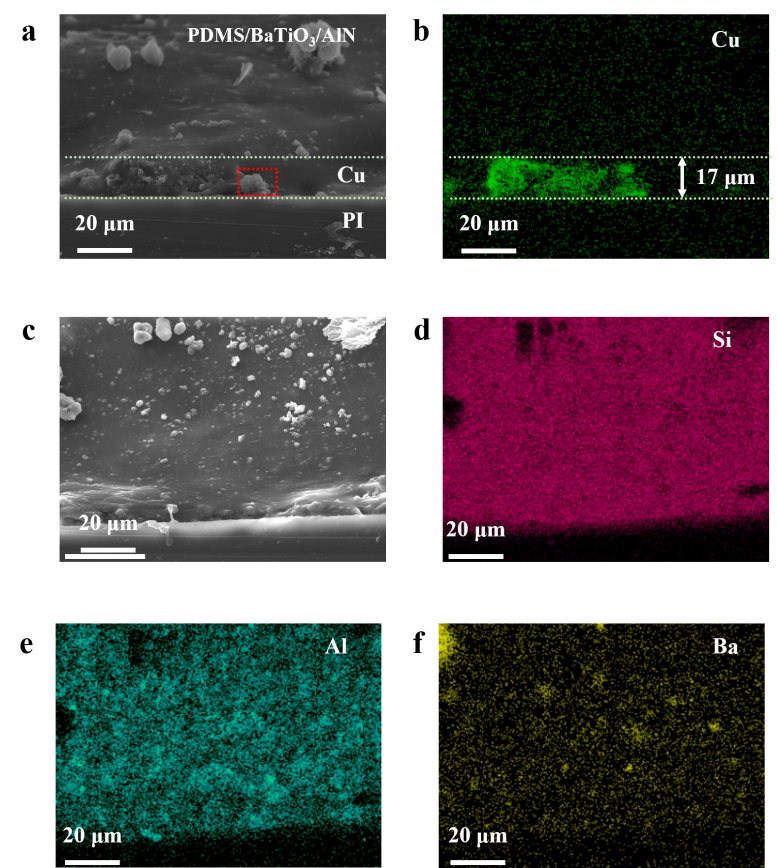


Fig. S8. a, SEM image of the cross-sectional EMSA-mech sensing layer and corresponding EDS image of b, Cu element. c, SEM image of the EMSA-mech sensing layer and its corresponding EDS mapping of d, Si, e, Al , and f, Ba elements.

**SEM characterization and analysis of EMSA-mech**

Fig. S8 displays cross-sectional SEM images and EDS mappings of the EMSA-mech module. In Fig. S8b and c, the LDW-fabricated Cu electrode (17 μm thickness) is fully embedded in the sensing layer. Figures S8e-h confirm that BaTiO3 and AlN nanoparticles are uniformly dispersed throughout the PDMS matrix.


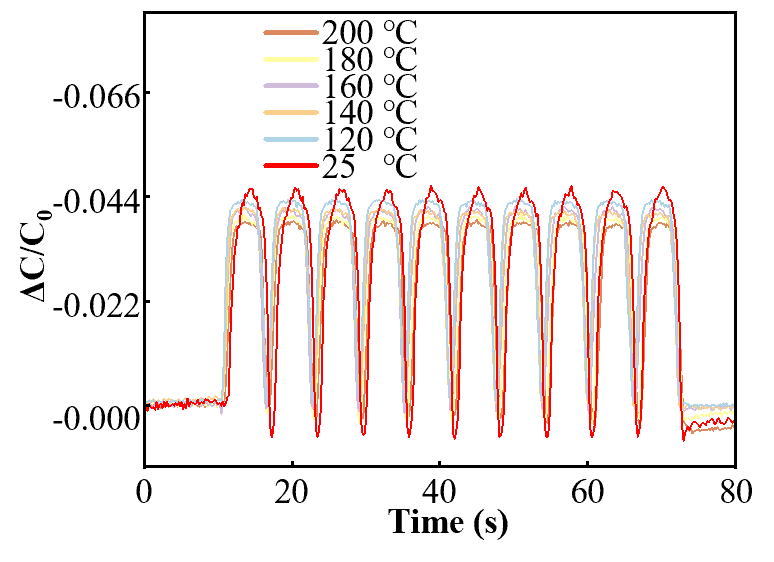


**Fig. S9.** *ΔC/C_0_* response of EMSA under 1000 µε at various temperatures (120–200 °C).


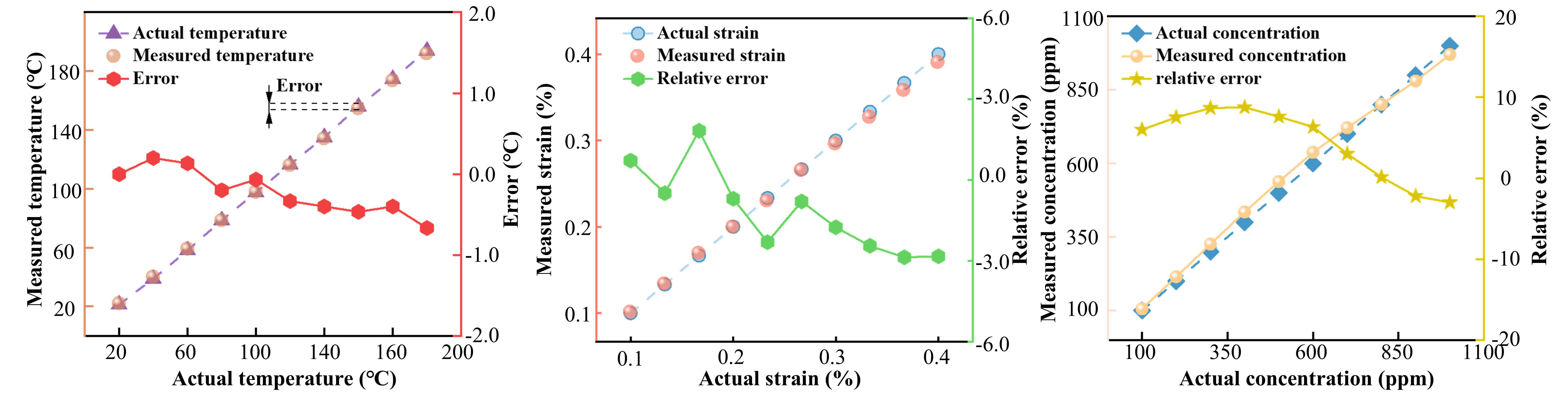


**Fig. S10.** Strain response accuracy test of EMSA-mech.


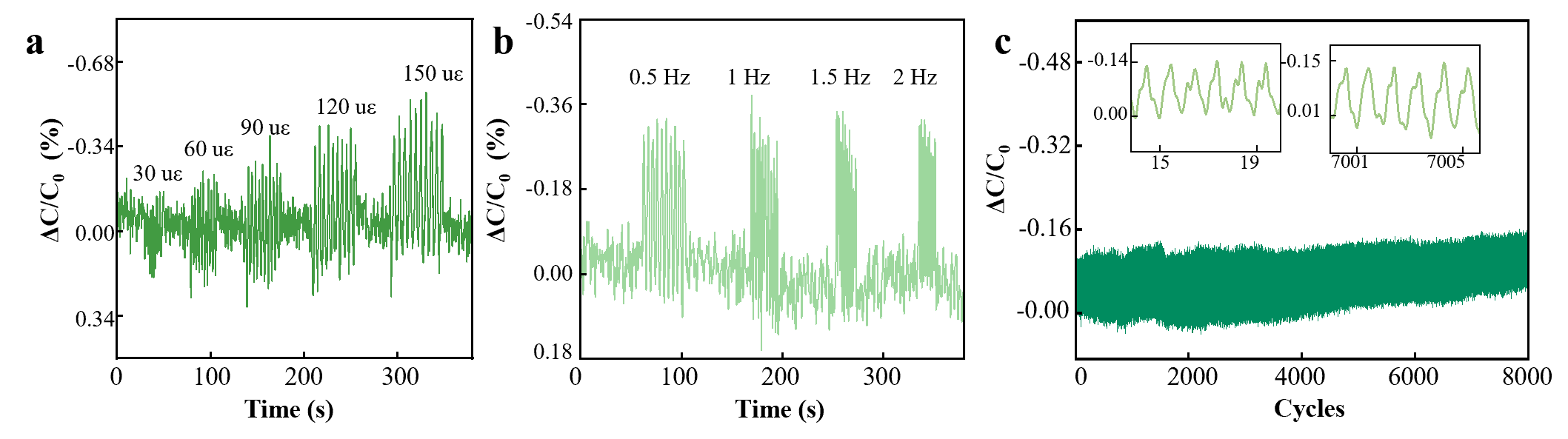


**Figure S11.** a**,** Performance of the EMSA-mech under small strain cycles; **b,** Performance of the EMSA-mech under different loading rate at a strain of 90 με. **c,** Performance of the EMSA-mech under 10,000 strain cycles at a strain of 3000 με.


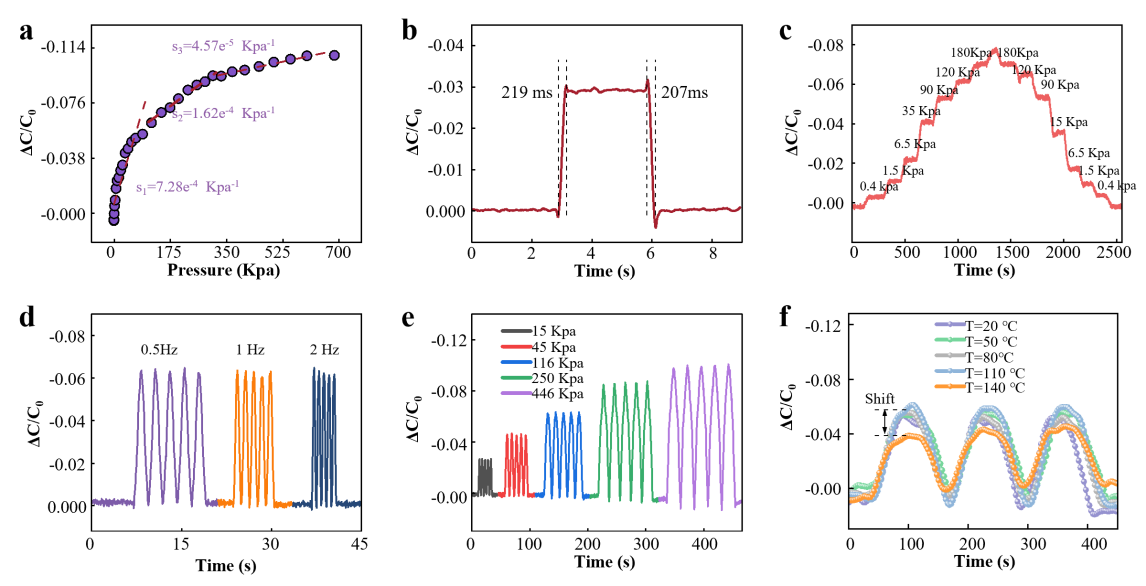


**Figure S12.** **a,** Sensing performance of the EMSA-mech at different pressures. **b,** Response and recovery time of the EMSA-mech at 10 kPa. **c,** *ΔC/C_0_* of the module at different pressures. **d,** Performance of the EMSA-mech under different loading rate at a pressure of 100 kPa. **e.** Cycling pressure of the EMSA-mech at different pressures. **f,** Performance of the EMSA-mech under different temperatures at a pressure of 30 kPa.

**Performance study of EMSA as a pressure sensor**

The EMSA-mech also could function as a pressure sensor, extending its application scope. Fig. S12 summarizes the device’s pressure-sensing performance. As shwon in Fig. S12a, in the 0-54 kPa range, the module has a highest pressure sensitivity of -7.28·10-4 kpa-1. The EMSA-mech has a response and recovery time of 219 and 207 ms, respectively, at 20 kpa (Fig. S12b). Fig. S8c demonstrates the device’s excellent reversibility and consistency under gradually increasing static pressure. The frequency variation does not significantly affect the pressure sensitivity of the device (Fig. S12d). Fig. S12e shows that the device exhibits stable and repeatable responses under cyclic pressure. Fig. S12f illustrates the temperature cross-talk effect on the device’s pressure response. Similar to the strain response, temperature has minimal impact on pressure sensing within the 20-110 ℃ range.


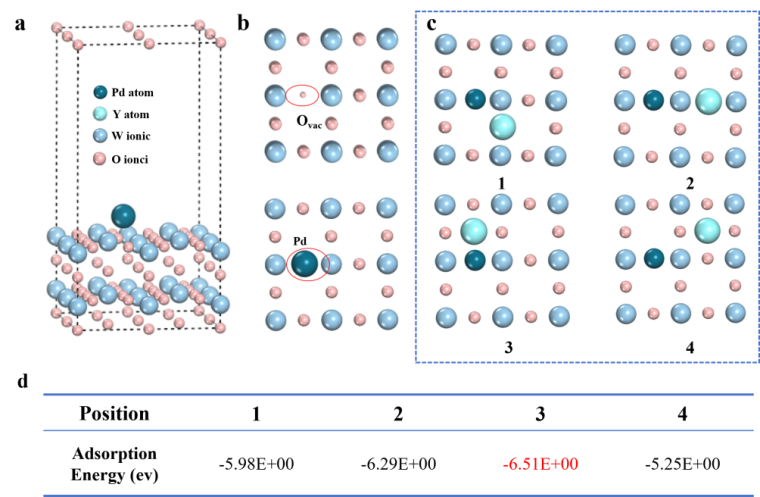


**Figure S13. a,** Constructed PdY@WO_3_ structure. **b,** Location of Pd atoms occupying oxygen vacancies. **c,** Four possible configurations of Y atoms. **d,** Adsorption energies of Y atoms corresponding to the four configurations.


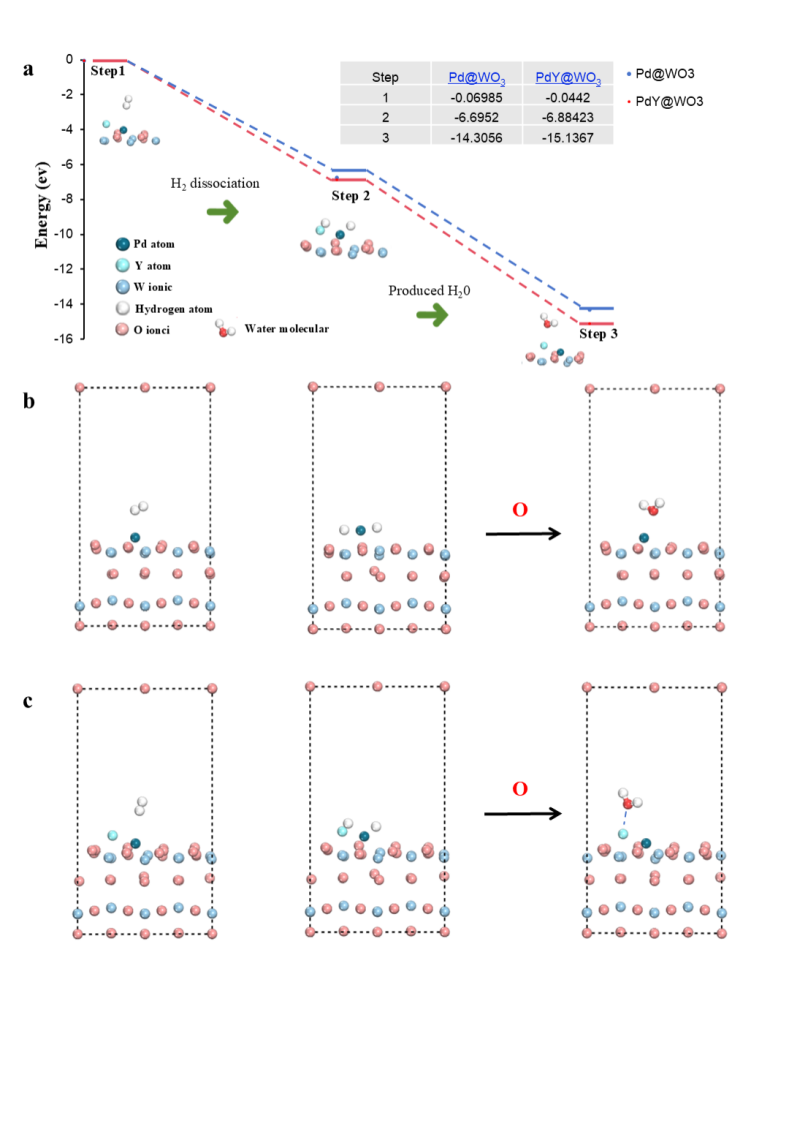


**Figure S14.** **a,** Calculated hydrogen reaction pathways and associated energy profiles on Pd@WO₃ and PdY@WO₃ surfaces. Schematic illustration of the hydrogen interaction mechanism on the **b,** Pd@WO₃ sensing layer and **c,** PdY@WO₃ sensing layer

**Analyze the superiority of PdY@ WO3 as a hydrogen-sensing material using DFT**

Figs. S12–13 demonstrate the calculated H_2_ adsorption behavior of PdY@WO_3_ by first-principles DFTs. Firstly, the computational model was constructed using the WO_3_ (100) facet. Based on literature reports that Pd atoms preferentially occupy surface oxygen vacancies^[4]^, a four-layer W-terminated slab was generated, and a bridging oxygen atom was removed to create an adsorption site (Fig. S12a). A Pd atom was then introduced into this vacancy (Fig. S12b), followed by systematic variation of the Y atom position to determine the lowest-energy configuration. Four candidate adsorption sites were identified (Fig. S9c), and their adsorption energies were plotted in Fig. S9d. Configuration 3 exhibited the lowest adsorption energy, indicating the most favorable site for Y incorporation. This configuration was therefore used to build the final PdY@WO3 model. Fig. S10 depicts the H₂ reaction pathway on PdY@WO_3_. Upon H2 exposure, H2 molecules dissociate on Pd and subsequently react with adsorbed oxygen to form H2O. Notably, PdY@WO₃’s energy profile lies below that of Pd@WO_3_, indicating a more favorable reaction. Pd catalyzes H₂ dissociation, while Y captures O from air and reacts with H atoms.


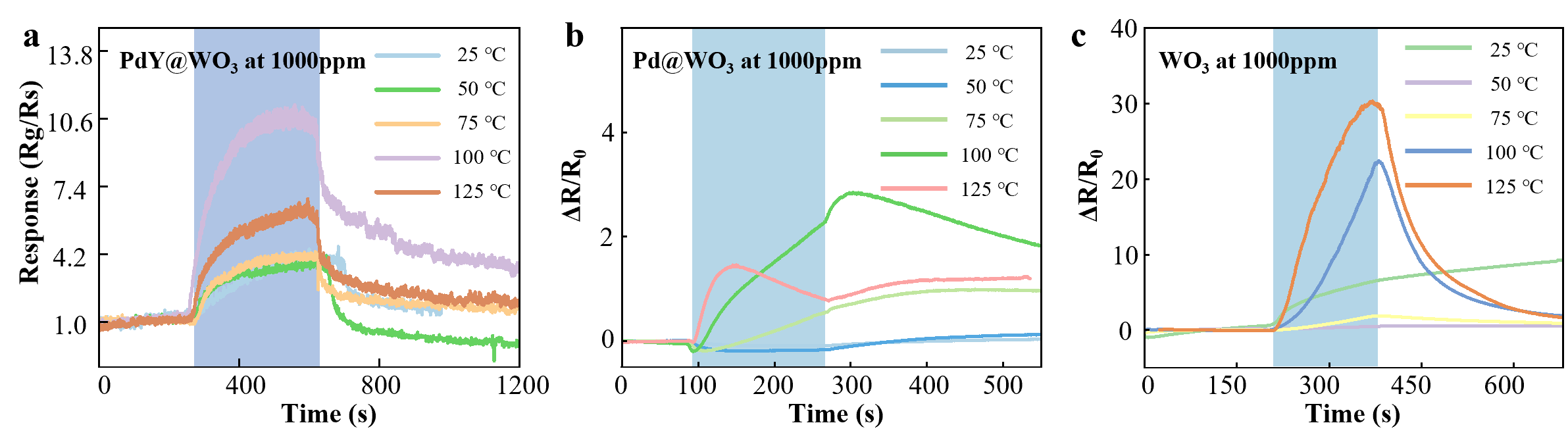


Fig. S15. Hydrogen response performance of the EMSA-gas module with **a,** PdY@WO_3_, **b,** Pd@WO_3_, and **c,** WO_3_ sensing layers at different temperatures.


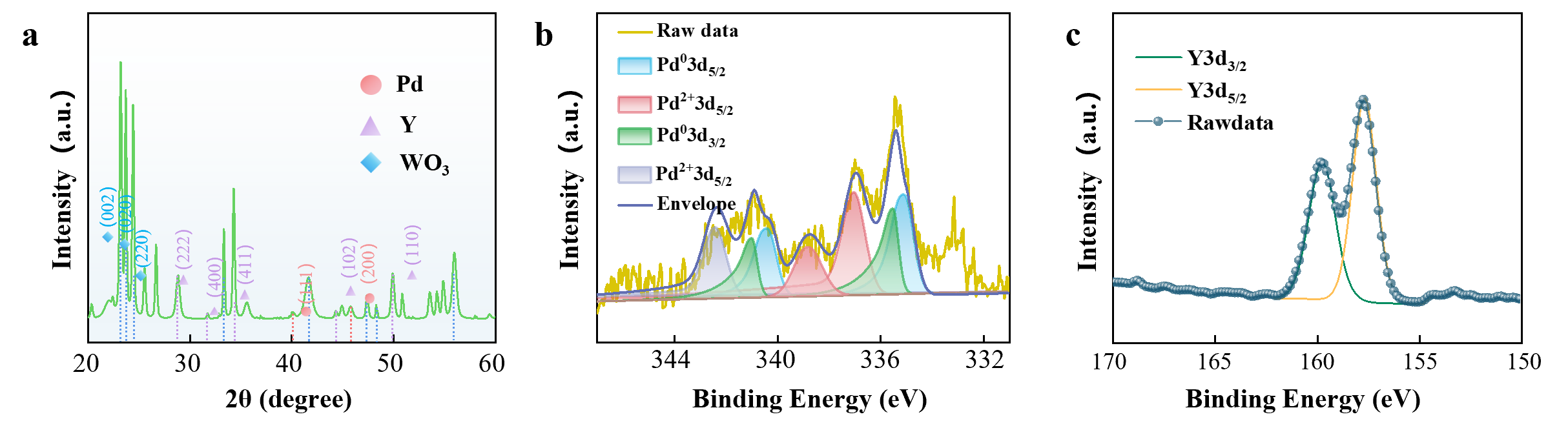


Fig. S16. **a,** XRD pattern. **b** and **c,** XPS spectrum of the EMSA-gas sensing layer.


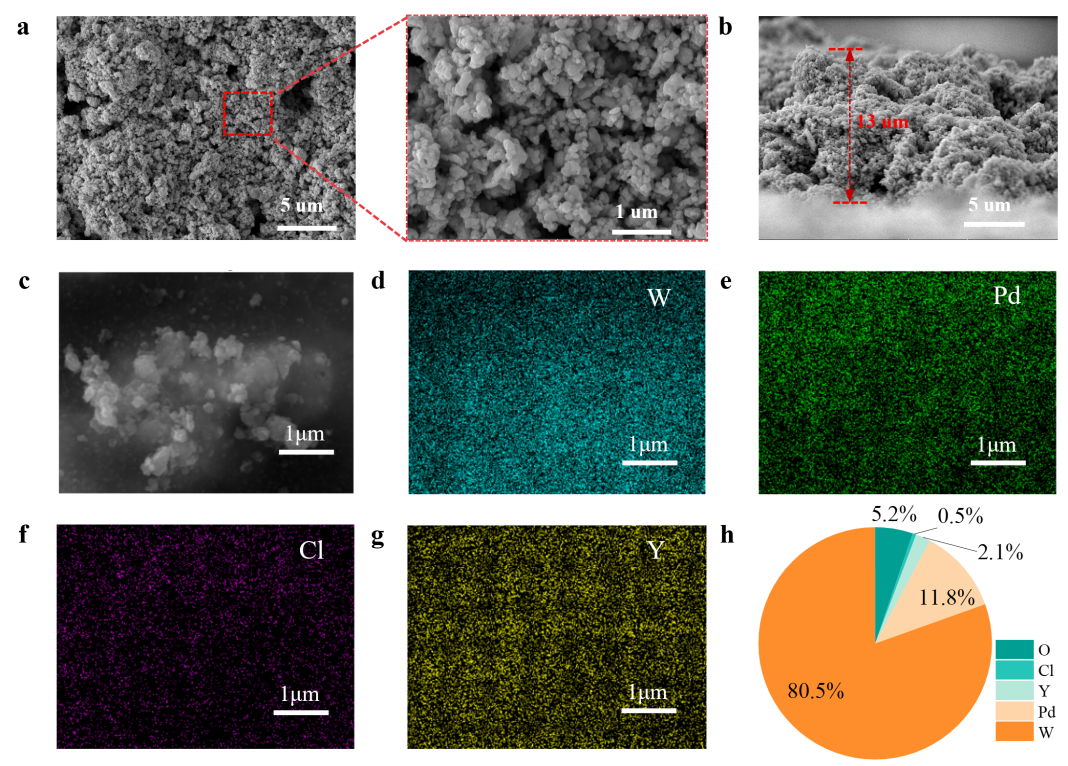


Fig. S17. SEM images of the PdY@WO_3_ gas sensing layer: **a,** Top-view; **b,** Cross-sectional view; **c,** High-magnification SEM image; and corresponding EDS elemental mapping of **d,** W, **e,** Pd, **f,** Cl, and **g,** Y element. **h,** Elemental composition analysis of the gas sensing layer.

Characterization and analysis of PdY@WO3

The bulk crystal structure of Pd/Y alloy-loaded WO_3_ was analyzed using XRD, revealing the crystal phases of the gas-sensitive layer. Fig. S16a (JCPDS No. 32-1395) shows WO_3_ peaks at 22.9°, 25.5°, and 35.5°, with corresponding sub-peaks. Four distinct diffraction peaks of the Pd alloy particles are observed at 39.8° and 46.2°, corresponding to the (111) and (200) crystal planes, respectively. Y alloy particles show peaks at 28.5°, 32.5°, and 34.5°, corresponding to the (222), (400), and (411) planes. XPS (Fig. S16b) shows Pd 3d₅/₂ main and satellite peaks at 335.3 and 340.3 eV, and Pd 3d₃/₂ peaks at 341.2 and 335.7 eV, confirming the existing of Pd⁰. ^[5]^ PdY@WO3 also shows Pd²⁺ 3d₅/₂ peaks at 337.8 and 339.2 eV, and 3d₃/₂ peaks at 342.9 eV ^[6]^. The two strong sub-peaks at 158.4 and 156.7 eV correspond to the high binding energy of Y 3d₃/₂ and the low binding energy of Y 3d₅/₂, respectively, confirming the presence of Y in the sample. ^[7]^ Minor Pd^2+^ and Y^2+^ species also appear, ascribed to incomplete reduction of PdCl_2_ and YCl_2_,. The presence of small peaks corresponding to palladium oxide suggests secondary oxidation during the fabrication process in the tube furnace. Fig. S17 illustrates the SEM morphology of PdY@WO_3_. The material exhibits a uniform distribution of nanoparticles with a thickness of approximately 13 μm. A porous stacking increases surface area and gas adsorption, enhancing sensor response. Furthermore, EDS mapping (Fig. S17c) confirms uniform Pd and Y dispersion. Combining the Map Sum Spectrum (Fig. S17d), it is evident that, in addition to Pd and Y, a small amount of Cl is present, which is consistent with the XPS spectrum (Fig. S16b and c). These results confirm successful fabrication of Pd/Y-modified WO_3._


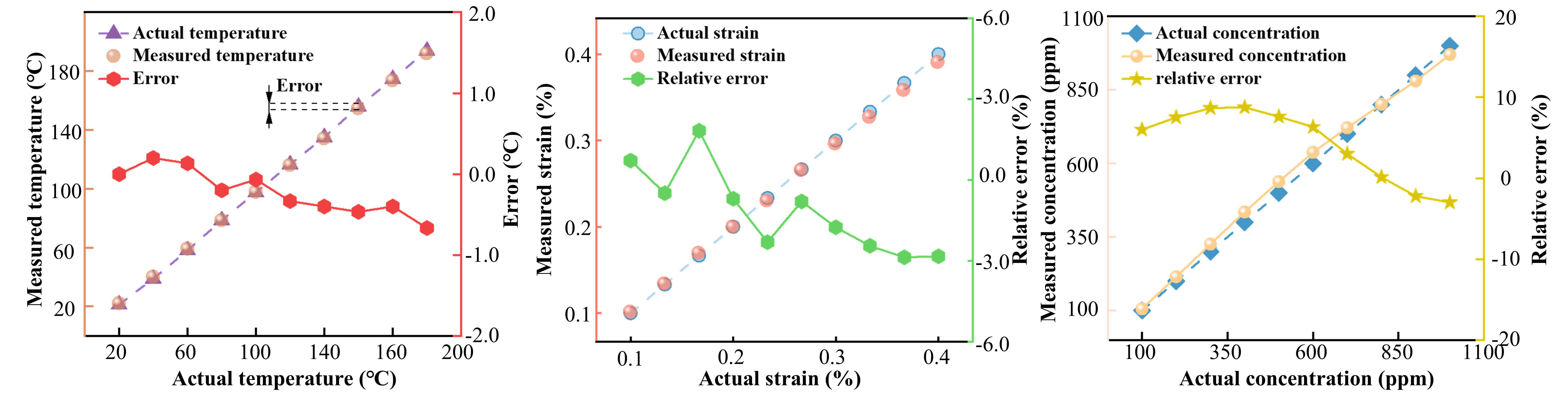


**Fig. S18.**Hydrogen response accuracy test of EMSA-gas.


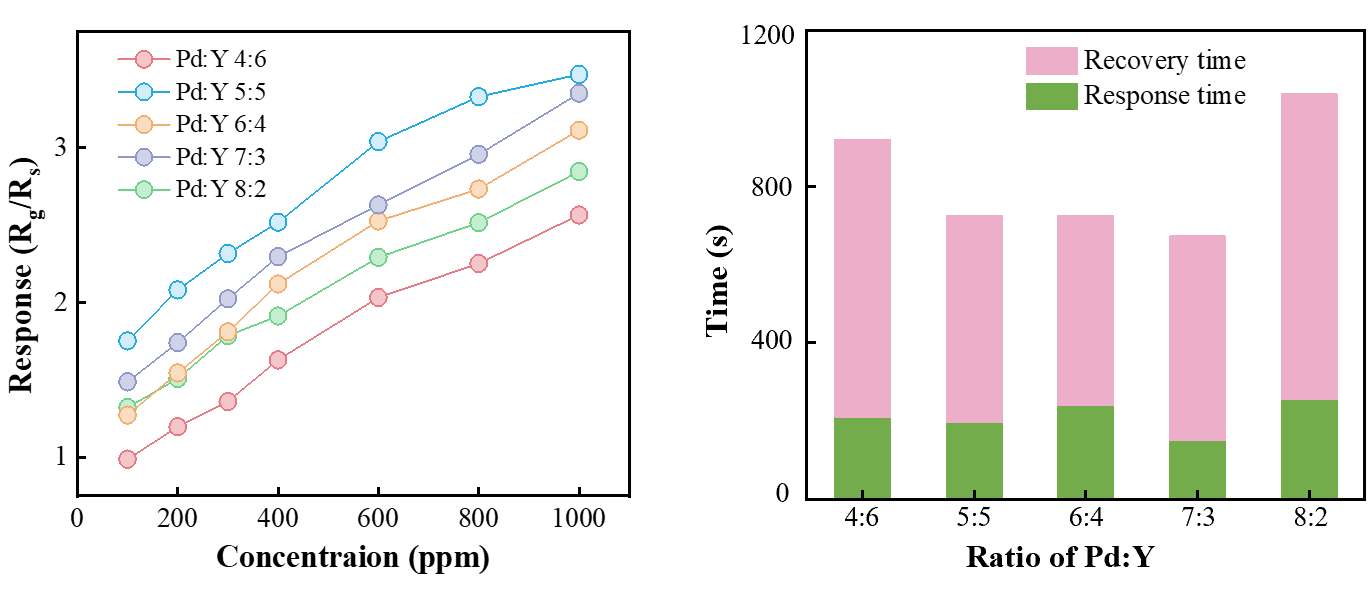


Fig. S19. **a,** Hydrogen response of the EMSA-gas module with different Pd/Y weight ratios. **b,** Response and recovery time of the EMSA-gas module with different Pd/Y weight ratios at 1000 ppm hydrogen.


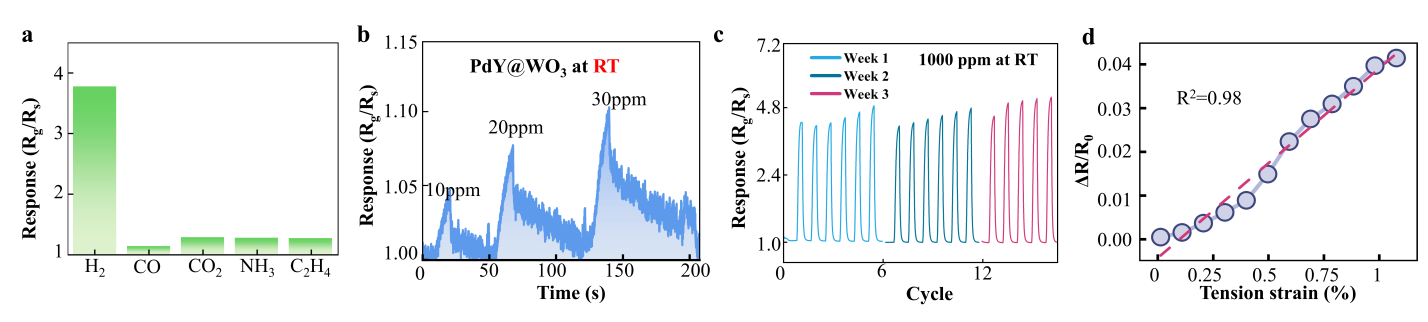


Fig. S20. **a,** Gas selectivity of the EMSA-gas. **b,** Response of the EMSA-gas at low hydrogen concentrations. **c,** Long-term stability testing of EMSA-gas. **d,** Strain-induced crosstalk to EMSA-gas.

**Table S2.** Quantified cross-talk of each EMSA sensing layer across the operational working range.

|  | **EMSA-tem** | **EMSA-mech** | **EMSA-gas** |
| --- | --- | --- | --- |
| **Temperature** | / | 1.36×10^-6^ ℃^-1^ | (141e^(T/46.2)^+157) ℃^-1^  (Need compensation) |
| **Strain** | No response | / | Negligible |
| **Hydrogen** | No response | No response | / |

**Quantification of Cross-talk Between Three-Modal Signals**

The operational range for early TR warning was defined as 60–100 °C (associated with SEI breakdown ^[8]^) and strains below 1500 με. (Figs. 4 and 5). The maximum crosstalk within this range is quantified in Table S2.

For the EMSA-tem, which operates based on the Seebeck effect (Fig. 2e), the signal is inherently independent of mechanical strain and gas exposure. Consequently, neither strain nor hydrogen induces any significant response in this module.

The EMSA-mech has a sensitivity of 44.4 in response to mechanical deformation, but owns a low temperature sensitivity of 60.1 and -120.2 ppm·℃^-1^ within 20-100 and 100-200 ℃, respectively, as shown in Fig. 2h. Thus, within the LIB early warning temperature range, temperature-strain cross-sensitivity coefficient is to be (60.1 ppm ℃^-1^/44.4= 1.36 ppm ℃^-1^).

For the EMSA-gas, similarly, the sensitivity to hydrogen concentration is determined to be *S_h_*=0.0023 ppm^-1^ (Fig.2n). The temperature dependence of the EMSA-gas can be fitted by the equation *R_a_/R_g_*=0.325e(^T/46.2^)+0.37. (Fig.2q) Therefore, the temperature-gas cross-sensitivity coefficient is to be (0.325e^(T/46.2)^+0.37)/0.0023 ℃^-1^. At 110 °C, the maximum resistance shift corresponds to a response (R_g_/R_s_) of ~3.88, equivalent to the signal from ~1148.66 ppm of H_2._ Thus, temperature compensation is essential. Regarding strain-induced crosstalk (as shown in Fig. R6), the resistance increases linearly with deformation ((R_strain_-R_0_)/(εR_0_)=*GF*=4, *R^2^*=0.98). When both gas concentration C and strain ε are present, the actual resistance R is:

 (1)

where R0 is the calibrated initial resistance. (no gas, no strain) In gas measurement, a fixed R0 is used as *R_g_*, the measured resistance *R* is used, and the apparent concentration C*_apparent_* is calculated:

 (2)

Substituting the actual R of equation(1) into equation(2) and simplifying,we can get the equation(3)

 (3)

The relative error is showed in equation(4)

 (4)

To simplify calculations, we assess the magnitude of crosstalk by evaluating the change in hydrogen concentration corresponding to the maximum working strain during EMSA. At 1500 με, according to equation(4), the relative error is ~-0.006. At a hydrogen concentration of 1000 ppm, crosstalk is merely -6 ppm, which is negligible.

**Table S3.** Comparison of the decoupling capability between the EMSA and other reported sensors.

| **Type** | **T-S coefficient** | **S-T**  **coefficient** | **T-G**  **coefficient** | **G-T**  **coefficient** | **G-S**  **coefficient** | **S-G**  **coefficient** | **Ref** |
| --- | --- | --- | --- | --- | --- | --- | --- |
| T-S-G | 1.36 ppm ℃^-1^ | No | (141e^(T/46.2)^+157) ℃^-1^ | No | No | Negligible | This work |
| T-G | / | / | -0.08858 ℃^-1^ | -11.288 ppm^-1^ | / | / | ^[9]^ |
| T-G | / | / | 0.000154 ℃^-1^ | 6439 ppm^-1^ | / | / | ^[10]^ |
| T-S | 0.596 ppm ℃^-1^ | No | / | / | / | / | ^[11]^ |
| T-S | -4071 ppm ℃^-1^ | -240 | / | / | / | / | ^[12]^ |
| S-G |  |  |  |  | 0.0175 ppm^-1^ | 57.1 | ^[13]^ |

**Table S4.** Response time of each sensing layer of EMSA and comparison with other multimodal sensors.

| **Type** | **Temperature (s)** | **Strain (ms)** | **Gas (s)** | **Ref** |
| --- | --- | --- | --- | --- |
| T-S-G | 8 | <8 | < 189 | This work |
| T-S | 1 | 100~200 | / | ^[14]^ |
| T-S | >200 | <39 | / | ^[15]^ |
| T-S | 9 | < 170 | / | ^[16]^ |
| T-S | >15 | 1500 |  | ^[17]^ |
| T-G | >50 |  | 217 | ^[9]^ |
| T-G | >15.3 |  | <20 | ^[10]^ |
| S-G | / | 800 | 36 | ^[13]^ |
| S-G |  | 616 | 150 | ^[18]^ |

In conclusion, the EMSA exhibits great and stable single-parameter response in each sensing layer. For a multimodal sensor, however, resistance to inter-signal cross-talk is equally critical. As quantified in Table S2, the EMSA demonstrates exceptional resistance to cross-talk, which arises from its distinct sensing mechanisms and performance tuning. Most signal pairs, such as strain-to-temperature and gas-to-temperature, show negligible interference. The primary cross-talk occurs from temperature to gas response. A systematic comparison with other multimodal sensors (Table S3) reveals that the T–S coefficient of the EMSA is only slightly higher than that reported by Li et ^[11]^ al. and lower than the S–G cross-talk index reported by Tan et al. ^[13]^. Nevertheless, the T–G coefficient remains notably higher than values in references^[19]^, indicating that accurate gas concentration measurement requires temperature compensation.

**Table S5.** Comprehensive performance comparison between EMSA and other multimodal sensing systems

| **Device** | **Parameter** | **EMSA** | **Fiber optical sensor** | **Commercial BMS** |
| --- | --- | --- | --- | --- |
| **Response time** | Temperature: | < 8 s | 0.3~30 s | 0.2~12 s |
|  | Strain | < 8 ms | / | / |
|  | Gas | < 195 s | 1~10 s | <30 s |
| **Accuracy** | Temperature: | ±1 ℃ | ±0.1~±1 ℃ | ±0.2~±2 ℃ |
|  | Strain | ± 3% | ±0.3~±2% | / |
|  | Gas | ± 10% | ±3% | ±5-20 % |
| **Integration complexity** | Components | Thin film sensor  +  Multimodal data acquisition module  (wireless) | Fiber optic sensor  +Demodulator  (Wired) | Sensors  +  Motherboard +  Signal bus  (Wired) |
|  | Complexity | Low | Middle | High |

In terms of overall response performance (Table S4), the strain response is particularly rapid (<8 ms), significantly outperforming most reported T–S and S–G sensors. The temperature response (~8 s) remains highly competitive, albeit slightly slower than the T–S sensor by Xiao et al. ^[14]^. Although the gas response is slower (<189 s), it still surpasses the result reported by Yang et al. ^[9]^, underscoring the well-balanced performance of the EMSA system.


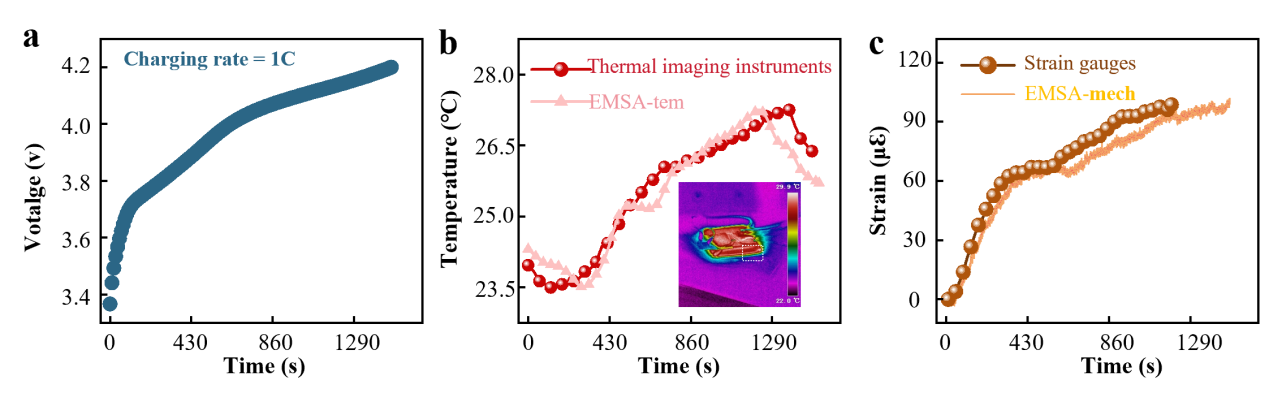


Fig. S21. **a,** Voltage profile of a lithium-ion battery at a 1C charge rate. **b,** Surface strain of the LIBs measured by the EMSA and a strain gauge at 1C. **c,** Surface temperature of the LIBs measured by the EMSA and an infrared thermography at 1C.


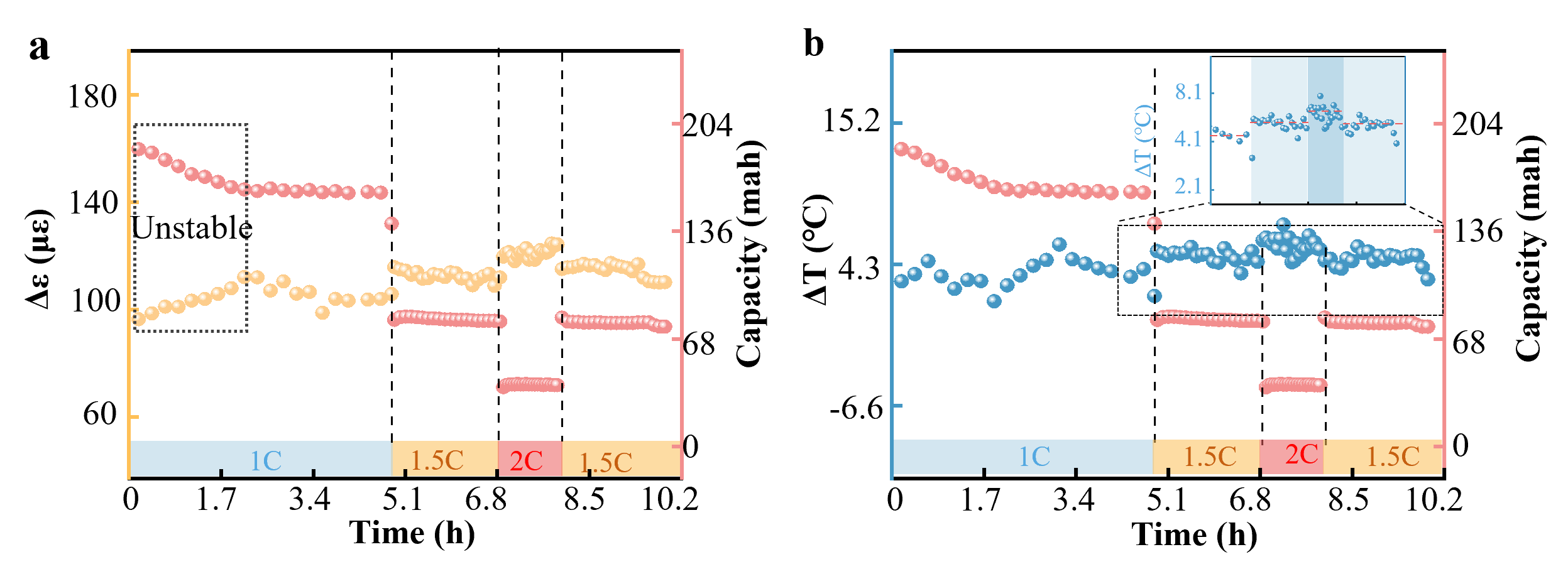


Fig. S22. **a,** Strain amplitude measured by the EMSA at different charge rates. **b,** Temperature amplitude measured by the EMSA at different charge rates.


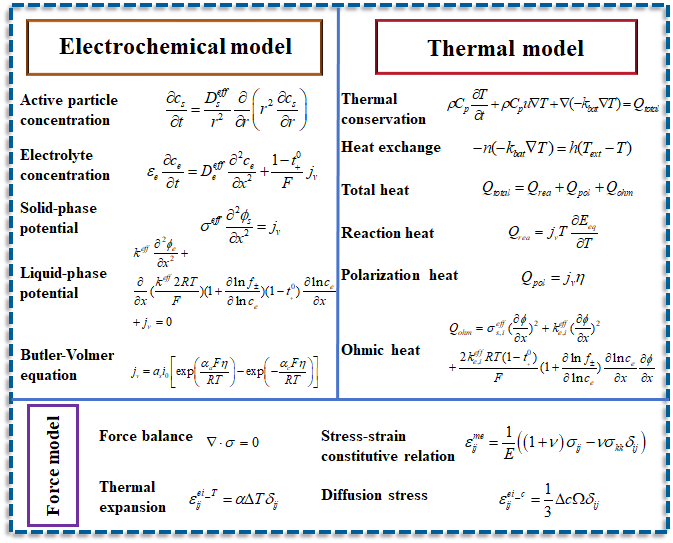


Fig. S23. Electrochemical-thermal-Mechanical coupling model of LiFePO₄ pouch cell under normal operation.

**E-T-M Model Construction**

The electrochemical submodel uses a pseudo-two-dimensional (P2D) framework to resolve ion transport and reaction kinetics (architecture in Fig. S23). The thermal submodel incorporates reaction-generated heat, cell-to-environment exchange, and internal conduction to map temperature fields, while the mechanical submodel computes stresses arising from material deformation, thermal expansion, and concentration gradients. These three modules exchange key variables—electrochemical heat feeds the thermal solver; temperatures adjust reaction rates; concentration and temperature gradients drive mechanical loads; the resulting stresses, in turn, modify reaction kinetics and heat generation.


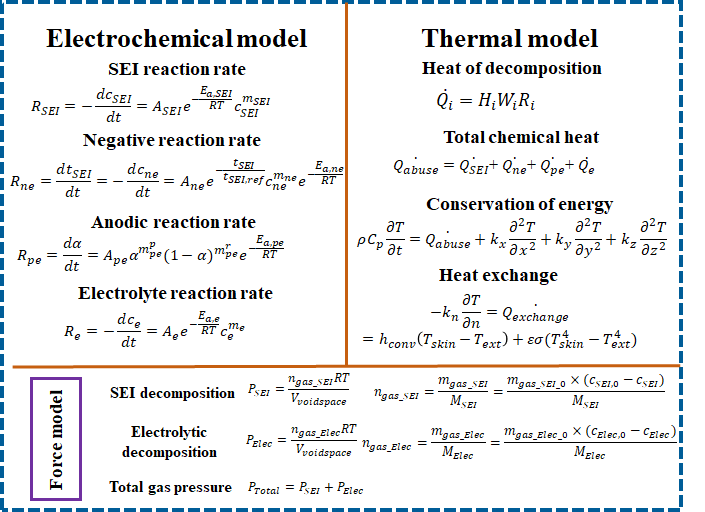


Fig. S24. Electrochemical-thermal-pressure coupling model of LiFePO₄ Pouch cell under TR.

**The Principle of the E-T-P Model**

The modeled exothermic processes include SEI layer decomposition, melting of electrode materials, and electrolyte vaporization. The total chemical heat release Qabuse is incorporated as a source term into a three-dimensional thermodynamic model, which comprehensively accounts for internal heat generation and conduction, heat exchange with the environment, and thermal radiation effects, and pressure changes caused by gas production.


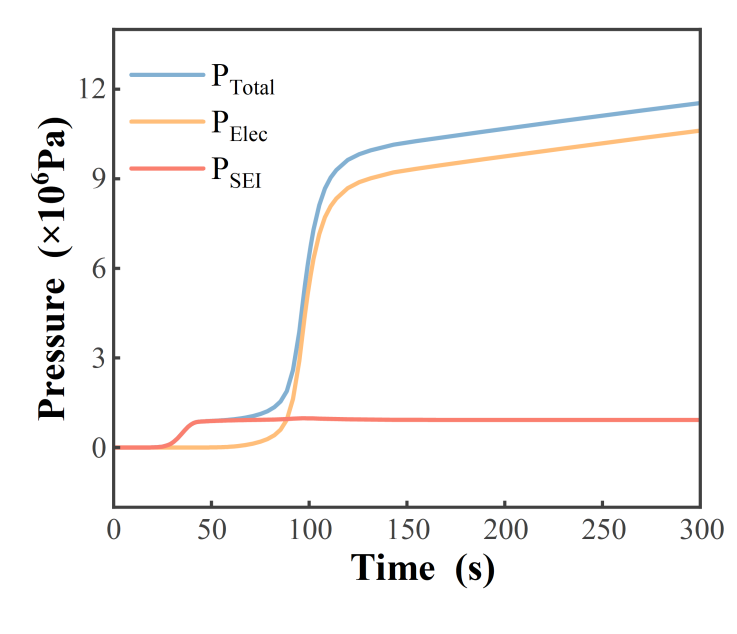


Fig. S25. Results of internal battery pressure changes during TR.


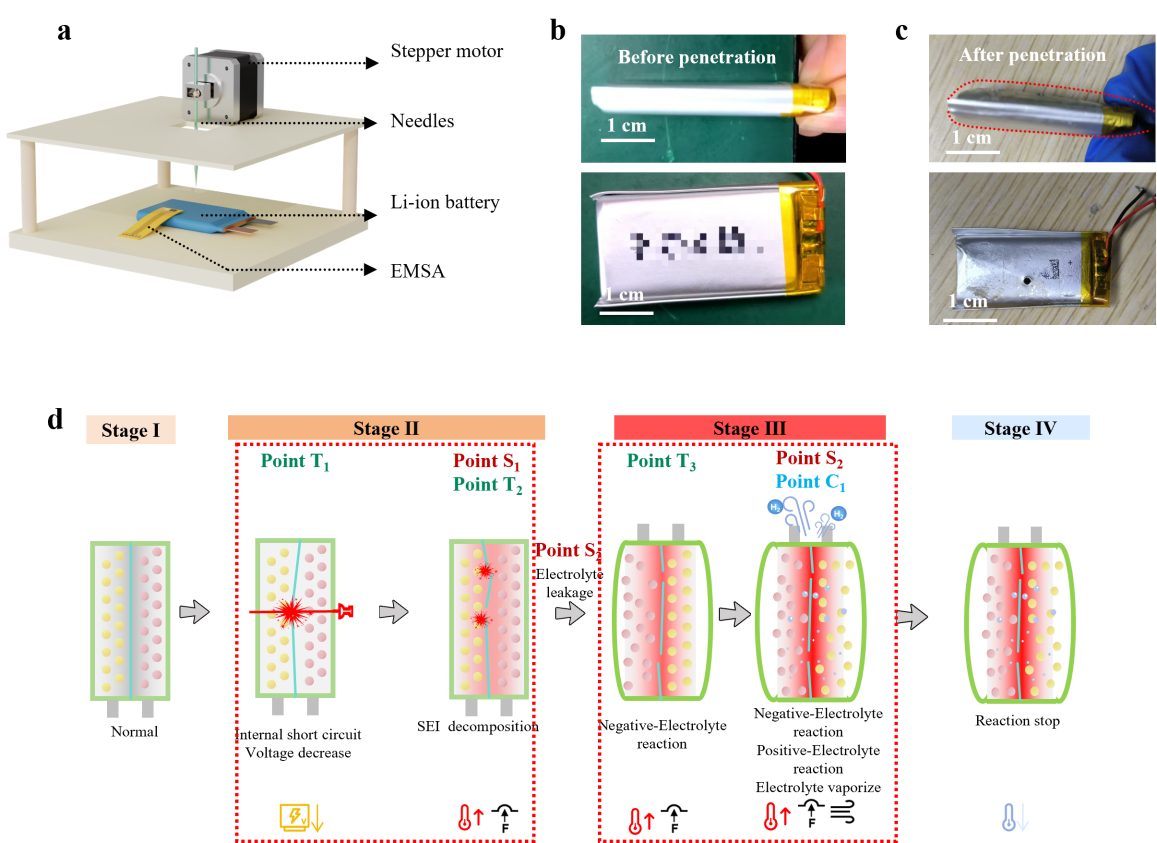


Fig. S26. **a,** Experimental setup for a puncture-induced TR of lithium-ion batteries. **b** and **c,** Photographs of a lithium-ion battery after the puncturing experiment. **d,** The E-T model-predicted sequence of internal reactions coincides with key multimodal signal transitions, enabling identification of the early warning period for mechanical-induced TR


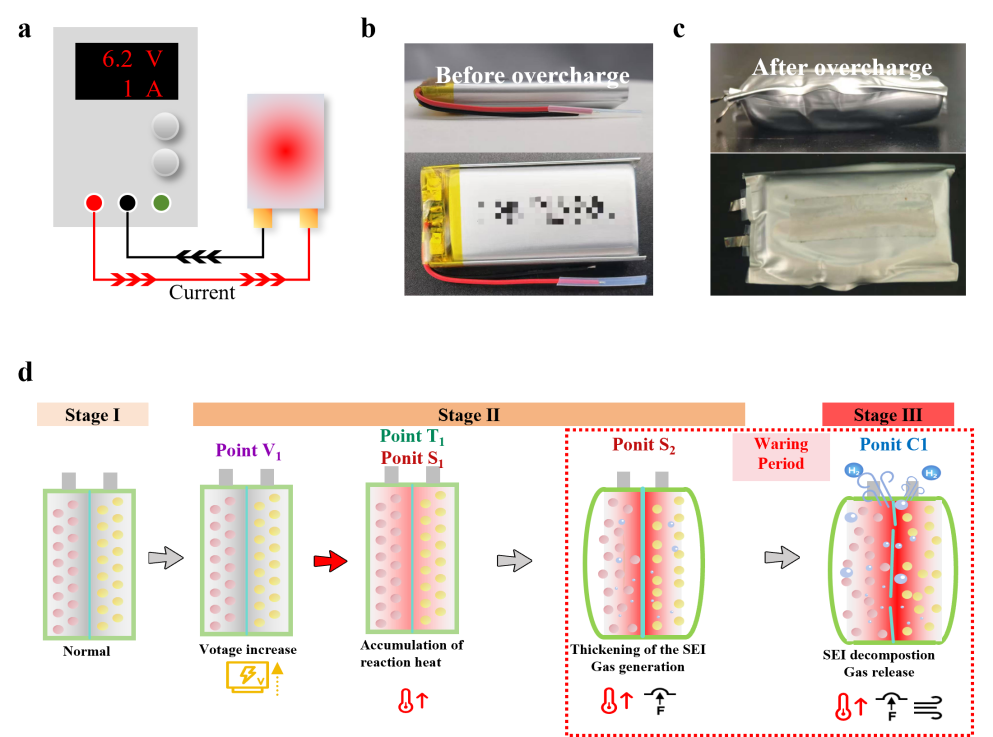


Fig. S27. **a,** Experimental setup for overcharge TR of LIBs. Comparison of LIB appearance **b,** before and **c,** after overcharge. **d,** The E-T model-predicted sequence of internal reactions coincides with key multimodal signal transitions, enabling identification of the early warning period for electrical-induced TR


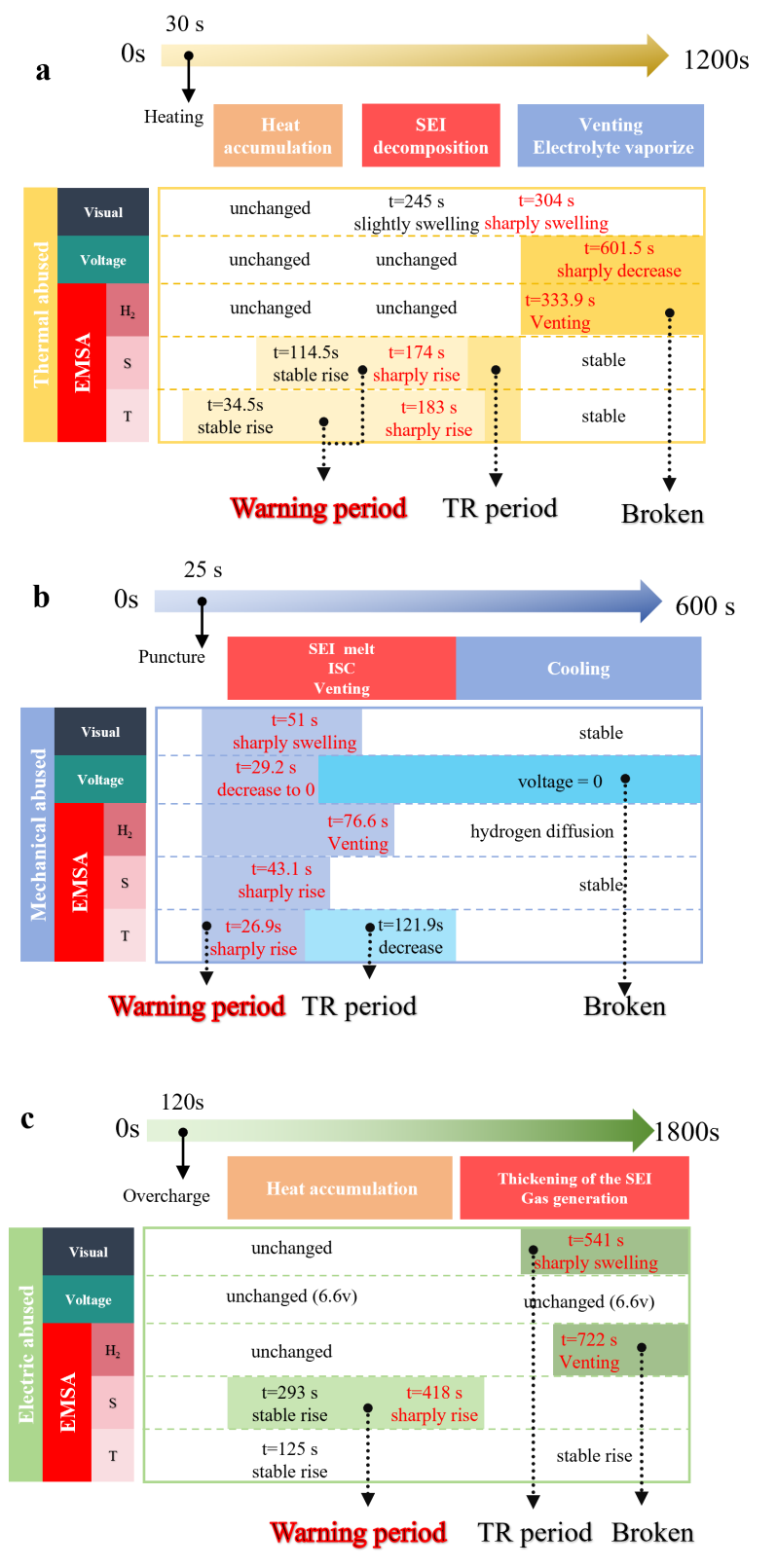


Fig. S28. EMSA-based early-warning windows and reaction stages for lithium-ion battery TR under a, thermal, b, mechanical, and c, electrical abuses.

Movie S1. Multimodal online monitoring of lithium-ion batteries under thermal abuse-induced thermal runaway based on EMSA (20× speed; the thermal runaway early-warning window was intentionally

**Movie S2**. Multimodal online monitoring of lithium-ion batteries under electrical abuse-induced thermal runaway based on EMSA (10× speed; the thermal runaway early-warning window remained active

**Movie S3.** Multimodal online monitoring of lithium-ion batteries under electrical abuse-induced thermal runaway based on EMSA (30× speed; the thermal runaway early-warning window was intentional
