# Supplementary figures and images for: A Self-Decoupling Multimodal Sensor for Enhanced Early Warning of Lithium-Ion Battery Thermal Runaway
Source: Research (Wash D C). 2026 Feb 24;9:1120. doi: 10.34133/research.1120 (PMC12929815; doi:10.34133/research.1120)

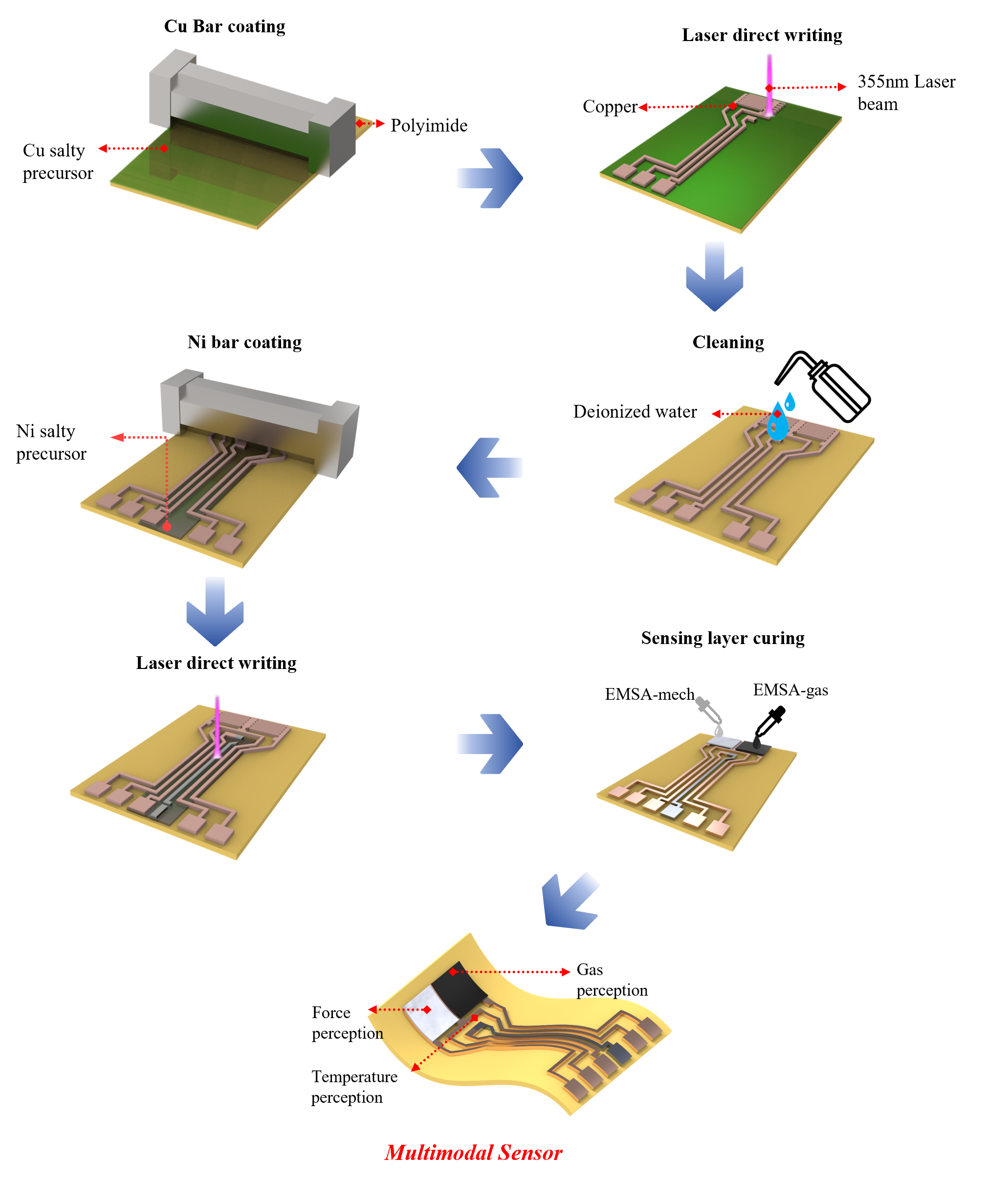

Supplement: Supplementary 1 — Figs. S1 to S28 Tables S1 to S5 Movies S1 to S3 [file research.1120.f1.zip › Fig.S1.tif]

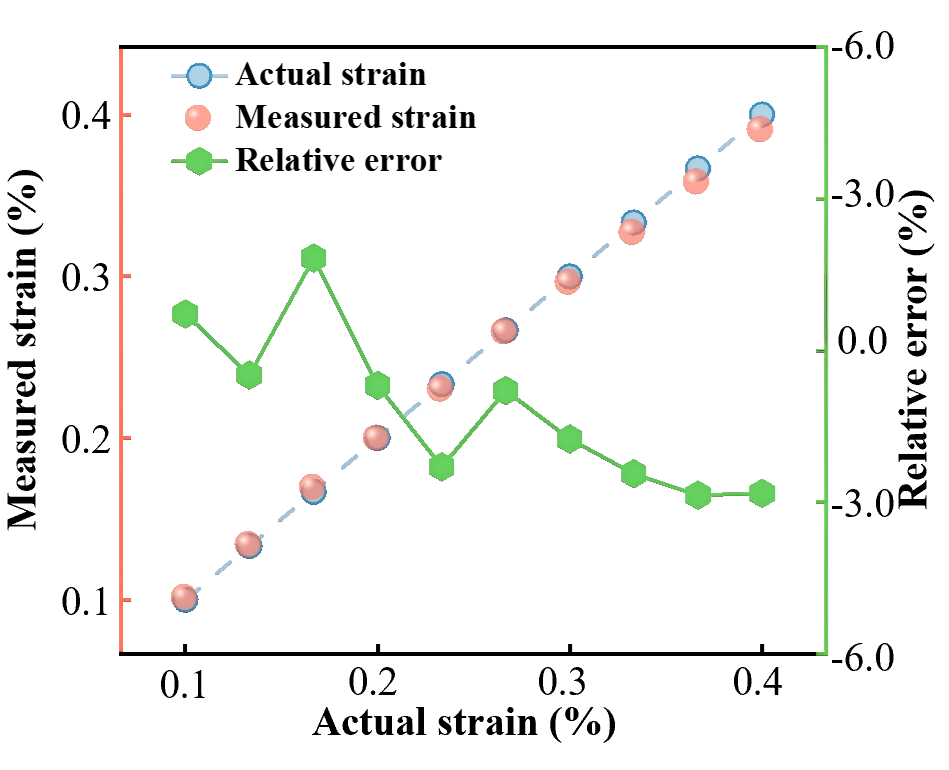

Supplement: Supplementary 1 — Figs. S1 to S28 Tables S1 to S5 Movies S1 to S3 [file research.1120.f1.zip › Fig.S10.png]

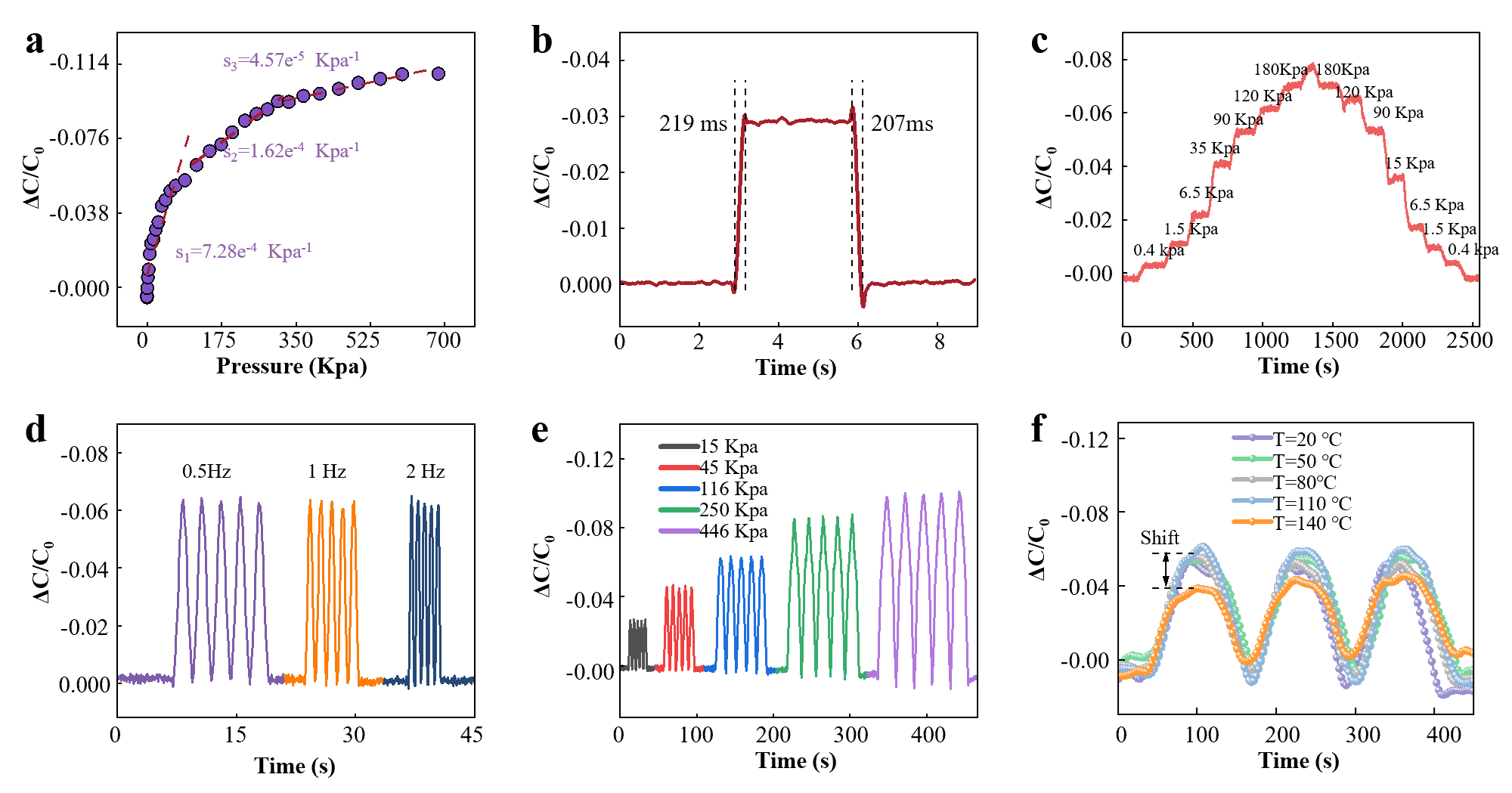

Supplement: Supplementary 1 — Figs. S1 to S28 Tables S1 to S5 Movies S1 to S3 [file research.1120.f1.zip › Fig.S12.tif]

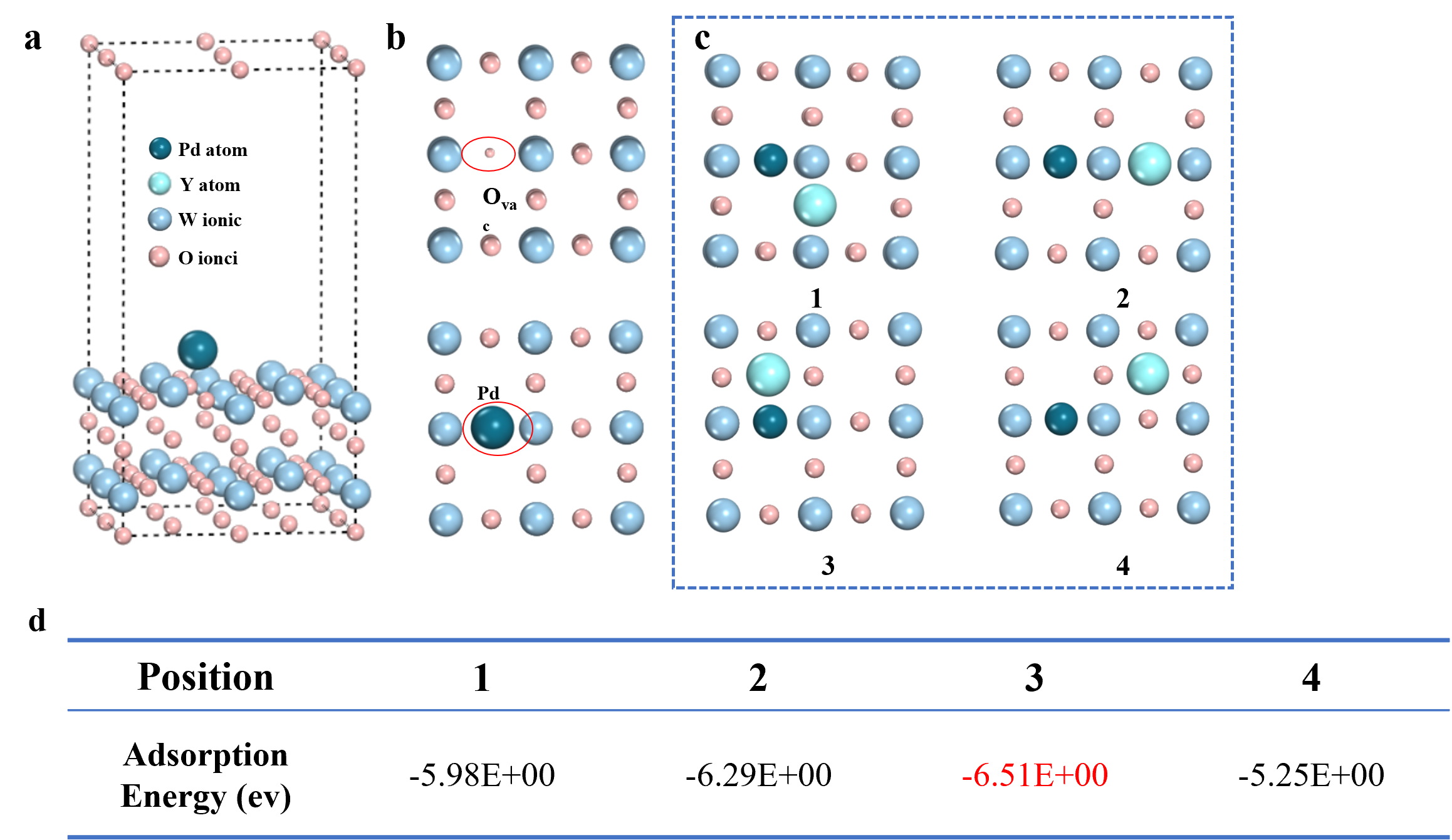

Supplement: Supplementary 1 — Figs. S1 to S28 Tables S1 to S5 Movies S1 to S3 [file research.1120.f1.zip › Fig.S13.tif]

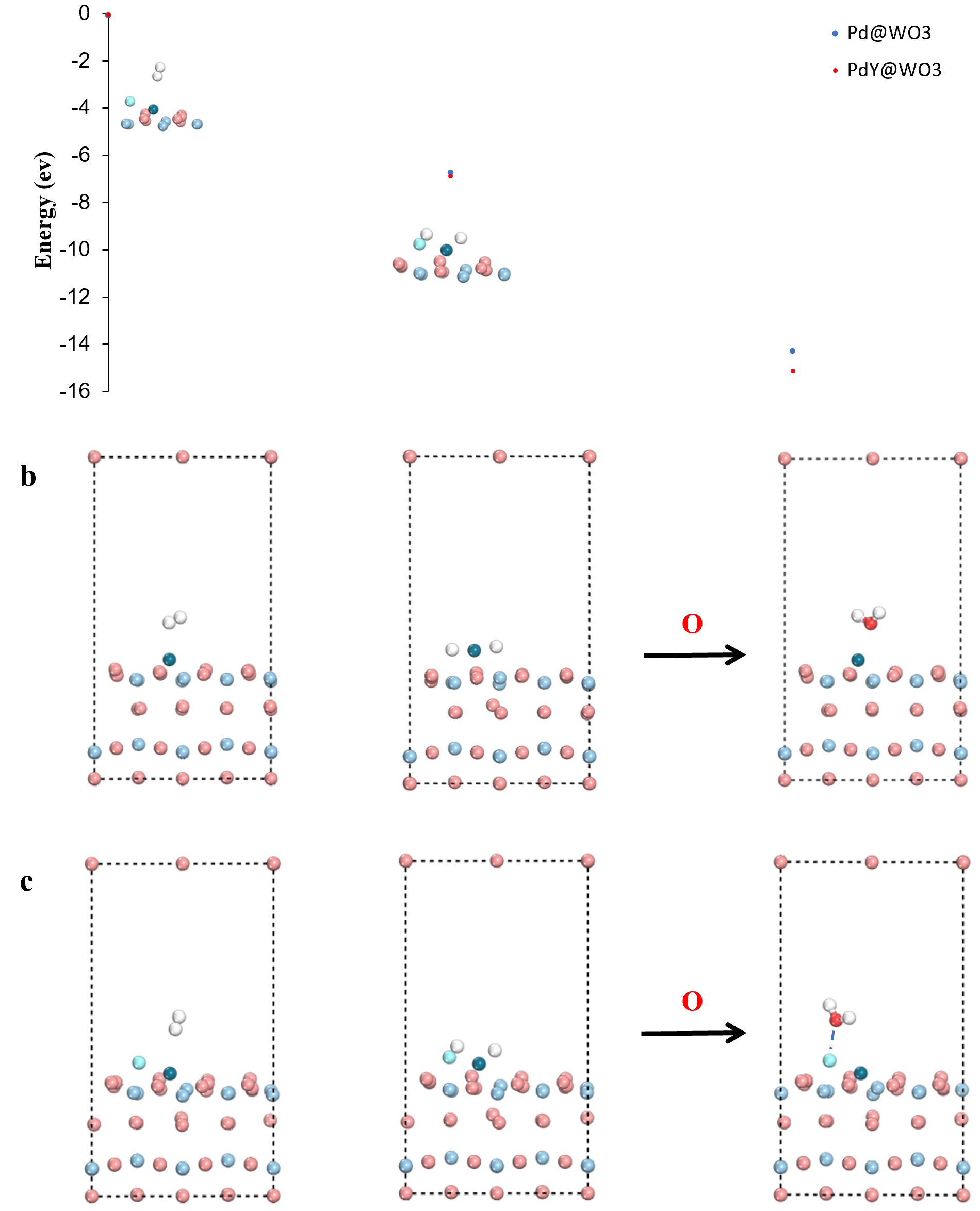

Supplement: Supplementary 1 — Figs. S1 to S28 Tables S1 to S5 Movies S1 to S3 [file research.1120.f1.zip › Fig.S14.tif]

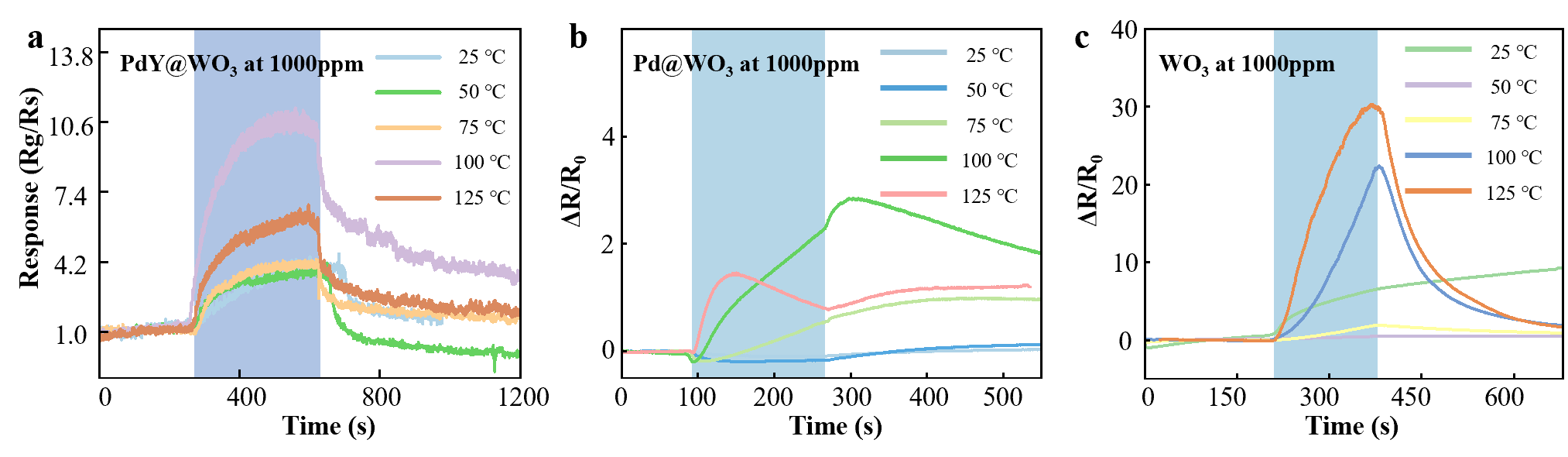

Supplement: Supplementary 1 — Figs. S1 to S28 Tables S1 to S5 Movies S1 to S3 [file research.1120.f1.zip › Fig.S15.tif]

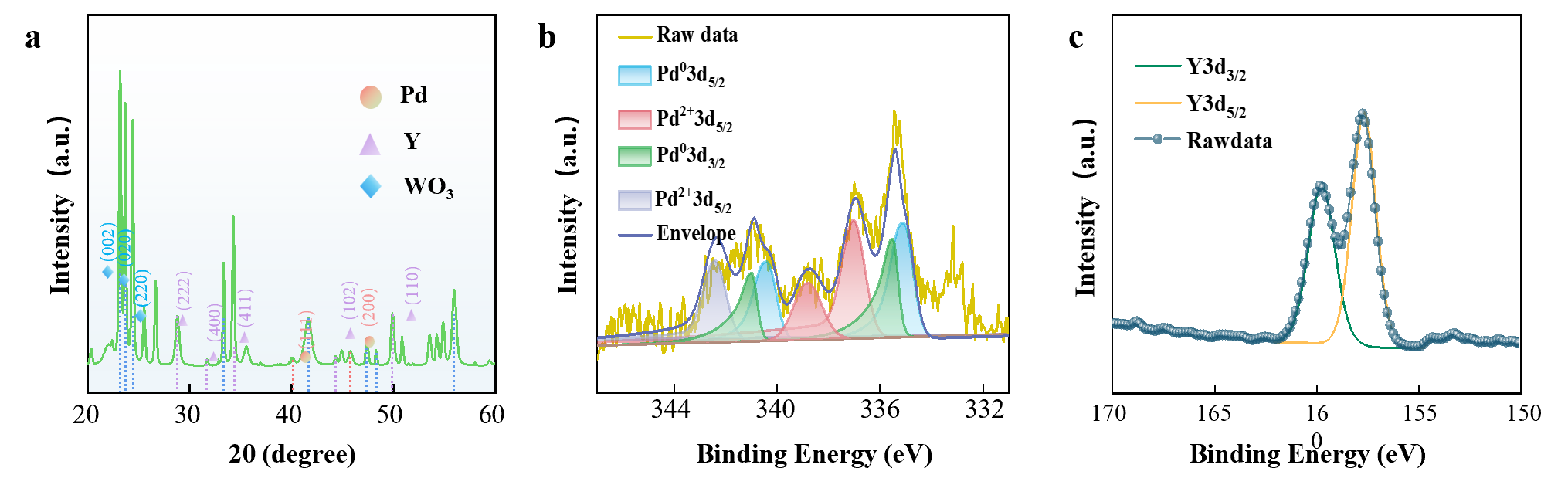

Supplement: Supplementary 1 — Figs. S1 to S28 Tables S1 to S5 Movies S1 to S3 [file research.1120.f1.zip › Fig.S16.tif]

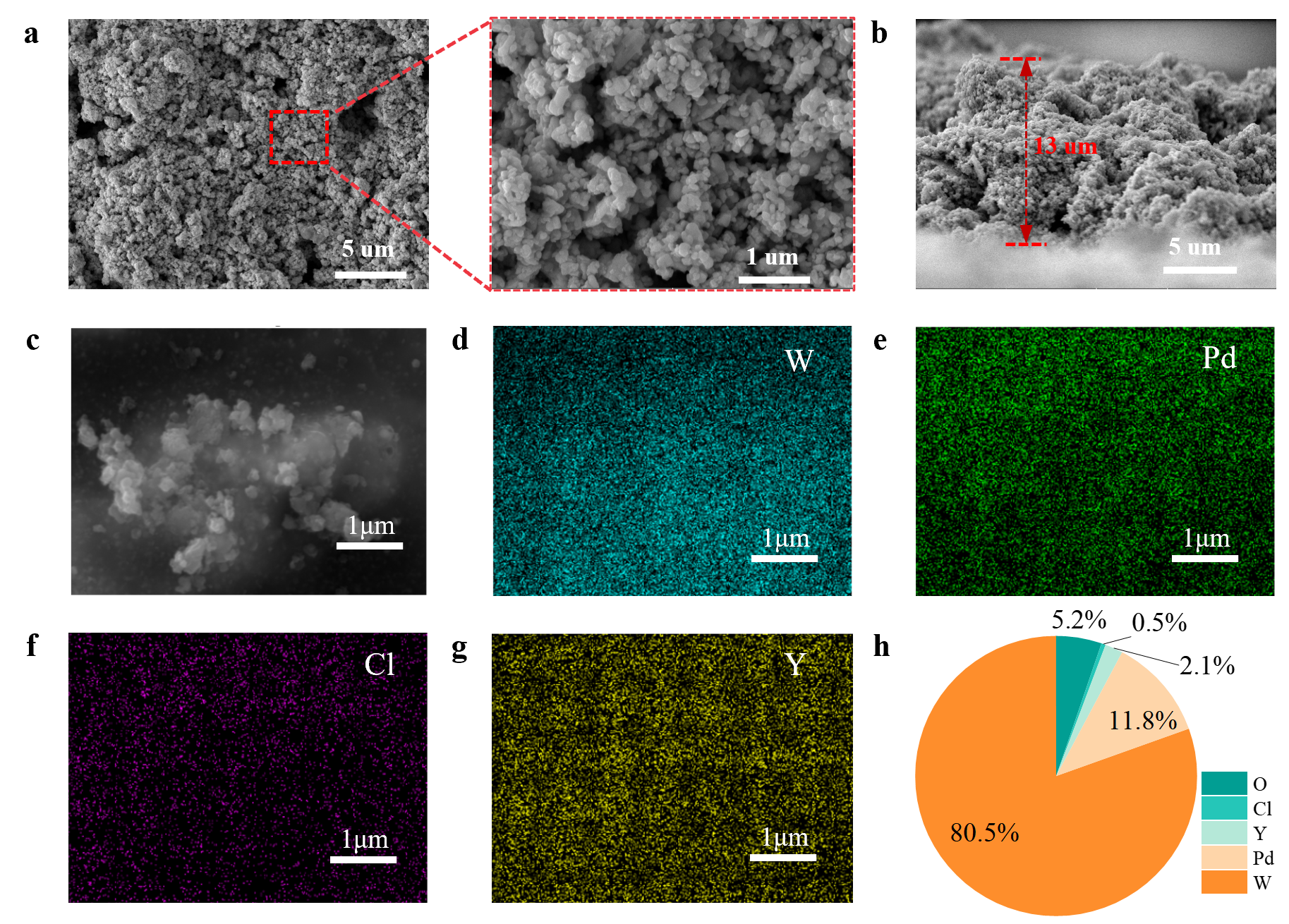

Supplement: Supplementary 1 — Figs. S1 to S28 Tables S1 to S5 Movies S1 to S3 [file research.1120.f1.zip › Fig.S17.tif]

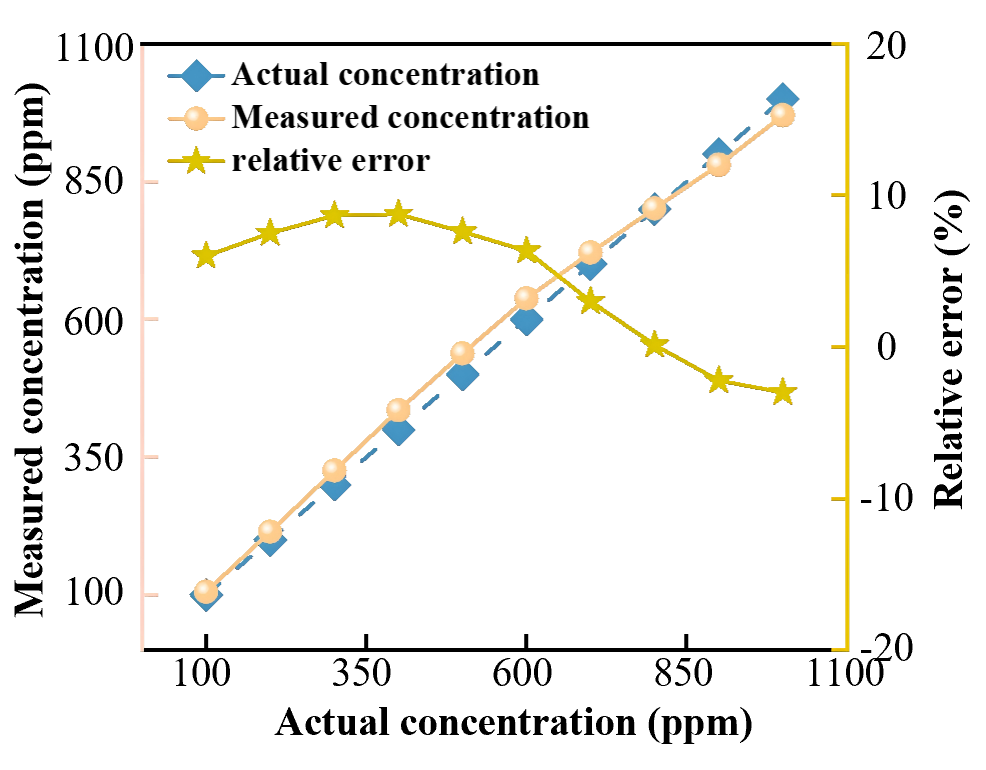

Supplement: Supplementary 1 — Figs. S1 to S28 Tables S1 to S5 Movies S1 to S3 [file research.1120.f1.zip › Fig.S18.png]

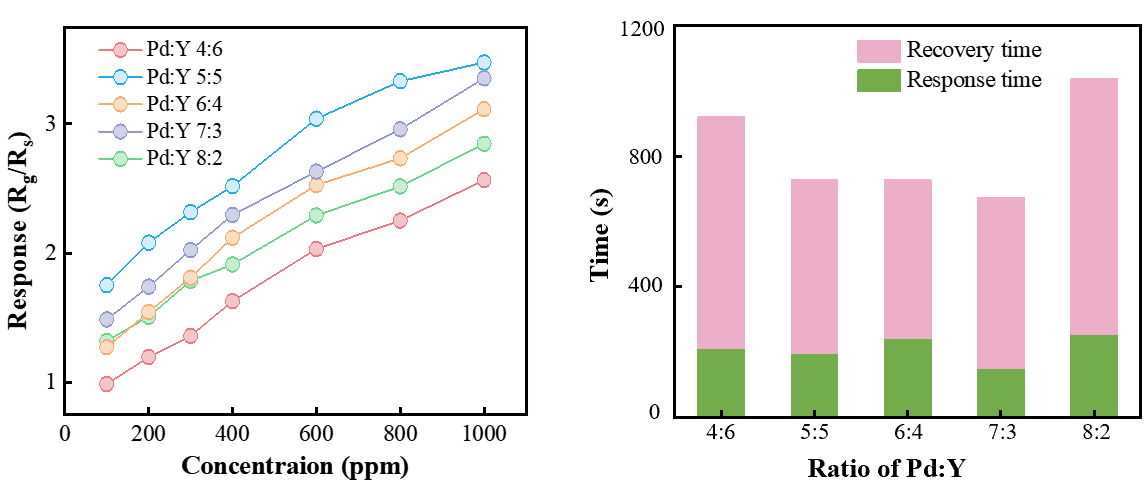

Supplement: Supplementary 1 — Figs. S1 to S28 Tables S1 to S5 Movies S1 to S3 [file research.1120.f1.zip › Fig.S19.tif]

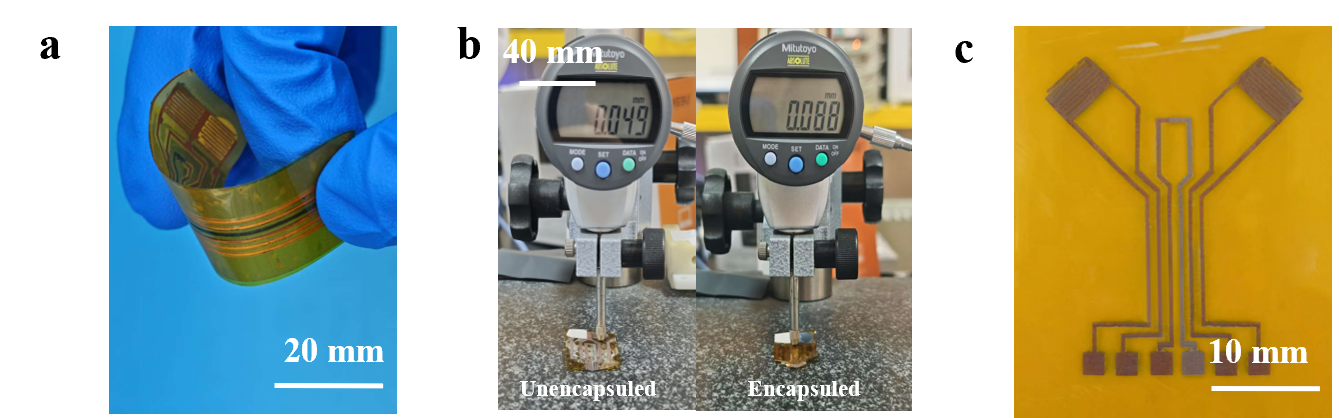

Supplement: Supplementary 1 — Figs. S1 to S28 Tables S1 to S5 Movies S1 to S3 [file research.1120.f1.zip › Fig.S2.tif]

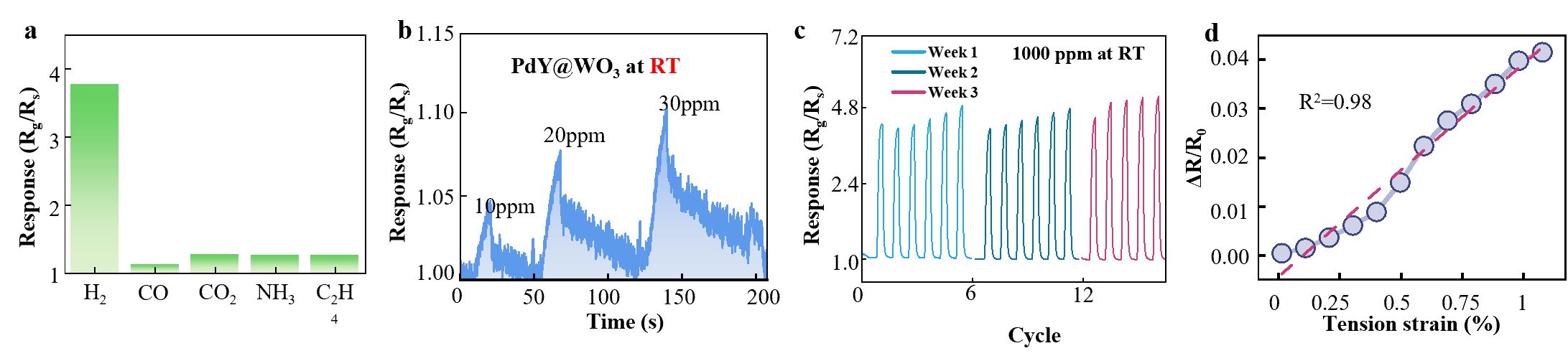

Supplement: Supplementary 1 — Figs. S1 to S28 Tables S1 to S5 Movies S1 to S3 [file research.1120.f1.zip › Fig.S20.tif]

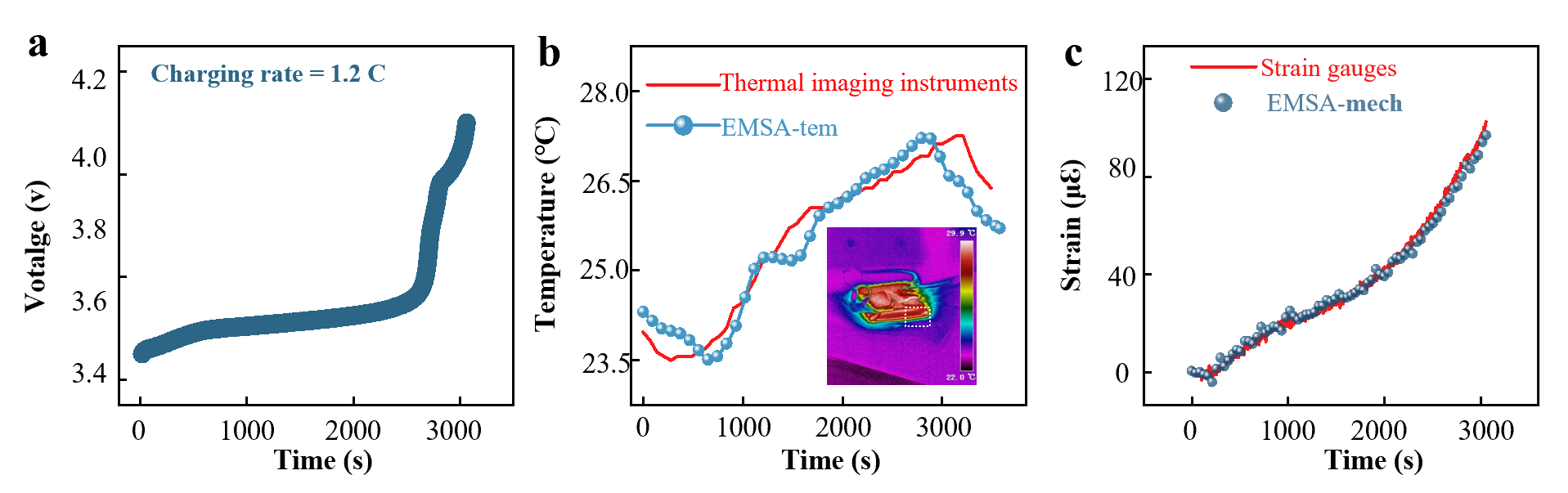

Supplement: Supplementary 1 — Figs. S1 to S28 Tables S1 to S5 Movies S1 to S3 [file research.1120.f1.zip › Fig.S21.png]

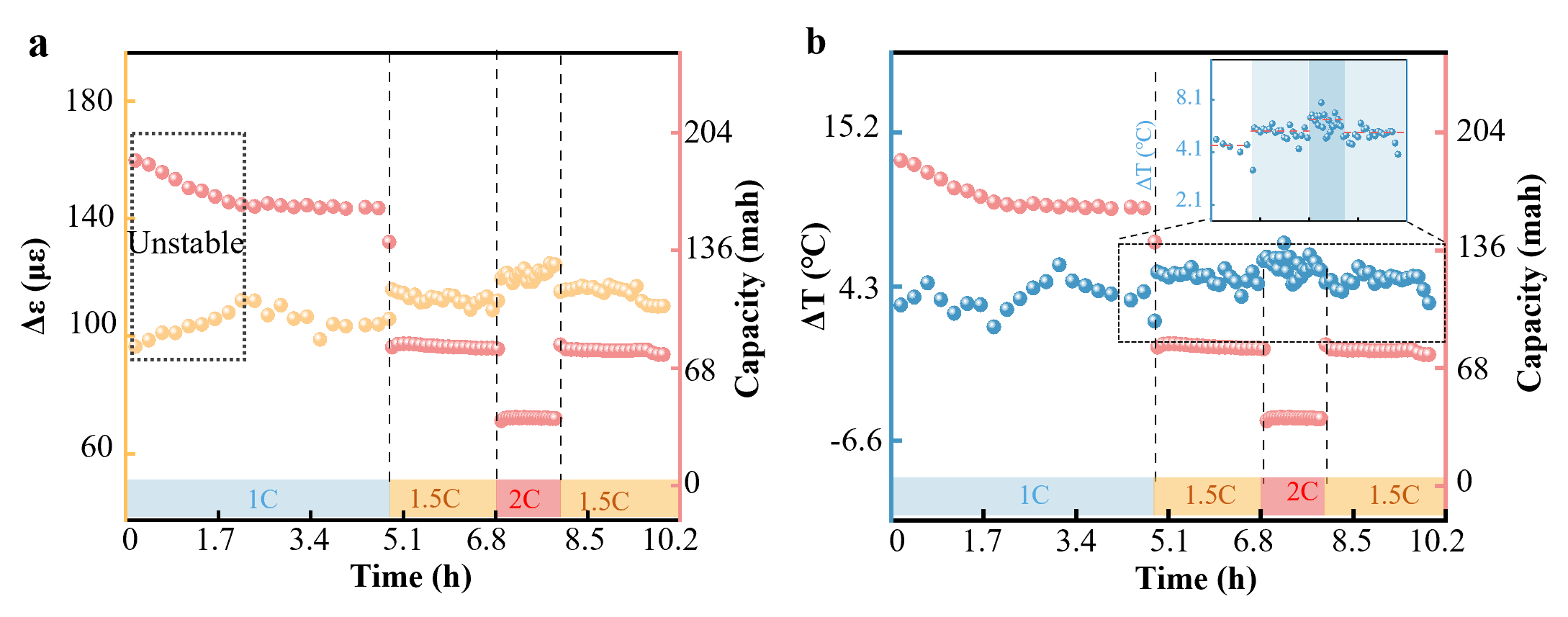

Supplement: Supplementary 1 — Figs. S1 to S28 Tables S1 to S5 Movies S1 to S3 [file research.1120.f1.zip › Fig.S22.tif]

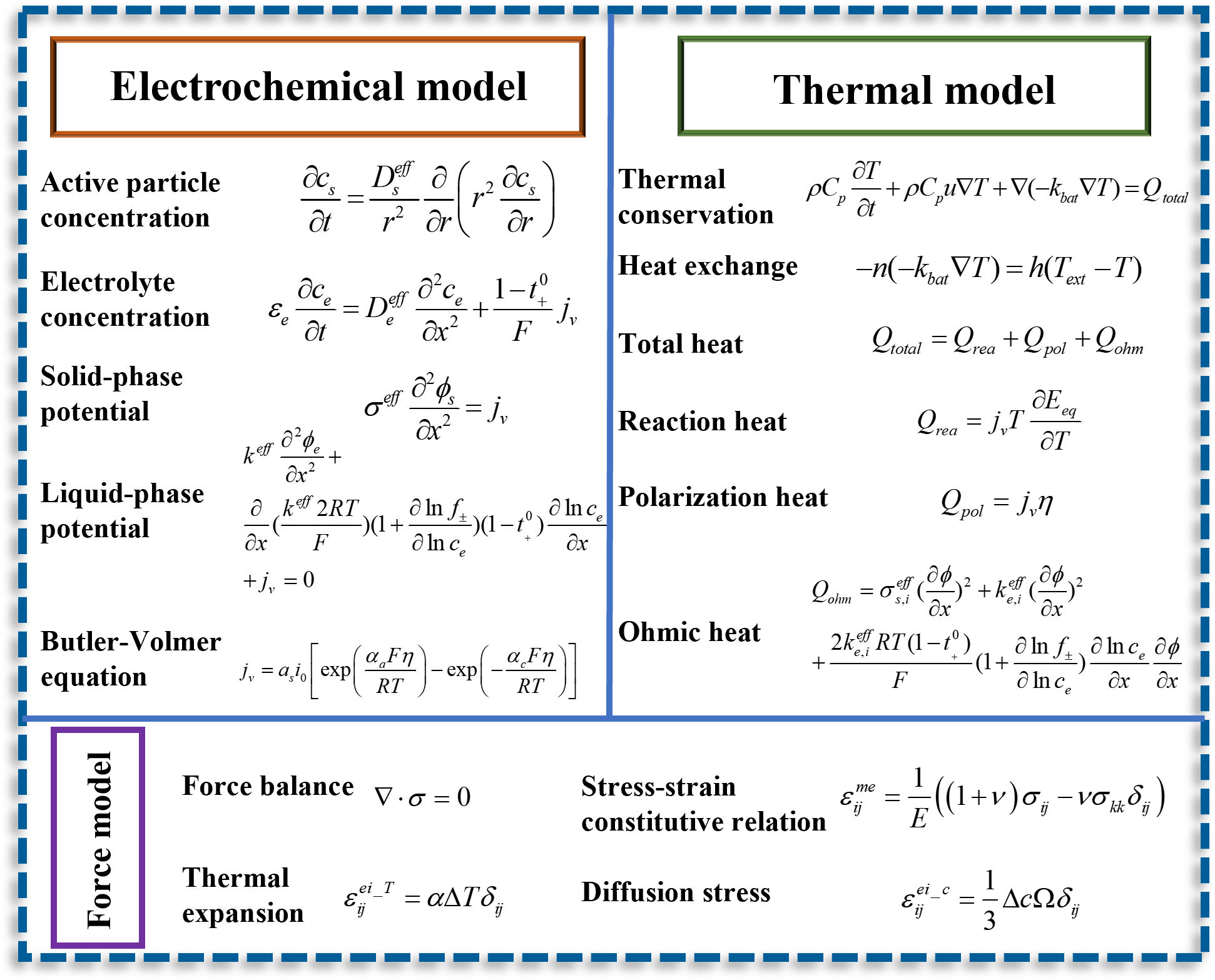

Supplement: Supplementary 1 — Figs. S1 to S28 Tables S1 to S5 Movies S1 to S3 [file research.1120.f1.zip › Fig.S23.tif]

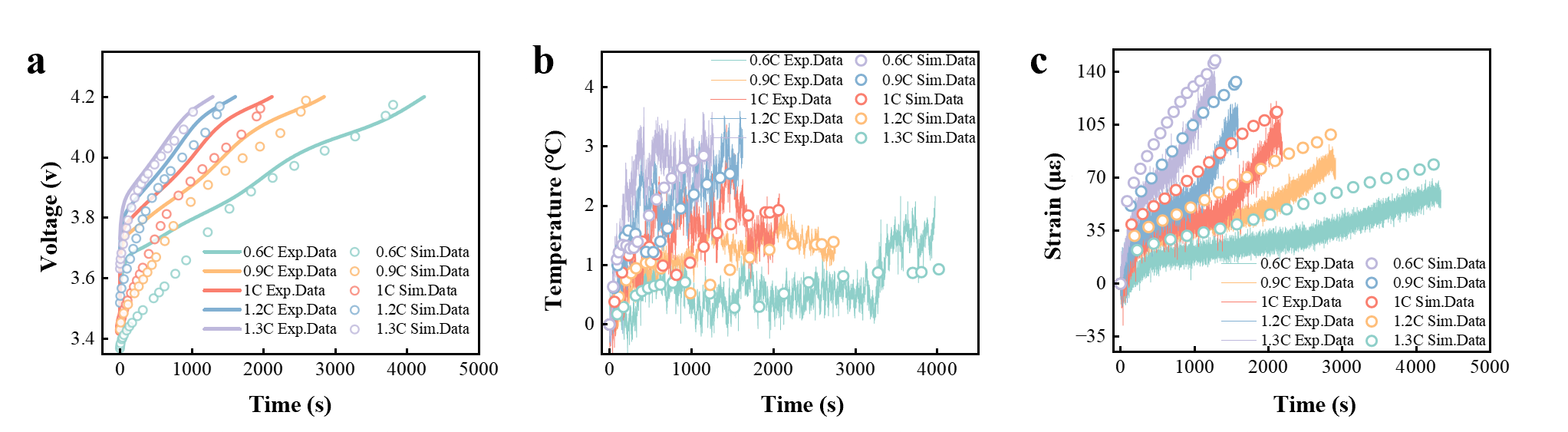

Supplement: Supplementary 1 — Figs. S1 to S28 Tables S1 to S5 Movies S1 to S3 [file research.1120.f1.zip › Fig.S24.png]

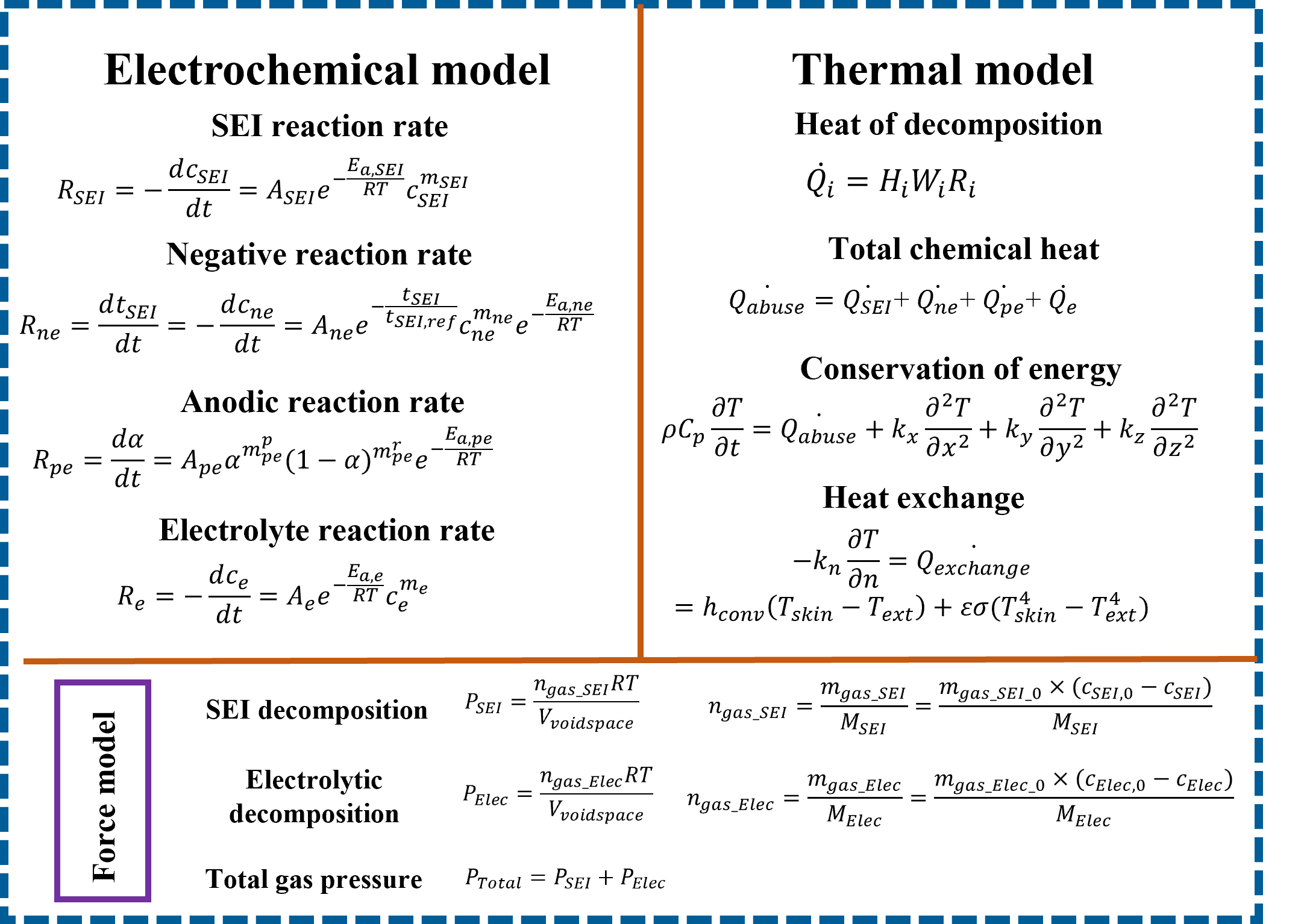

Supplement: Supplementary 1 — Figs. S1 to S28 Tables S1 to S5 Movies S1 to S3 [file research.1120.f1.zip › Fig.S24.tif]

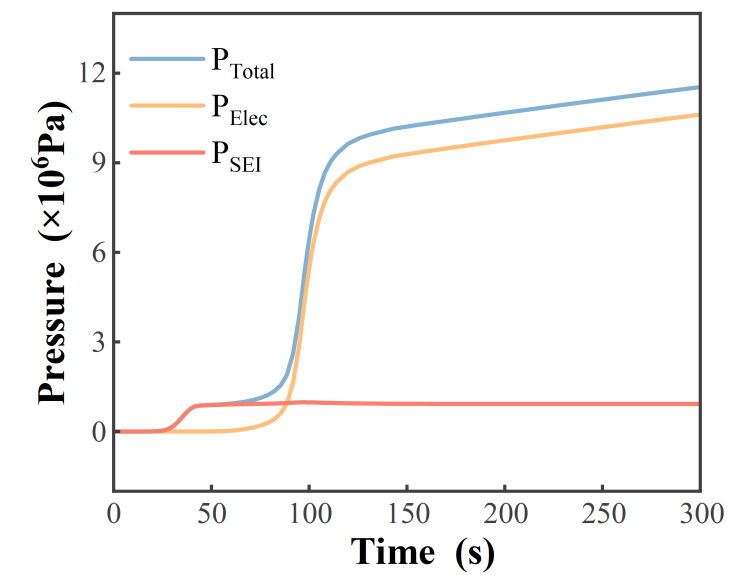

Supplement: Supplementary 1 — Figs. S1 to S28 Tables S1 to S5 Movies S1 to S3 [file research.1120.f1.zip › Fig.S25.tif]

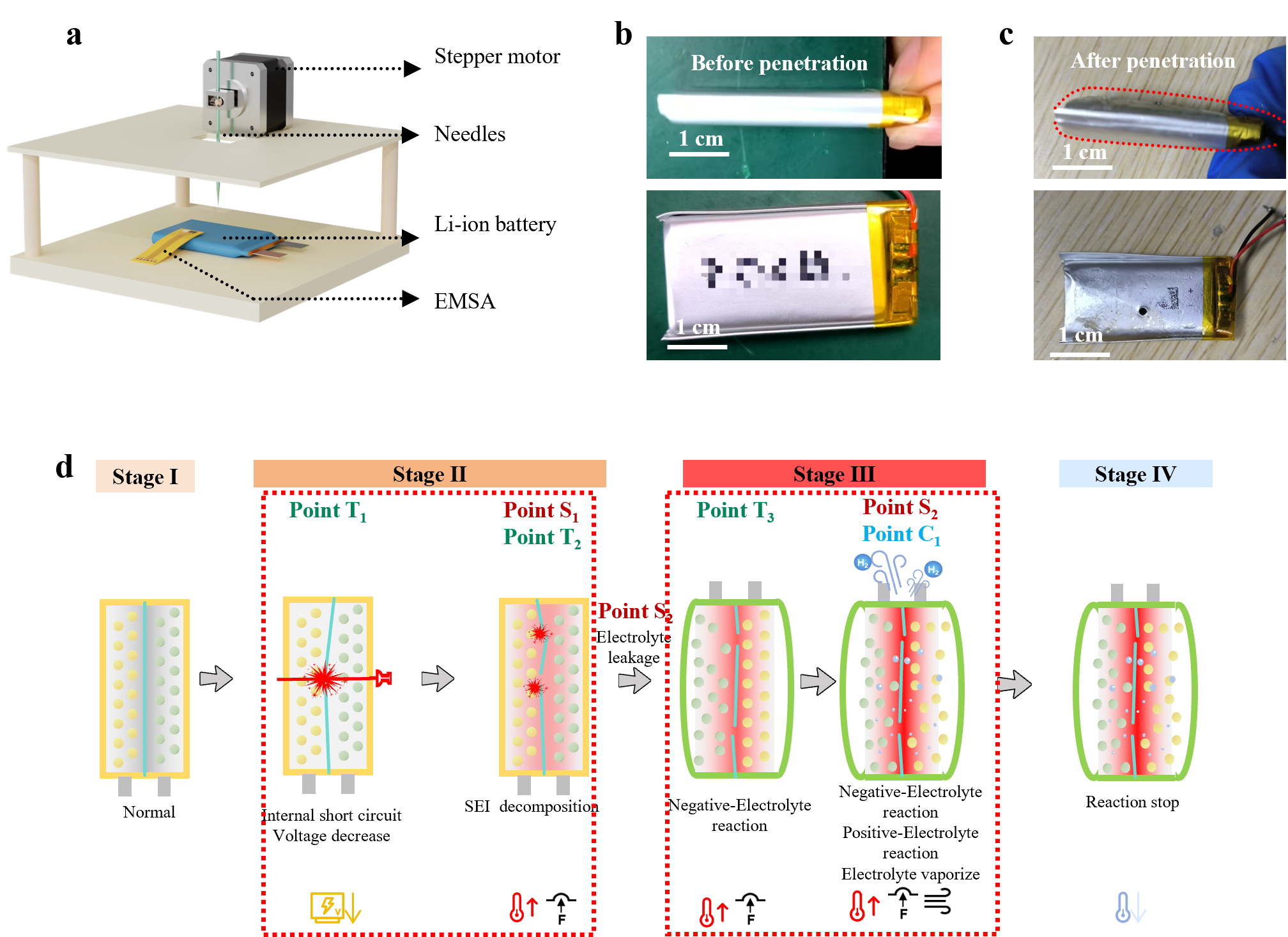

Supplement: Supplementary 1 — Figs. S1 to S28 Tables S1 to S5 Movies S1 to S3 [file research.1120.f1.zip › Fig.S26.tif]

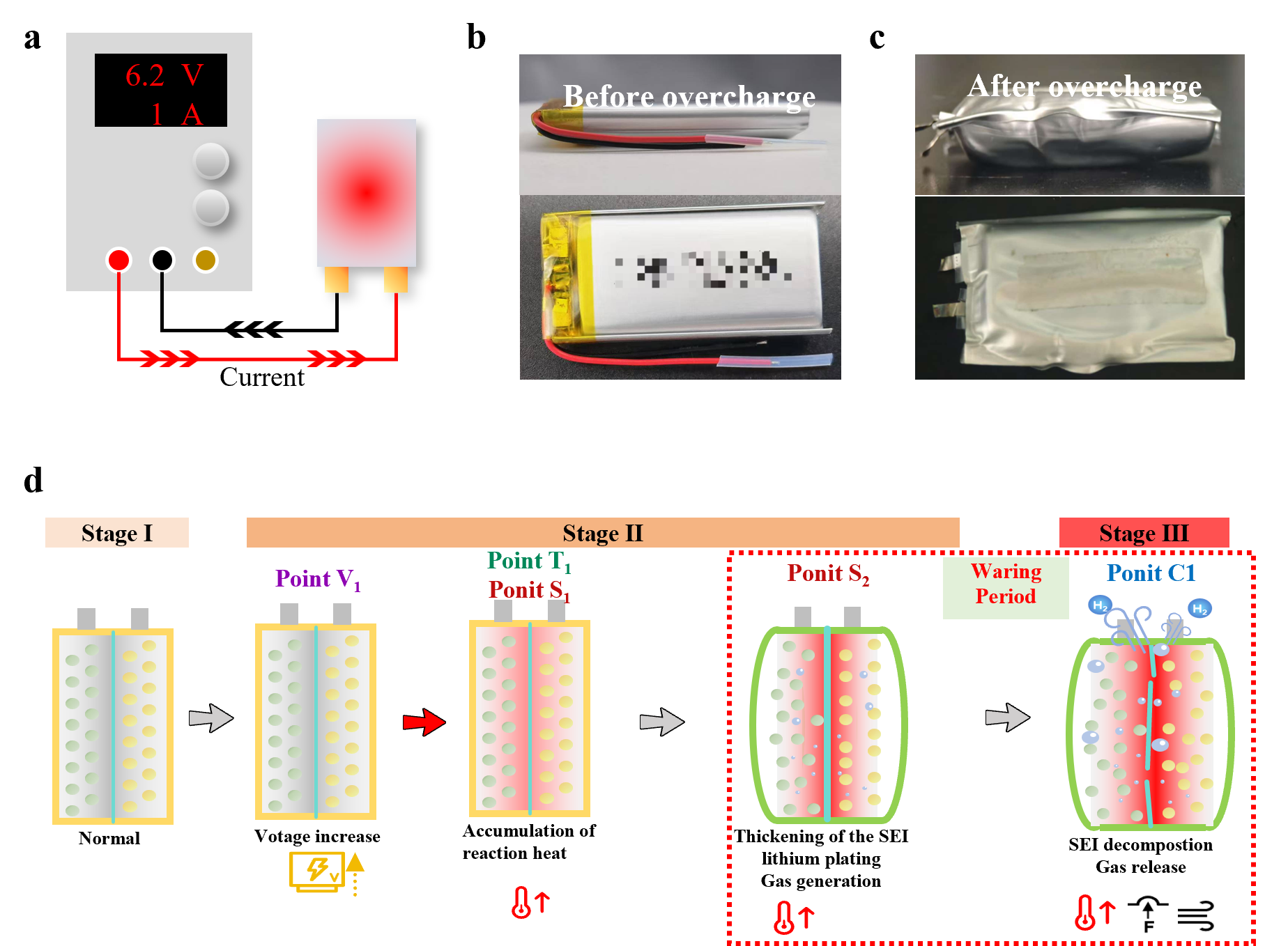

Supplement: Supplementary 1 — Figs. S1 to S28 Tables S1 to S5 Movies S1 to S3 [file research.1120.f1.zip › Fig.S27.tif]

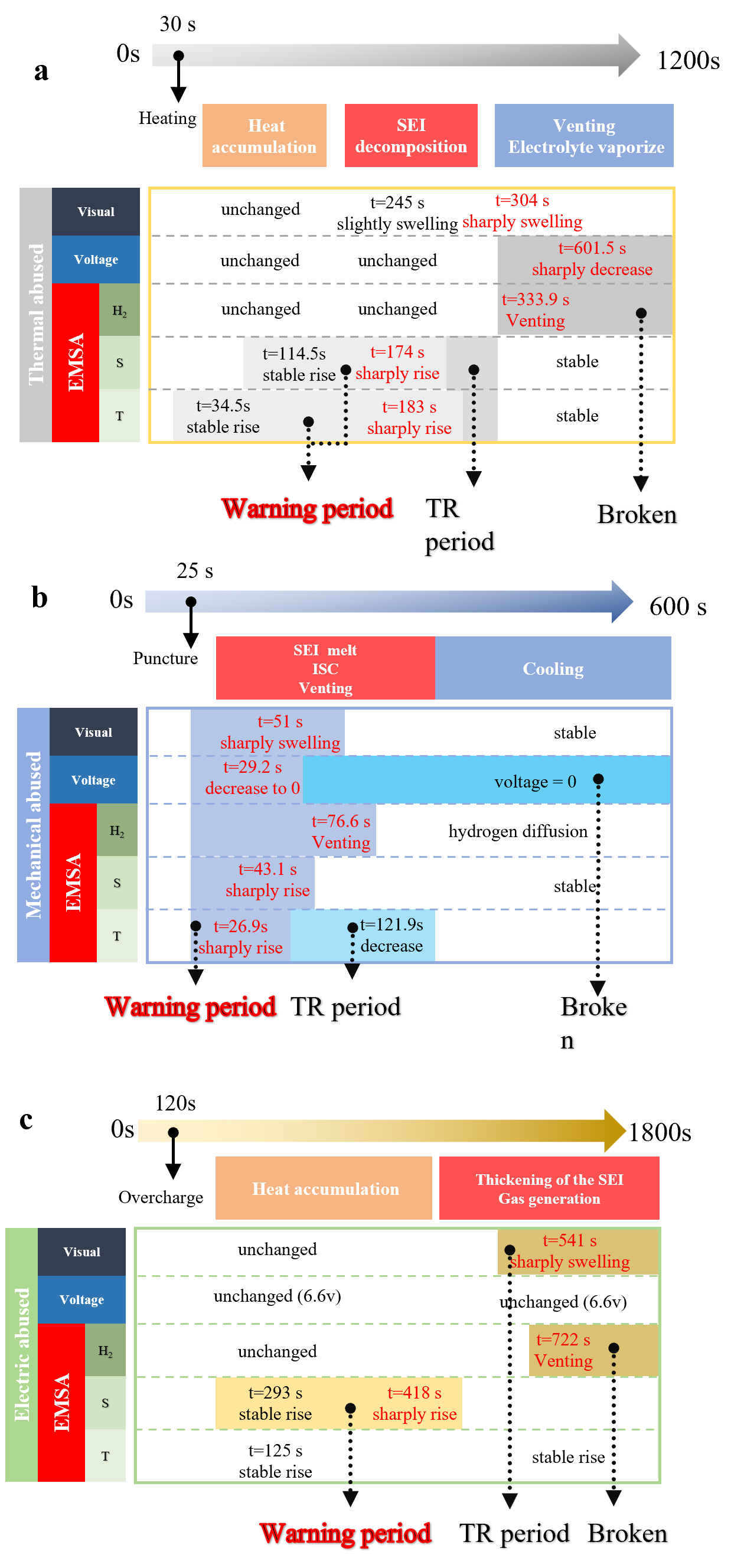

Supplement: Supplementary 1 — Figs. S1 to S28 Tables S1 to S5 Movies S1 to S3 [file research.1120.f1.zip › Fig.S28.tif]

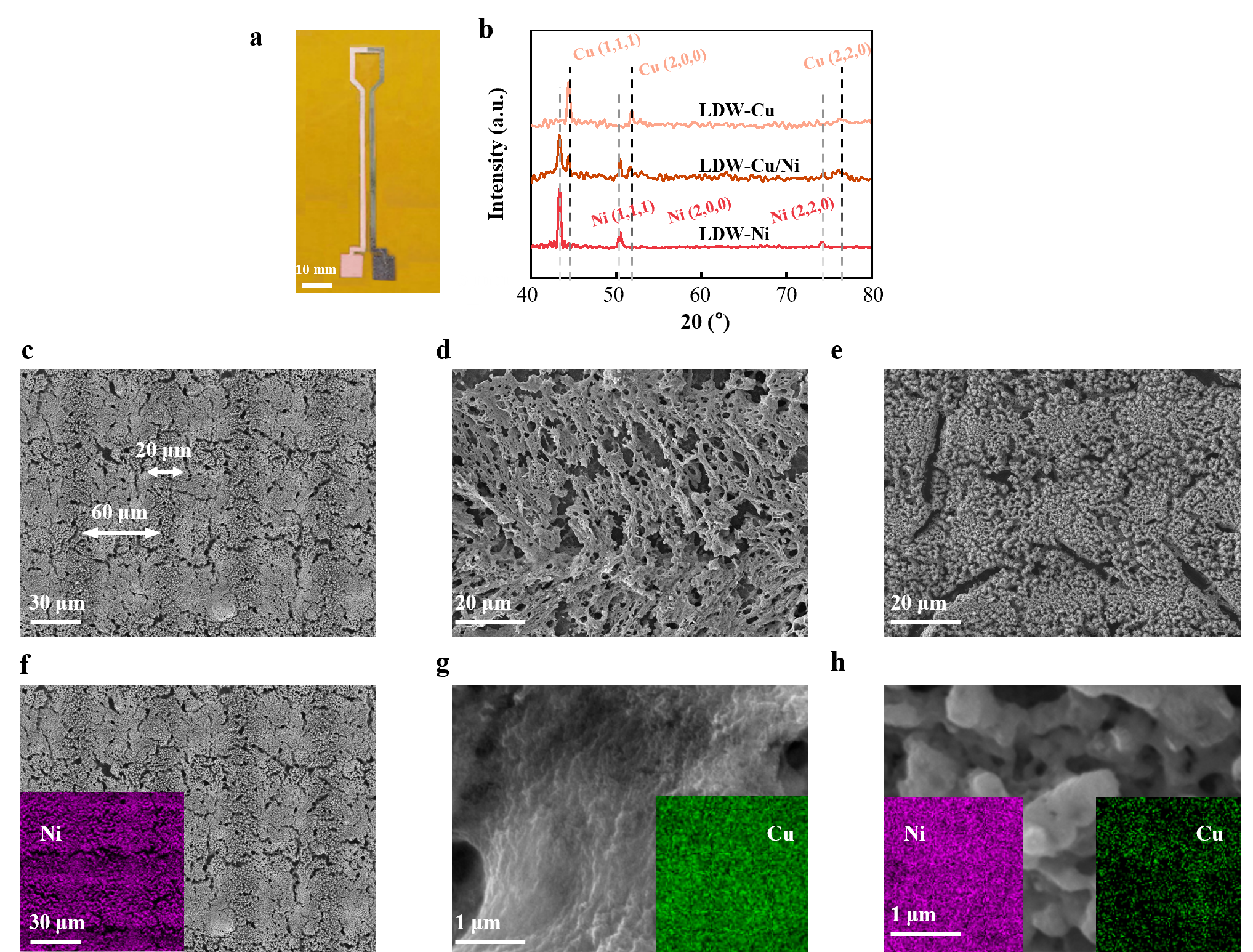

Supplement: Supplementary 1 — Figs. S1 to S28 Tables S1 to S5 Movies S1 to S3 [file research.1120.f1.zip › Fig.S3.tif]

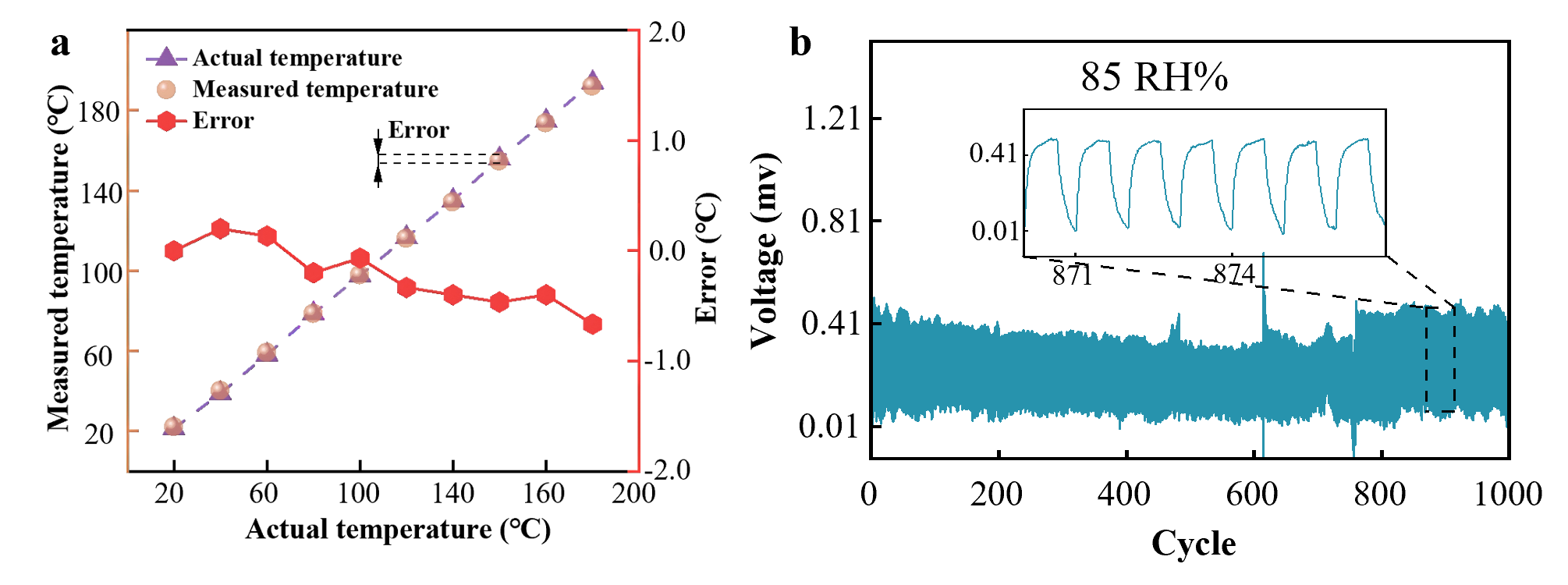

Supplement: Supplementary 1 — Figs. S1 to S28 Tables S1 to S5 Movies S1 to S3 [file research.1120.f1.zip › Fig.S4-2.png]

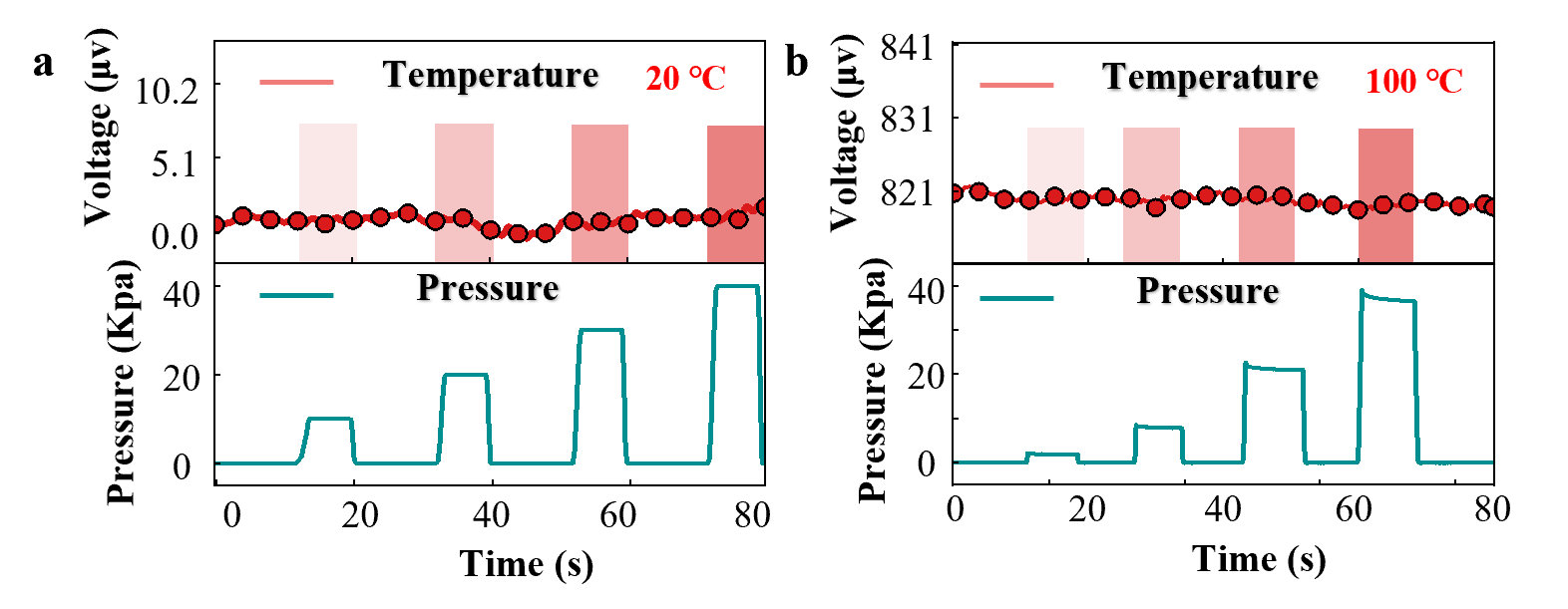

Supplement: Supplementary 1 — Figs. S1 to S28 Tables S1 to S5 Movies S1 to S3 [file research.1120.f1.zip › Fig.S5.tif]

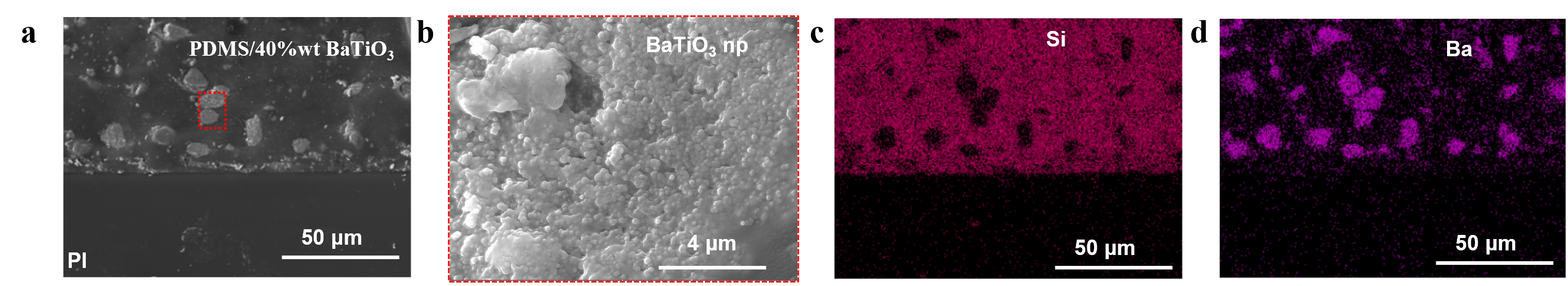

Supplement: Supplementary 1 — Figs. S1 to S28 Tables S1 to S5 Movies S1 to S3 [file research.1120.f1.zip › Fig.S7.png]

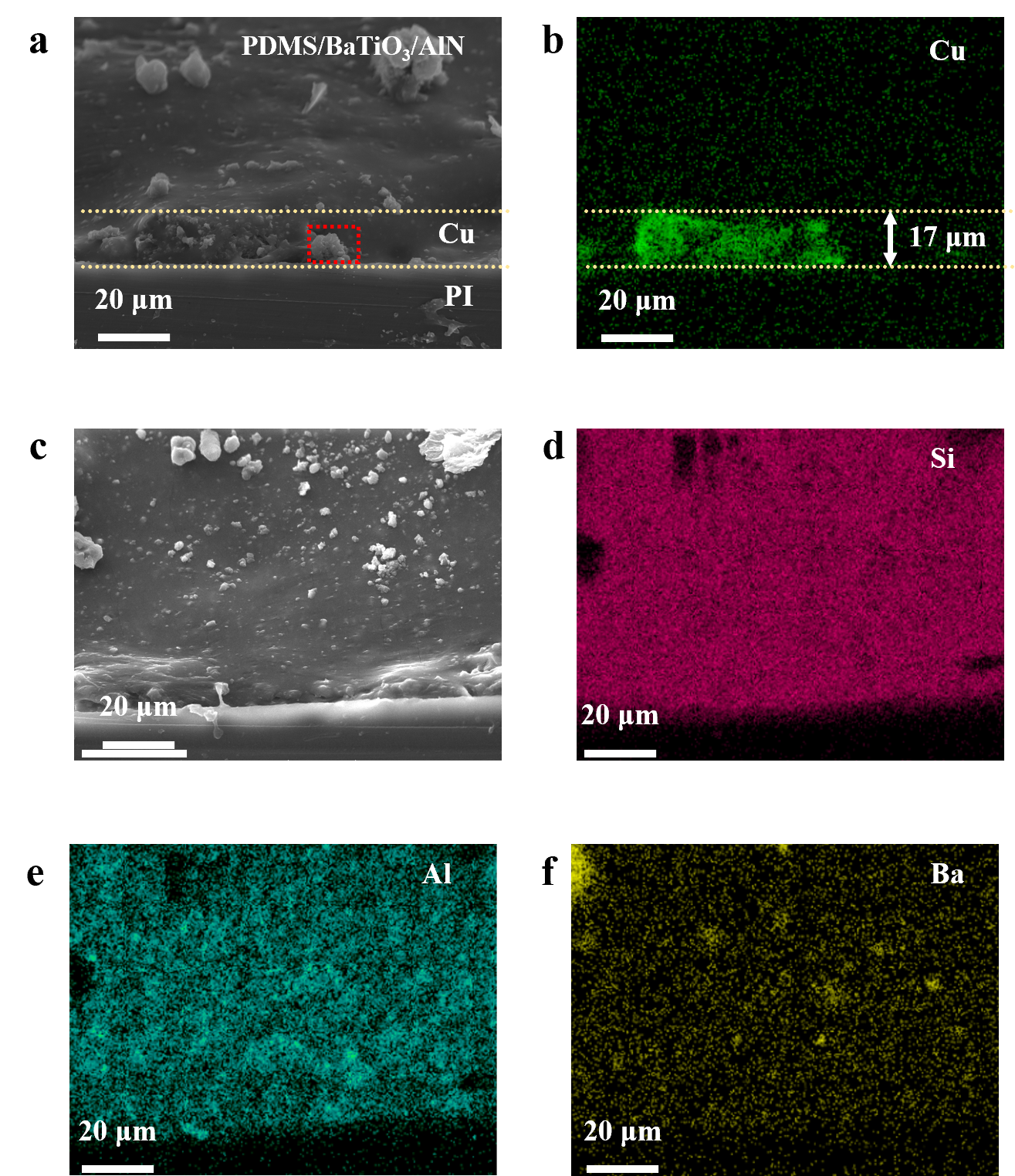

Supplement: Supplementary 1 — Figs. S1 to S28 Tables S1 to S5 Movies S1 to S3 [file research.1120.f1.zip › Fig.S8.tif]

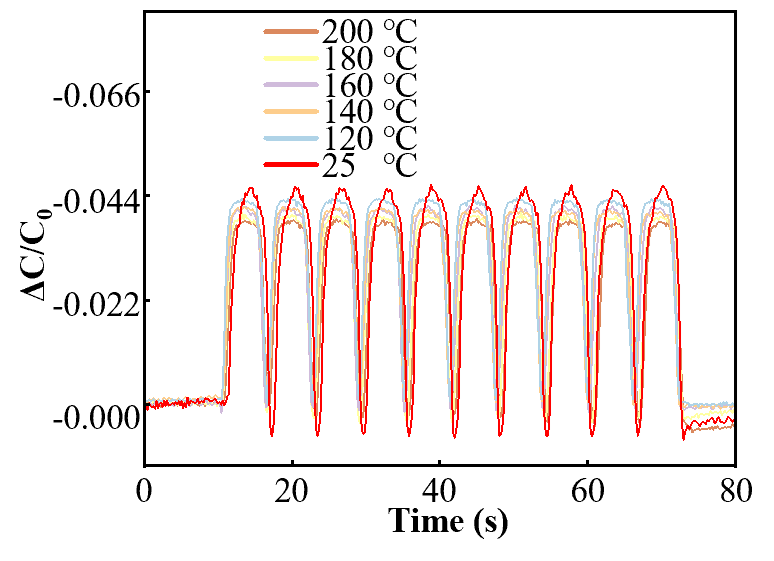

Supplement: Supplementary 1 — Figs. S1 to S28 Tables S1 to S5 Movies S1 to S3 [file research.1120.f1.zip › Fig.S9.tif]

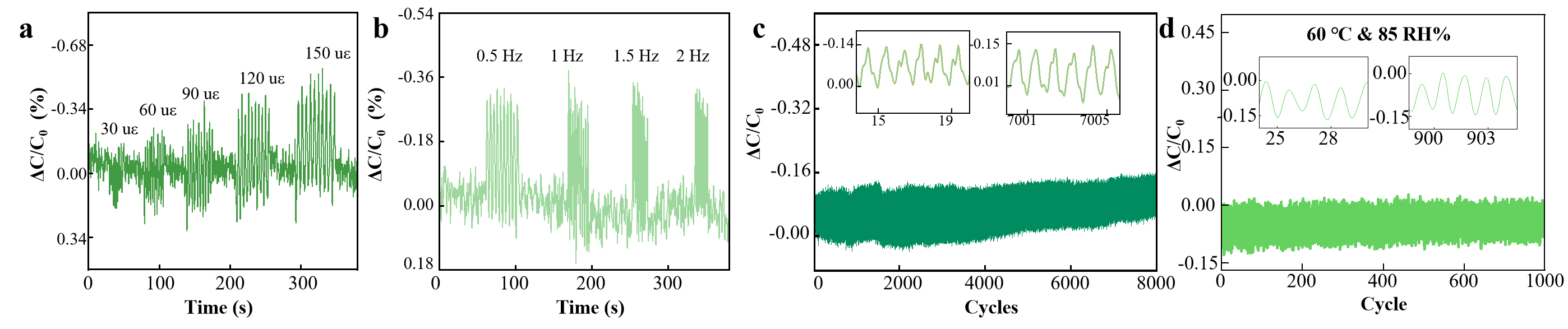

Supplement: Supplementary 1 — Figs. S1 to S28 Tables S1 to S5 Movies S1 to S3 [file research.1120.f1.zip › FigS11.png]

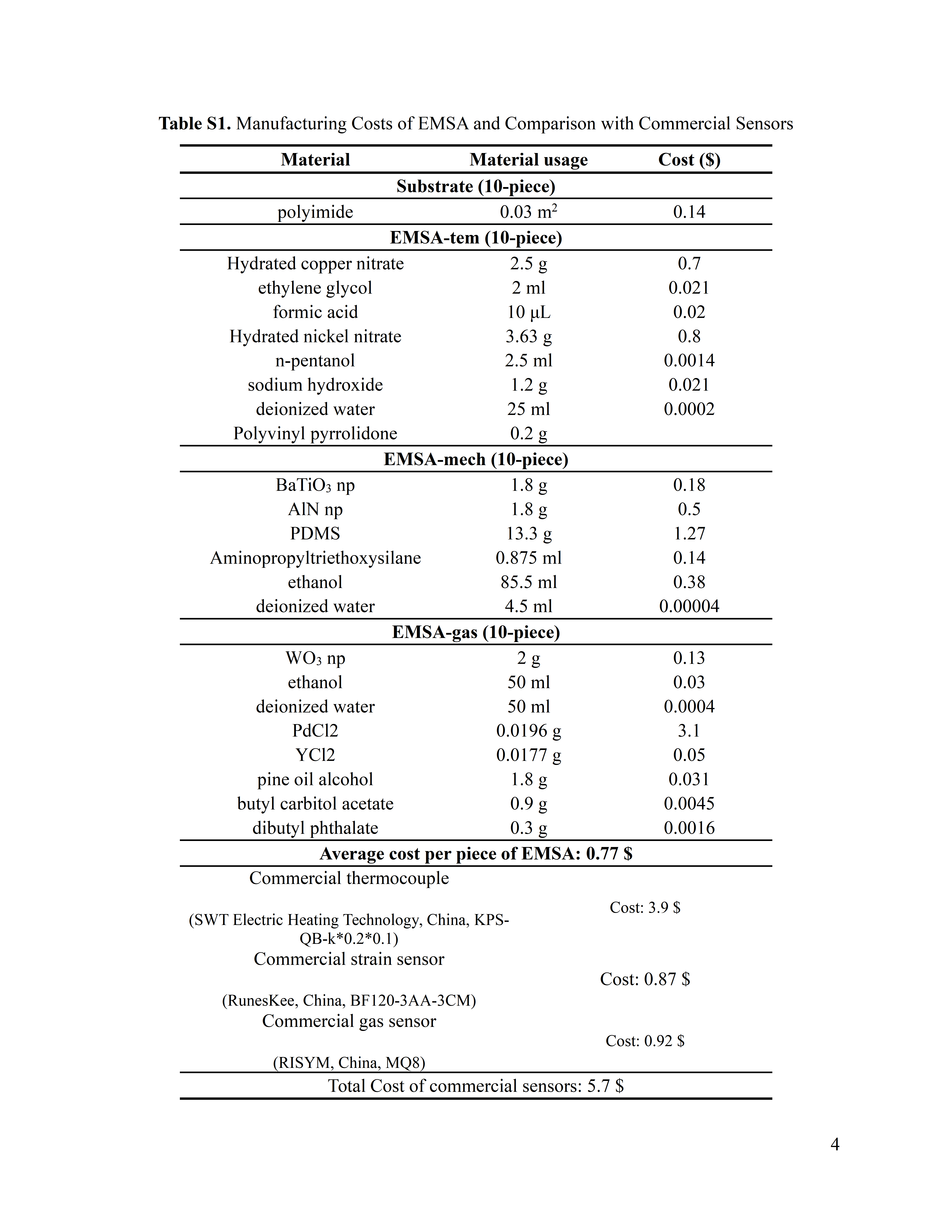

Supplement: Supplementary 1 — Figs. S1 to S28 Tables S1 to S5 Movies S1 to S3 [file research.1120.f1.zip › Table S1.png]

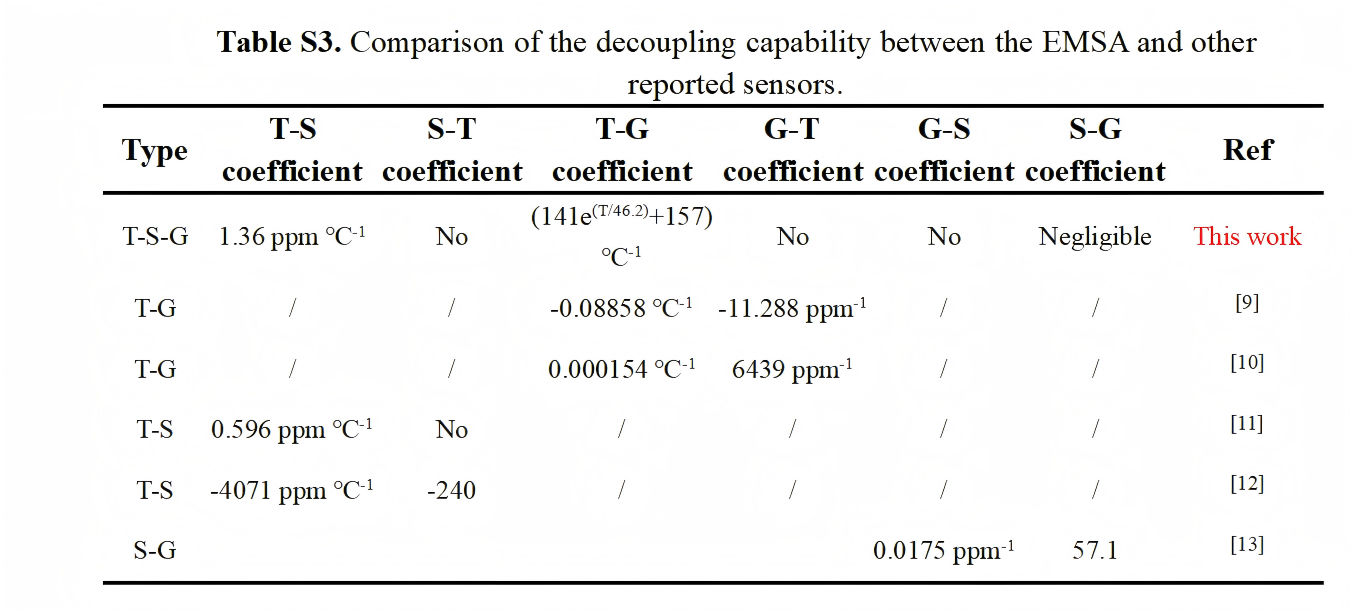

Supplement: Supplementary 1 — Figs. S1 to S28 Tables S1 to S5 Movies S1 to S3 [file research.1120.f1.zip › Table S3.png]

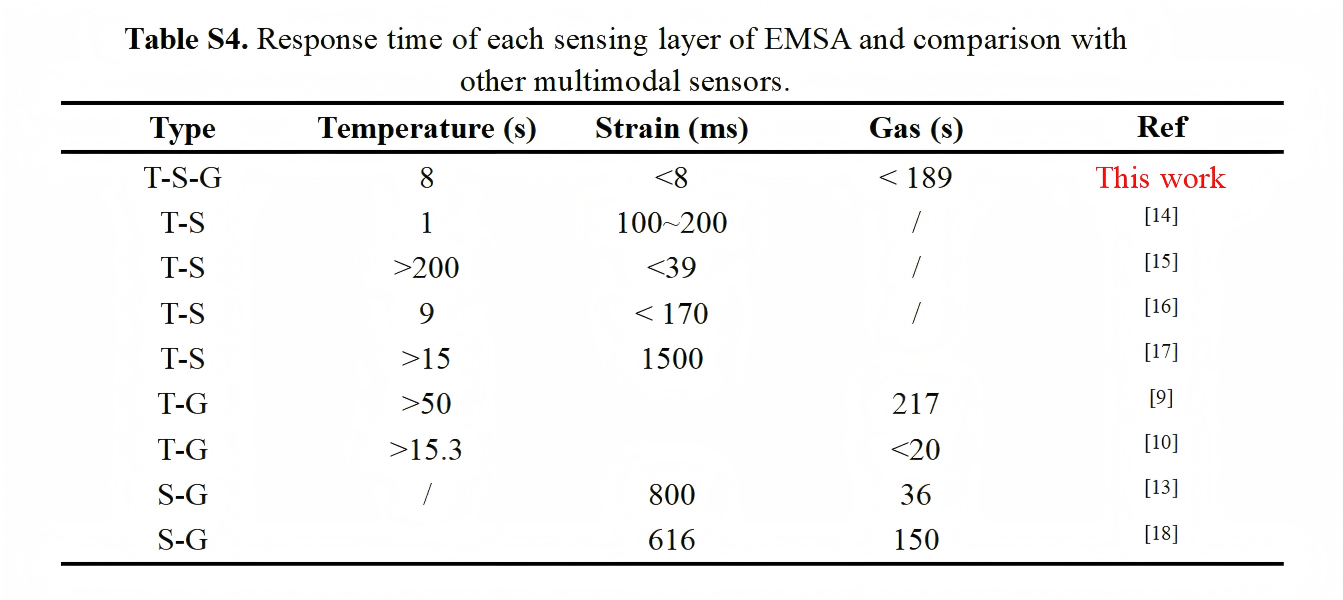

Supplement: Supplementary 1 — Figs. S1 to S28 Tables S1 to S5 Movies S1 to S3 [file research.1120.f1.zip › Table S4.png]

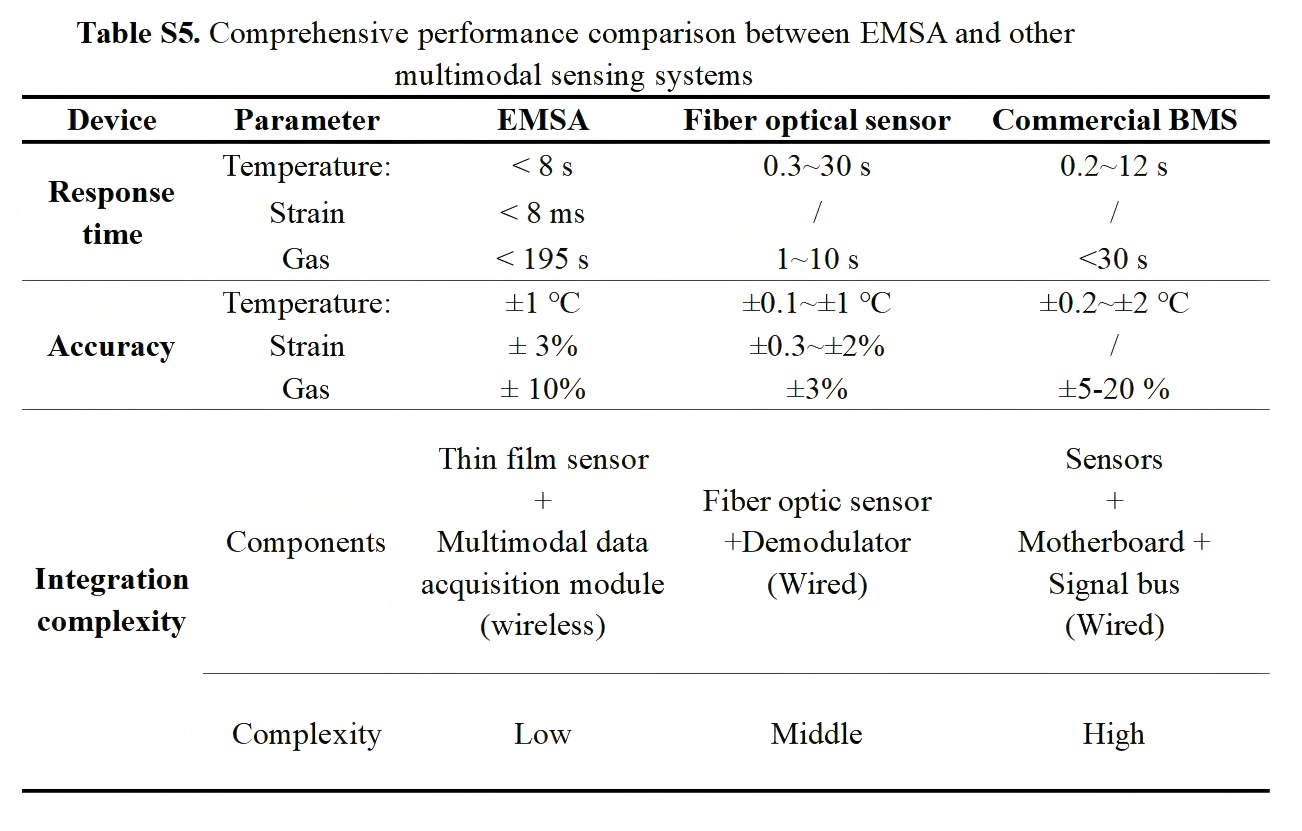

Supplement: Supplementary 1 — Figs. S1 to S28 Tables S1 to S5 Movies S1 to S3 [file research.1120.f1.zip › Table S5.png]

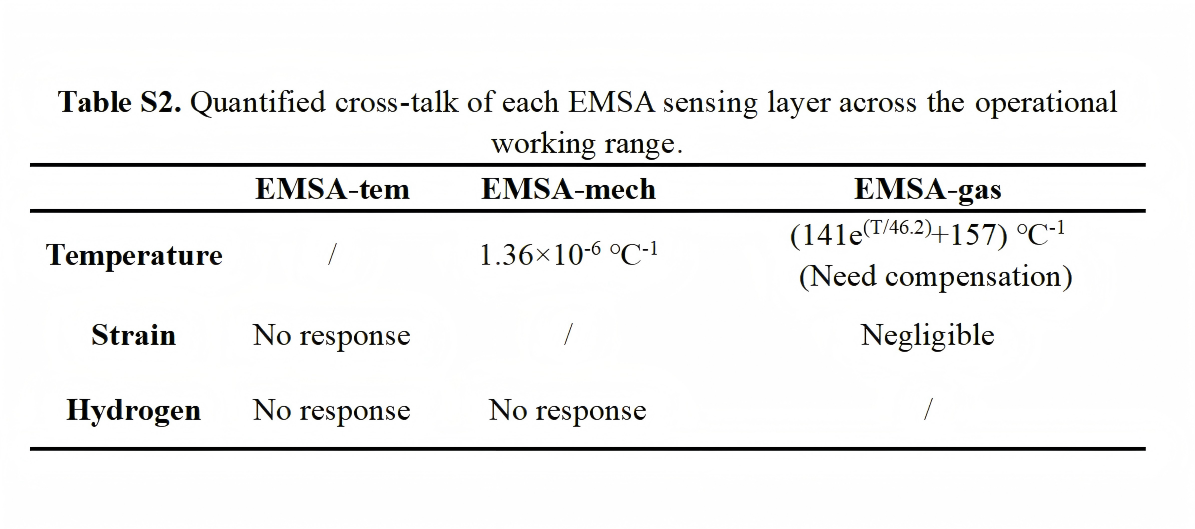

Supplement: Supplementary 1 — Figs. S1 to S28 Tables S1 to S5 Movies S1 to S3 [file research.1120.f1.zip › TableS2.png]
